# Screening of amphipathic helices identifies features linked to inner nuclear membrane properties

**DOI:** 10.1038/s41467-026-75561-0

**Published:** 2026-07-30

**Authors:** Shoken Lee, Anabel-Lise Le Roux, Marc Goudge, Mira Mors, Stefano Vanni, Pere Roca‑Cusachs, Shirin Bahmanyar

**Affiliations:** 1 Department of Molecular Cellular and Developmental Biology, Yale University, New Haven, CT USA; 2 Institute for Bioengineering of Catalonia, The Barcelona Institute of Technology (BIST), Barcelona, Spain; 3 Department of Biology, University of Fribourg, Fribourg, Switzerland; 4 Swiss National Center for Competence in Research Bio-Inspired Materials, University of Fribourg, Fribourg, Switzerland; 5 Departament de Biomedicina, Unitat de Biofísica i Bioenginyeria, Facultat de Medicina i Ciències de la Salut, Universitat de Barcelona, Barcelona, Spain; 6 Present Address: Department of Microbiology and Immunology, Emory University School of Medicine, Atlanta, GA USA

**Keywords:** Nucleus, Membrane lipids, Membrane biophysics, Molecular modelling

## Abstract

Inner nuclear membrane (INM) proteins control numerous nuclear functions, yet properties governing peripheral INM-association remain poorly defined. Here, we perform an image-based screen to identify features in candidate and established amphipathic helices (AHs) that mediate INM association. AHs that localize to ER/Golgi membranes become INM-associated when directed to the nucleus, whereas mitochondrial-localized AHs become largely nucleoplasmic. Mutating a mitochondrial-localized AH to increase its preference for membranes with lipid packing defects enables INM-association upon nuclear targeting. Structural studies of an INM-associated AH in TMEM214 show folding upon binding to lipid packing defects, and full-length TMEM214 localizes to nuclear pores. Consistently across multiple AHs, INM binding depends primarily on sensitivity to lipid packing defects, with a minor electrostatic contribution. Highlighting distinct effects of different mechanical inputs, nuclear swelling, but not cell stretching, enhances INM association of select AHs. These findings define AH features that promote INM association, with implications for nucleo-mechanical responses.

## Abstract (web summary)

The properties of the nuclear envelope and mechanisms of peripheral protein association are poorly understood. Here, authors show sensing lipid packing defects and a minor electrostatic contribution governs amphipathic helix binding, with links to nuclear responses to mechanical stimuli.

## Introduction

Membrane-bound organelles have characteristic biophysical properties defined by their distinct lipid compositions. The endoplasmic reticulum (ER) and Golgi apparatus are defined by loose lipid packing and low electrostatics, whereas organelles of the late secretory pathway, plasma membrane, and mitochondria are defined by tight lipid packing and a negative surface charge^1–3^.

Protein regions called amphipathic helices (AHs) recognize differences in membrane properties to anchor soluble proteins to, and in some cases alter the local shape of, the targeting organelle^2,4,5^. AHs reversibly insert in the polar–apolar region of a single leaflet of a lipid bilayer^6^. The selectivity of AHs for specific membrane properties depends on the composition of their charged and hydrophobic amino acid residues on each face of the helix^4,5^. This composition determines the relative contributions of electrostatics and the hydrophobic effect for membrane binding^6,7^. Some AHs named ALPS motifs (e.g., AH Lipid-Packing Sensors) with no to very few charged amino acids on their polar face minimize the electrostatic component to mainly depend on the hydrophobic effect for membrane partitioning^8–11^. This property along with a low density of hydrophobic residues prevents binding to flat membranes and makes them highly sensitive to curved membranes that intrinsically contain lipid packing defects^4,11–13^. Packing defects are regions in which hydrophobic acyl tails of phospholipids are exposed to the aqueous cytoplasm, a property typical of curved membranes as well as loosely packed bilayers.

Although less characterized, AHs also exist in integral membrane proteins. Membrane shaping proteins of the integral family of reticulons and REEPs contain AHs that help generate curved ER tubules^14,15^. An AH in the membrane protein IRE1 detects membrane stress to help trigger the unfolded protein response (UPR)^16^. Thus, AHs are not exclusive to soluble proteins; they also exist in membrane proteins to carry out organelle-specific functions.

While the membrane properties of most organelles have been characterized, less is known about the nuclear envelope (NE) that surrounds the genome^17^. The NE is continuous with the ER, making it challenging to biochemically define its lipid content in isolation^17^. The chromatin-facing inner nuclear membrane (INM) is enriched with a subset of membrane proteins that have a variety of functions^18^. The INM is fused to the outer nuclear membrane (ONM) at regions of high curvature embedded with nuclear pore complexes (NPCs), the gateway between the nucleoplasm and cytoplasm^19,20^. AHs in some nucleoporins help bind and shape the curved nuclear pore membrane and guide targeting to the NPC^19,21–27^. Although few in number, examples of AHs in NE-associated proteins other than nucleoporins^28–31^, suggest that lipid-protein interactions are likely a key missed feature important to INM functions.

The response of the NE to mechanical forces is one context in which lipid-protein interactions likely play a critical role. For example, INM-association of the nuclear-localized enzyme cPLA2, which binds lipid packing defects through its C2 domain, is induced by mechanical forces on the nucleus, leading to downstream lipid signaling that changes cell contractility^32,33^. In general, mechanical forces imposed on the NE have profound effects on cell functions and fate^32,34–38^, and tension is known to influence membrane properties^39–41^, highlighting the need to gain foundational knowledge of the biophysical properties of the INM, as determined by its lipid composition.

Lipid biosensors developed for the INM in mammalian cells have revealed the presence of the conical lipid DAG^31^ as well as the negatively charged lipid PS^42^. The presence of DAG in flat membranes increases the probability of packing defects, which resemble those induced by positive curvature^11,43^, while PS enhances the binding of cationic peptides and proteins. However, it is not possible to infer INM biophysical membrane properties with lipid biosensors alone because of the inability to gain quantitative data of absolute or even relative concentrations of the lipids they detect.

Here, we performed a screen of candidate AHs from putative integral membrane NE and ER proteins to relate amino acid sequence features to their cytosolic organelle localization when expressed in independent of their protein backbone. Nuclear targeting of 32 AH candidates shows that AHs localizing to ER/Golgi membranes—but not those restricted to mitochondria—also associate with the INM, indicating shared features between ER/Golgi- and INM-associated AHs. Consistent with this, mutating charged amino acid residues on the hydrophilic face of the mitochondrial-localized AH from TMEM126A shifts its localization toward ER/Golgi membranes, enables INM association upon nuclear targeting, and promotes binding to membranes with lipid packing defects in vitro. Analysis of candidate and established AHs further supports a role for sensitivity to lipid packing defects over electrostatics in INM association. A subset of AHs, including ALPS motifs, increase their INM association upon nuclear swelling, but not in response to cell stretch, suggesting that distinct mechanical inputs may have different effects on the properties of the INM. Finally, we identify TMEM214 as a nuclear pore–associated protein containing an AH that folds upon binding to lipid packing defects, validating that our screening approach uncovered a lipid-binding AH in a NE-associated protein.

## Results

### Generating a list of candidate AHs in membrane proteins

**Fig. 1:**
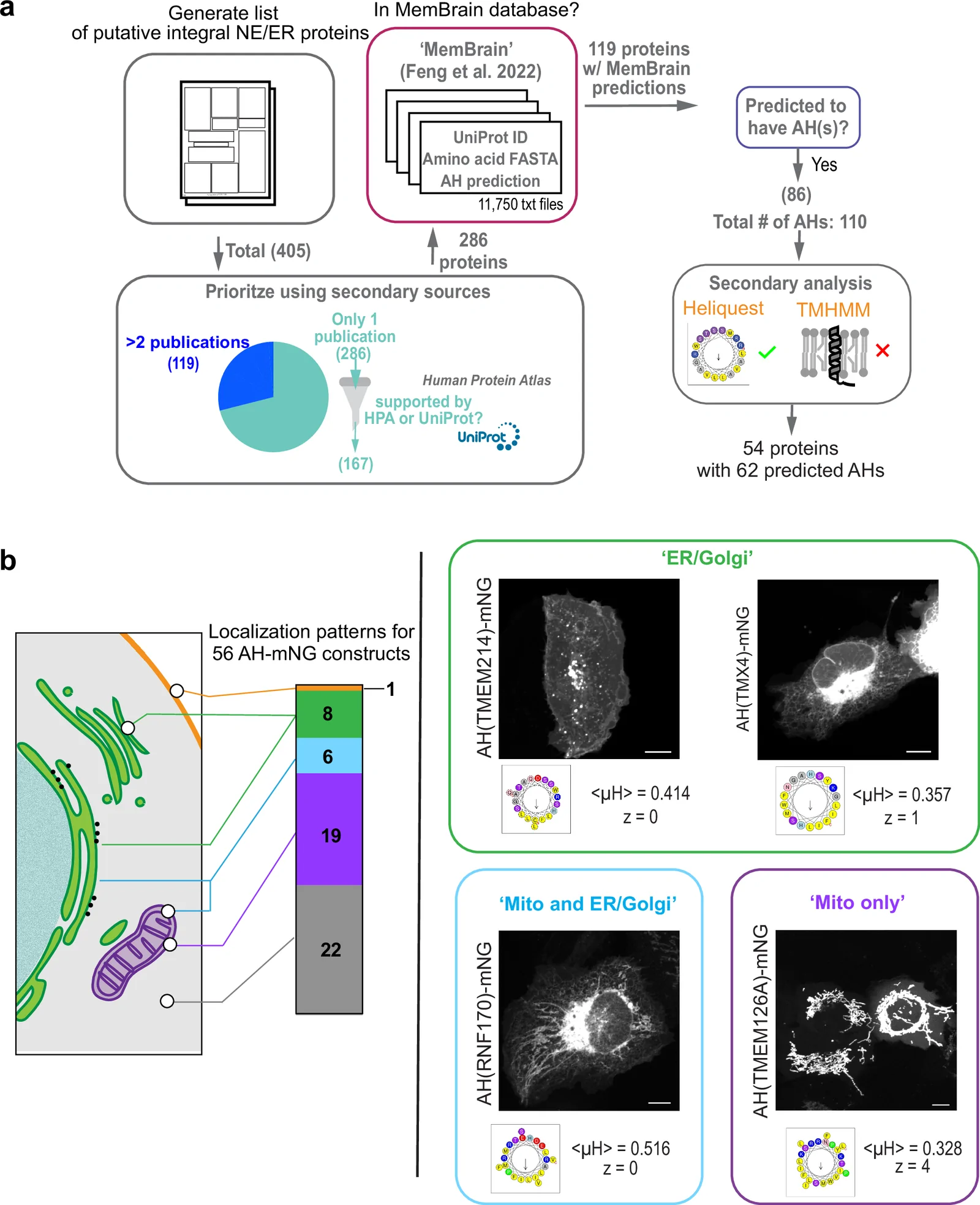
Identification of amphipathic helices (AHs) in membrane proteins and their localization in the cytosol, independent of their protein backbone. **a** Flow chart of data mining for putative membrane proteins with some evidence for association with the nuclear envelope (NE). Secondary identification of predicted AHs. See Fig. S1 for more details. **b** Plot and corresponding color-coded schematic summarizing localization categories for AH-mNG in U2OS cells expressed independent of backbone in the cytosol. Representative spinning disk confocal images of U2OS cells expressing the indicated constructs. Experiments were conducted two or three times with similar results. The wheel projections of the predicted AHs with mean hydrophobic moment <µH> and net charge z is shown. See Figs. S6–S8 for representative images of remaining AH-mNG candidates. Scale bars, 10 µm.

Our prior work used machine learning-based algorithms to yield seven predicted AHs in seven established INM proteins^31^. We increased the number of putative NE-associated proteins to 405 by compiling data from biochemical studies in which the NE proteome was determined^44–48^, with the understanding that these data will not be exclusive to the INM because of the challenges of isolating the NE from the rest of the ER (Fig. 1a; see Fig. S1A, B and “Methods” for methodological details, see Supplementary Data 1–6). We refined this list to 286 proteins by prioritizing those in which more than one publication supported some NE localization or additional support for nuclear localization was available from the Human Protein Atlas or UniProt (Fig. S1A and “Methods”; see Supplementary Data 7 and 8).

We leveraged an in silico deep-learning-based algorithm called MemBrain to predict AHs, which the developers applied specifically to transmembrane domain (TMD)-containing proteins^49^. We filtered their published predictions that covered 11,750 putative transmembrane proteins from various organisms, hereafter referred to as the MemBrain list, to only those from humans (Fig. S1A and Supplementary Data 9 and 10). Merging the compiled list of putative NE proteins with the filtered MemBrain analysis resulted in 86 proteins that contained either one or two predicted AHs (Figs. 1, S1A and Supplementary Data 11 and 12).

Similar to our prior analyses of AHs^31^, AHs were identified from the MemBrain list in some characterized INM proteins (Fig. S1C). There was no overlap between the list of AH candidates from our study and the ALPS-like motifs identified in a prior proteome-wide bioinformatics screen^8^ (Fig. S2)— MemBrain analysis was not available for the only four proteins that overlapped between the two screens (Fig. S2). Potential false positives were eliminated if AH regions were predicted to be TMDs by TMHMM^50^. We also confirmed AHs by determining their amphipathicity in HeliQuest^51^ (Supplementary Data 13). Alphafold predictions are not in the membrane context, yet all of the candidate AHs on our list were predicted to be alpha-helical in the context of the full-length protein (Fig. S3)^52,53^. This was also the case for established AHs, except for ALPS motifs (Fig. S3). Note that we did not eliminate candidate AHs that appeared embedded in a structural domain, and so some of the predicted AHs may not be engaged in membrane interactions in the context of their protein backbone. Our final list included 54 proteins that carry 62 AH candidates, which was a manageable quantity that allowed for screening of membrane partitioning properties (Supplementary Data 14).

### Organelle preferences of candidate AHs

We set out to determine the membrane preferences of candidate AHs by expressing them in the cytosol independent of their protein context. Fifty-six AH candidates were appended to mNeonGreen (mNG) fluorescent protein at their C-termini, and their localization was assessed in living U2OS cells. Twenty-two candidate AHs were not membrane-bound (Figs. 1b and S4), and this was not because the mNG was abnormally cleaved from the peptide sequence (Fig. S5). Three main categories emerged from the localization analysis of the remaining 34 AHs: mitochondrial, ER and/or Golgi, or a mixture of ER/Golgi and mitochondrial membranes (Figs. 1b, S6–S8 and Supplementary Data 14). Note that only one AH (AH(Man1)) localized to the plasma membrane, but replacing a hydrophobic residue with a charged residue on the hydrophobic face did not alter its membrane association when targeted to the nucleus, while the same mutation for a different AH (AH(RNF160)) did, indicating that the amphipathic nature of the predicted AH of Man1 does not facilitate membrane binding (see Fig. S9A, B)^4^. We therefore eliminated this predicted AH from our analyses because other mechanisms (such as protein-protein interactions or covalent lipid modifications) are likely involved in its plasma membrane localization and further analyzed the remaining 33 AHs.

### Amino acid compositions of AHs relate to organelle preferences

**Fig. 2:**
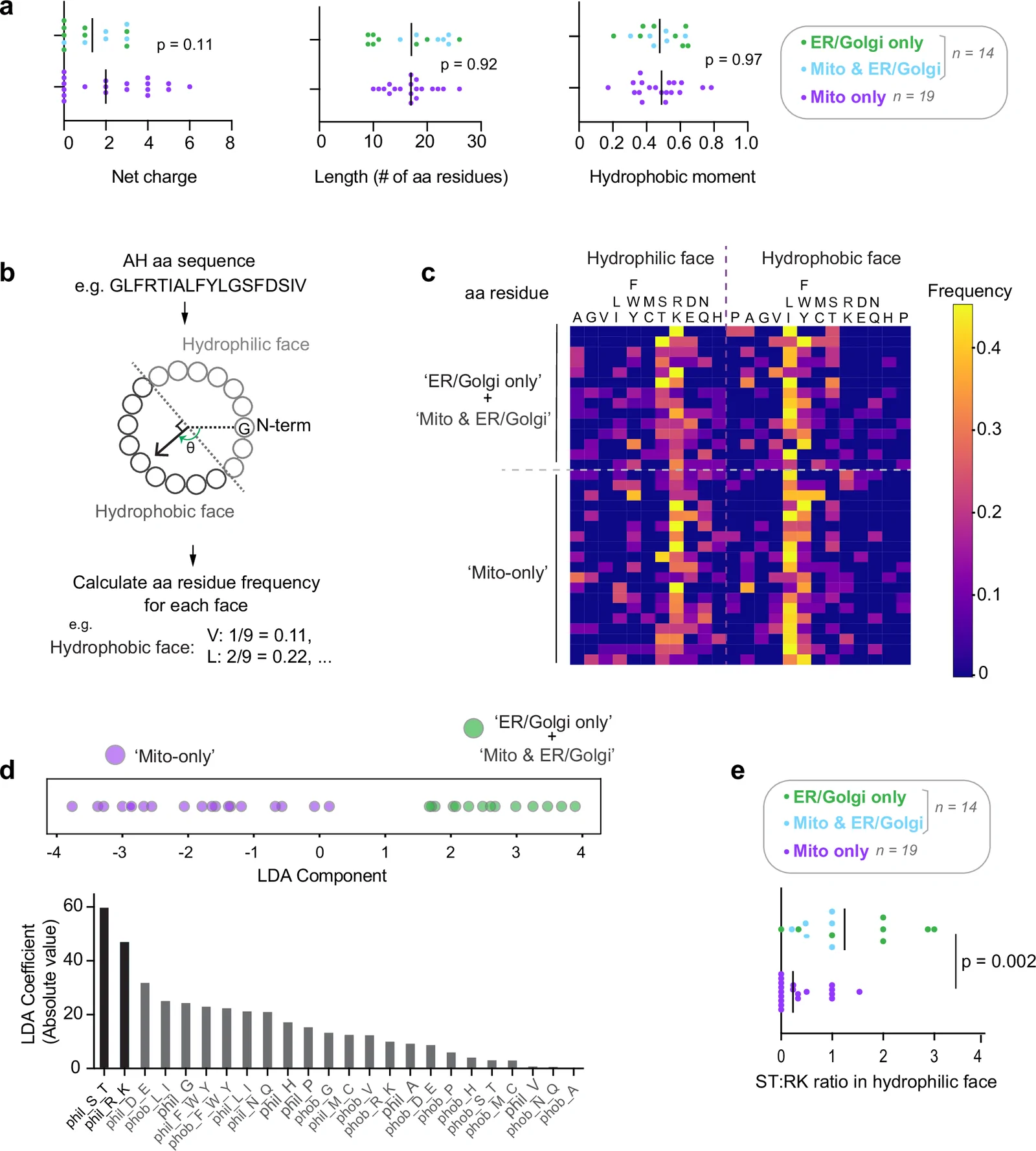
Analysis of amino acid sequences of candidate amphipathic helices (AHs) related to cytosolic localization categories. **a** Scatter plot of the indicated metrics for AH candidates categorized by localization (Fig. 1b). *n* = number of independent AH sequences. *P* values, unpaired two-sided *t* test. Data points represent independent AH sequences. **b** Flow chart of calculation of hydrophilic and hydrophobic faces and amino acid distribution. **c** Heatmap showing the fraction of amino acid residues with similar properties on each face of each AH. **d** Linear discriminant analysis (LDA) plot and corresponding coefficient contributions. **e** Scatter plot of the S/T:R/K ratio on the hydrophilic face for each AH. The bar represents median. *n* = number of independent AH sequences. *P* values, unpaired two-sided *t* test. Source data are provided.

We combined AHs that showed some localization to ER/Golgi membranes and compared their amino acid compositions to AHs that exclusively localized to mitochondria (mito-only) (Fig. 2a). We predicted that the mito-only localized AHs would be cationic because the outer mitochondrial membrane is relatively negatively charged, favoring electrostatics for membrane partitioning^4,54,55^. Furthermore, N-terminal AHs that contain both hydrophobic and positively charged amino acid residues meet the requirements for mitochondrial import^56–59^. As expected, the amino acid sequences of mito-only AHs trended towards a greater net charge compared to AHs that showed association with ER/Golgi membranes (Fig. 2a). There were no statistically significant differences in length, hydrophobic moment,, hydrophobicity, or % of hydrophobic residues (hydrophobic density) that clearly discriminated these categories (Figs. 2a and S9C).

We calculated hydrophilic and hydrophobic faces for the 33 AHs to determine how their amino acid compositions corresponded to their localization categories (Fig. 2b). AHs that showed association with ER/Golgi membranes were enriched in polar amino acid residues on their hydrophilic face compared to mito-only AHs, which contained more cationic amino acid residues (Fig. 2c). We used an unbiased statistical technique called Linear Discriminant Analysis (LDA) to find the linear combinations of features that best separate the two categories. The calculated amino acid residue frequencies on the hydrophilic or hydrophobic face for each AH was used as input variables (Fig. 2b). LDA showed the frequency of Ser/Thr and Arg/Lys amino acid residues on the hydrophilic face were most influential in distinguishing the different categories (Fig. 2d). In line with this, the calculated Ser/Thr to Arg/Lys (S/T:R/K) ratio of amino acids on the hydrophilic face of AHs that exclusively localized to mitochondria (mito-only) was significantly lower than that of AHs that displayed ER/Golgi localization (Fig. 2e).

We examined the net charge and hydrophobic density for AHs and found that the majority of mito-only AHs (11/19) had a high net charge (≥4, 7/19 AHs; Supplementary Data 15, columns V-AC) and/or a relatively high hydrophobic density (≥56%, 5/19 AHs; Supplementary Data 15). None of the AHs that exclusively localized to ER/Golgi membranes fulfilled these criteria (8/8 AHs; Supplementary Data 15). Instead, these AHs had relatively high S/T:R/K ratios on their hydrophilic face (2 ≤ S/T:R/K < 3, 5/8 AHs; Supplementary Data 15) as compared to all other AHs (S/T:R/K ≤ 1 for the other 24/25 AHs; Supplementary Data 15). The remaining 14 AHs did not fulfill any of these criteria and were not selective for ER/Golgi-membranes (mito-only localized, 8/14 AHs; mitochondria and ER/Golgi-localized, 6/14 AHs; Supplementary Data 15).

Thus, by screening the organelle localizations of a large number of AHs, we found that localization to ER/Golgi-membranes corresponded to low net charge and low hydrophobic density with a relatively high S/T:R/K ratio on the hydrophilic face. These properties are representative of their low ratio of positively charged to hydrophobic residues (0.08–0.25 for 6/8 AHs compared to 0.25–1.0 for 17/19 mito-only AHs; Supplementary Data 15, column AC), which indicates a greater contribution from the hydrophobic effect than electrostatics for membrane partitioning and aligns with binding the relatively neutral and loosely packed membranes of the ER/Golgi.

### Altering membrane binding preferences for a mito-only AH

**Fig. 3:**
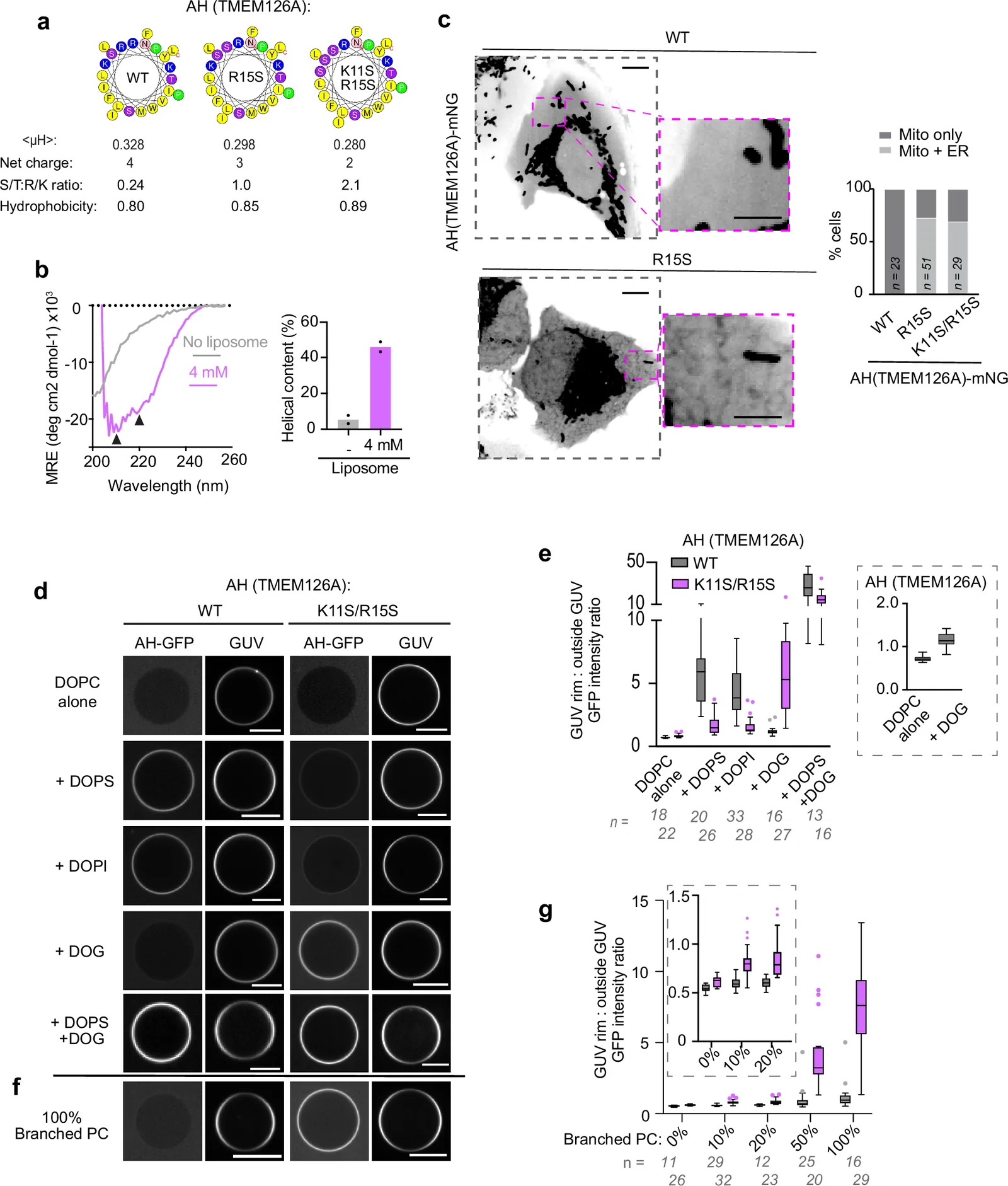
Mutagenesis of AH(TMEM126A) changes its  organelle localization and membrane binding preferences. **a** Helical wheel projections of wild-type (WT) and mutated AH(TMEM126A) with hydrophobic moment <µH > , net charge z, S/T:R/K ratio on hydrophilic face, and hydrophobicity shown. **b** Representative plot of circular dichroism spectral analysis of synthetic peptides as indicated incubated with and without 4 mM liposomes (10% DOPS + 12% DOG). Arrowheads indicate the local minima at 208 and 222 nm. Helical content estimate from MRE at 222 nm for *n* = 2 independent experiments. See Fig. S9D for the fitted curve and secondary structure estimate by BeStSel. **c** Representative spinning disk confocal images pseudocolored by inverting grayscale with accompanying magnified images. Plot of percent cells displaying indicated localizations expressing WT and mutated AH constructs. *n* =  number of cells. Scale bars, 10 µm or 5 µm (zoom). **d**, **f** Spinning disk confocal images of a single z-slice of GUVs incubated with the indicated AH-GFP recombinant protein. Box plots represent the GUV rim  association of AH(TMEM126A)-GFP (see “Methods” and Fig. S10B). Magnification of specified plot regions shown in (**e**, **g**). *n* = number of independent GUVs. Data points represent independent GUVs or cells pooled from two or three independent experiments. Box plots follow the Tukey method (center line: median; box: 25–75th percentiles; whiskers: 1.5× IQR; points: outliers). GUV giant unilamellar vesicle, DOPC 1,2-Dioleoyl-sn-glycero-3-phosphocholine, DOPS 1,2-dioleoyl-sn-glycero-3-phosphoserine, DOG 1,2-dioleoyl-sn-glycerol, DOPI 1,2-dioleoyl-sn-glycero-3-phospho-(1’-myo-inositol). Source data are provided as a Source Data file.

We used mutational analysis to interrogate how polar versus positively charged residues on the hydrophilic face of an AH directly impact membrane binding preferences. We chose a mito-only localized AH of the protein TMEM126A, which is defined as a multi-spanning inner mitochondrial protein in UniProt, but was not eliminated from our list because it passed filtering tests (see Figs. 1a and S1A). The features of AH(TMEM126A) include a high hydrophobic density (65%) and hydrophobicity (0.80), high net charge (*z *= 4), low S/T:R/K ratio on the hydrophilic face (0.24), and relatively high ratio of charged to hydrophobic amino acid residues (0.27). These parameters met the requirements for mito-only targeting based on our analysis above. Using mutagenesis, we tested if substituting a single or two charged residues with polar residues on the hydrophilic face (Fig. 3a) would alter organelle localization and binding to giant unilamellar vesicles (GUVs) with defined lipid compositions in vitro.

First, we demonstrated by circular dichroism (CD) analysis that AH(TMEM126A) folds into an α-helical structure in the presence of liposomes containing both conical and negatively charged lipids, as suggested by the local minima at around 208 and 222 nm (Fig. 3b; see Fig. S9D for secondary structure estimate by BeStSel^60^). Thus, AH(TMEM126A) is a membrane-binding helix, yet its properties are not favorable for binding to ER/Golgi membranes in cells. Note that the proline residue may introduce a kink that lowers helicity^61^.

Next, we introduced point mutations to increase the S/T:R/K ratio of AH(TMEM126A) from 0.24 to 1.0 to 2.1, which incrementally decreased the ratio of positively charged to hydrophobic residues, lowered the net charge, and increased hydrophobicity of each helix (Fig. 3a). These changes resulted in a shift from the exclusively mitochondrial localization of mNG-tagged AH(TMEM126A) to some localization to ER membranes (Fig. 3c).

We used low-curvature GUVs with defined lipid compositions to test if the substitutions in AH(TMEM126A) altered its membrane binding preference, and thus accounted for the changes in its organelle localization. The wild-type (WT) and mutated AH (K11S/R15S) fused to monomeric GFP (Fig. S9E) were purified from *E. coli* and incubated with GUVs generated with different lipid compositions. Neither AH(TMEM126A) peptide bound to DOPC-only GUVs, consistent with the absence of electrostatic interactions and lipid-packing defects. Both peptides bound strongly to GUVs containing negatively charged lipids (DOPS) and conical lipids (dioleoyl glycerol, DOG, a DAG analog known to induce packing defects in model membranes)^43,62^ (Fig. 3d, e). Only the WT peptide bound strongly to GUVs that contained negatively charged lipids (either +DOPS or +DOPI) and no DOG (Fig. 3d, e). These data suggested that AH(TMEM126A)^K11S/R15S^ requires acyl chain exposure from lipid packing defects to associate with membranes^10,43,63,64^. On the other hand, WT AH(TMEM126A) binds membranes primarily through electrostatic interactions, and this binding is enhanced by the presence of exposed acyl chains from packing defects. The propensity to fold into an α-helix can affect the affinity of a peptide to membranes^65^; however, a decreased propensity to fold is unlikely for the AH(TMEM126A)^K11S/R15S^ peptide because the mutations altered organelle and lipid composition preferences, rather than the overall degree of membrane association (see Fig. 3e).

We next tested the ability of each AH(TMEM126A) to bind to neutral membranes with packing defects. When the requirement for electrostatic interactions is reduced by a lack of anionic lipids in the membrane, the hydrophobic component drives membrane binding^4,8^. The mutated AH(TMEM126A)^K11S/R15S^ bound strongly to neutral membranes with DOG, demonstrating that the hydrophobic effect is the driving force for membrane binding under this condition (Fig. 3d, e). The WT AH(TMEM126A), however, was much less sensitive to packing defects in neutral membranes (Fig. 3d, e), suggesting that electrostatic interactions are required for its membrane binding even when there is a lack of anionic lipids in loosely packed membranes. This result combined with the highly enhanced binding of WT AH(TMEM126A) in the presence of GUVs containing both DOG and anionic lipids (Fig. 3d, e) suggested that this AH only recognizes packing defects in negatively charged membranes, a property previously described for the AH in α-synuclein^8,9^.

As a complementary way to test AH(TMEM126A) binding to accessible acyl chains, we generated GUVs with the lipid diphytanoyl phosphocholine (DPhPC), a branched-chain PC made up of fully saturated, methyl-branched chains that induce shallow packing defects^63,64^ (Fig. 3f, g). AH(TMEM126A)^K11S/R15S^ bound to GUVs in a manner that increased with greater percentages of DPhPC incorporated, while WT AH(TMEM126A) bound weakly even to GUVs made up of 100% DPhPC (Fig. 3g). This result further indicated that AH(TMEM126A)^K11S/R15S^ is sensitive to the presence of lipid packing defects, whereas WT AH(TMEM126A) requires electrostatic interactions for membrane binding.

In conclusion, substituting positively charged residues for polar residues on the hydrophilic face of AH(TMEM126A) increased its sensitivity to lipid packing defects in vitro, offering a mechanistic basis for its shift in localization from exclusively mitochondrial to include some binding to loosely packed ER membranes in cells. Defining the distinct membrane-binding properties of AH(TMEM126A) and its mutant forms provides a framework for interpreting the features that promote its association with the INM when targeted to the nucleus (see below).

### Association of AHs with the INM

**Fig. 4:**
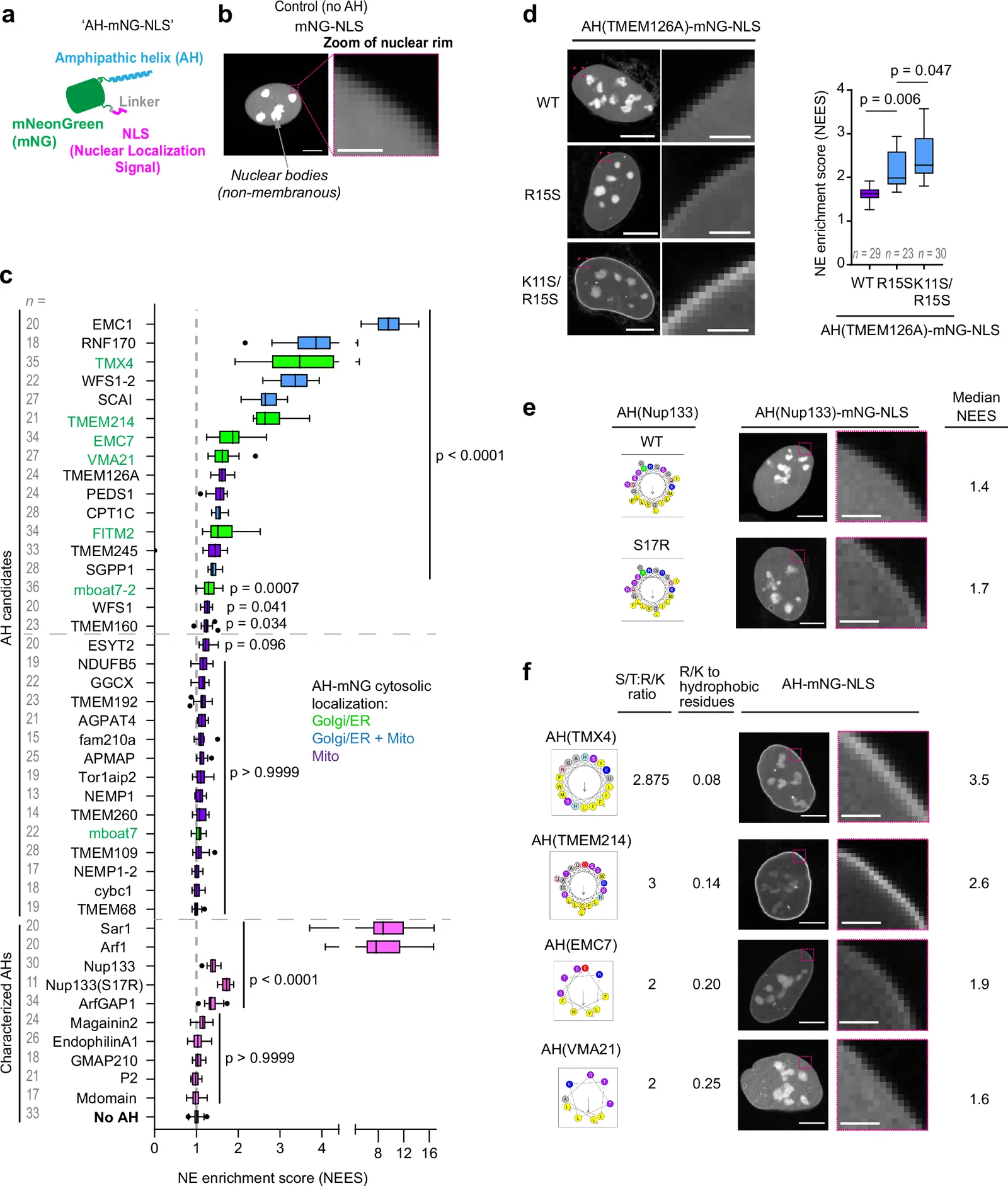
Nuclear targeting and INM enrichment of candidate and characterized AHs. **a** Schematic of AH-mNG and AH-mNG-NLS constructs. **b** Representative spinning disk confocal images of U2OS cells expressing mNG-NLS alone. Image is representative of ≥ 2 independent experiments with similar results. **c** Box plot of NE enrichment scores (NEES) normalized to mNG-NLS (no AH) control. Colors indicate localization when expressed in the cytosol, except for characterized AHs (pink) in which cytosolic localization was not assessed in this study. *n* =  number of nuclei (individual cells analyzed). Data represent individual cells pooled from two independent biological replicates. *P* values, Kruskal–Wallis test followed by Dunn’s multiple comparison test (two-sided). **d** Images and quantification of wild-type and mutant AH(TMEM126A)-mNG-NLS constructs. *n* = number of nuclei (individual cells). Data pooled from two independent biological replicates. *P* values, one-way ANOVA with Tukey’s multiple comparison test (two-sided). **e**, **f** Representative images of AH-mNG-NLS constructs with corresponding helical wheel projections. Images are representative of two independent experiments with similar results. Scale bars indicated. Box plots follow the Tukey method (center line: median; box: 25–75th percentiles; whiskers: 1.5× IQR; points: outliers). Source data are provided.

We appended a nuclear localization signal (NLS) to the C-terminus of mNG for the 32 AHs that localized to organelle membranes (see Fig. 1b and Supplementary Data 15; note that AH(KDSR) was the only AH excluded because of technical difficulties) as well as to an additional nine highly characterized AHs with established membrane binding properties (Supplementary Data 16). We analyzed INM enrichment for each nuclear-targeted AH candidate and established AH to infer features important for INM binding (Fig. 4a–c). We developed an image analysis pipeline to calculate the nuclear rim fluorescence signal intensity for each AH-mNG-NLS construct expressed in living U2OS cells (Fig. S10A). The median nuclear rim fluorescence intensity normalized to that of mNG-NLS control, termed the NE enrichment score (NEES), was used for all our analyses. Note that our image analysis pipeline eliminated the signal from nuclear bodies seen with the mNG-NLS control and AH-mNG-NLS constructs (Figs. 4b and S10a).

A general trend that emerged was that mito-only localized AHs had the lowest NEES, and in a majority of cases (14/19) the NEES was not statistically significant relative to control mNG-NLS signal (Fig. 4c and Supplementary Data 16; NEES median range 0.99–1.22). In contrast, almost all AHs that showed ER/Golgi-localization when in the cytosol (12/13 AHs) had statistically significant NEES relative to the mNG-NLS control (Fig. 4b, c and Supplementary Data 16; NEES median range >1.24). Furthermore, the mutated AH(TMEM126A) R15S and K11S/R15S that partially localized to the ER, and bound neutral membranes with packing defects in vitro, displayed higher NEES than the cationic WT AH(TMEM126A) that required electrostatics for membrane binding (Fig. 4d and Supplementary Data 16, see also Fig. 3d, e). These data support the interpretation that AHs associating with the INM share features with ER/Golgi-localized, rather than mitochondrial, AHs, and that electrostatic interactions are not the dominant contributor to their INM association.

We next determined the NEES scores of nuclear-targeted, established AHs with known membrane binding properties. We chose nine AHs with well-defined membrane preferences and distinct mechanisms for membrane binding and compared the degree of INM binding to AH candidates.

The AH of Sar1 that binds to ER/Golgi membranes in the cytosol^4,13^, was significantly enriched at the INM when directed to the nucleus (Fig. 4c, NEES median >7.6). The same was true for AH(Arf1), but it is likely lipid-modified^4^ and thus we eliminated it from further analysis. Only one candidate AH(EMC1) enriched to a similarly high level as AH(Sar1) (NEES median > 9; all other AH candidates NEES median ≤3.86). Both AH(Sar1) and AH(EMC1) have few positively charged residues relative to hydrophobic residues (0.18 and 0.23 R/K:hydrophobic residues, respectively) with high hydrophobicity (0.71 and 0.81, respectively) and a normal density of hydrophobic residues (48% and 54%, respectively), suggesting that their strong INM enrichment is mainly through the hydrophobic effect over electrostatic interactions.

The characterized AHs with NEES indistinguishable from control mNG-NLS all were highly charged (Fig. 4c, medians of NEES ~ 1.0). These were the negatively charged M and P2 domain of CCTa (net charge −5), the AH of Endophilin A1 that is rich in basic residues with high net charge (net charge 4), or Magainin2 that is rich with Lysine residues on the hydrophilic face and known to prefer highly negatively charged membranes (Supplementary Data 16)^4,66–68^. These data further supported that electrostatics is not the major contributor to INM association for a subset of AHs in this study, consistent with a relatively neutral membrane surface charge.

We determined INM binding of nuclear-targeted curvature-sensitive ALPS motifs (AH(Nup133) and ALPS1-ALPS2 of ArfGAP1^69^; Fig. 4c) to test if minimizing the electrostatic component with a relatively low hydrophobic density, such that a high density of lipid packing defects is required for membrane binding, would allow association with the INM^8^. Note that the ALPS motif for GMAP210 fused to the N-terminus of mNeonGreen (mNG) did not show any NE enrichment (Fig. 4c, median NEES 1.0). This is likely because of its low avidity for membranes in this arrangement, as suggested by prior reports^13^. The low, albeit significant, NEES of the ALPS motifs (Fig. 4c, median NEESs 1.38 and 1.34, respectively, for AH(Nup133) and AH(ArfGAP1)) suggested that the relative density of packing defects in the flat INM is too low for strong binding by curvature-sensitive AHs. Mutating a single serine residue near the center of the polar face of AH(Nup133)-mNG-NLS to a positively charged arginine residue increased INM binding (NEES 1.72; Fig. 4c, e). Thus, adding a charge to decrease the curvature sensitivity of AH(Nup133)^13^, increased binding to the flat INM, consistent with the possibility that the INM has a relatively low-charge surface that can concentrate weak AHs and facilitate subsequent membrane binding.

The observation that ALPS motifs are not the highest INM binders aligns with the NEES for AH candidates with the greatest S/T:R/K ratios (S/T:R/K ratio >2) (Fig. 4c), which were exclusively ER/Golgi-bound when in the cytosol (Fig. 2). The degree of INM binding for these AHs was significantly greater than control and mito-only AHs (6/7 AHs, Fig. 4c), but generally lower than AHs from the ER/Golgi + mito category (Fig. 4c). A greater S/T:R/K ratio is thus not a reliable indicator for the degree of INM binding, consistent with our findings that high S/T:R/K ratios of ALPS motifs does not result in strong INM binding (Fig. 4c and Supplementary Data 16). Instead, the ratio of charged to hydrophobic residues, with a lower ratio displaying higher binding, corresponded with the NEES for these AHs (Fig. 4f and Supplementary Data 16), which fits with a greater reliance on the hydrophobic effect than electrostatics for INM binding. Interestingly, some of these candidate AHs also contained a relatively low density of hydrophobic residues (e.g., AH(TMEM214), hydrophobic density 35% compared to AH(Sar1), hydrophobic density 47.8%) (Supplementary Data 16). Helices containing a weak charge and low hydrophobic density need access to exposed acyl chains via packing defects as a prerequisite for membrane binding^62,70^. This requirement supports our conclusion that binding of these AHs to the INM is through detection of lipid packing defects, in line with their exclusive cytosolic ER/Golgi-localization when expressed in the cytosol.

**Fig. 5:**
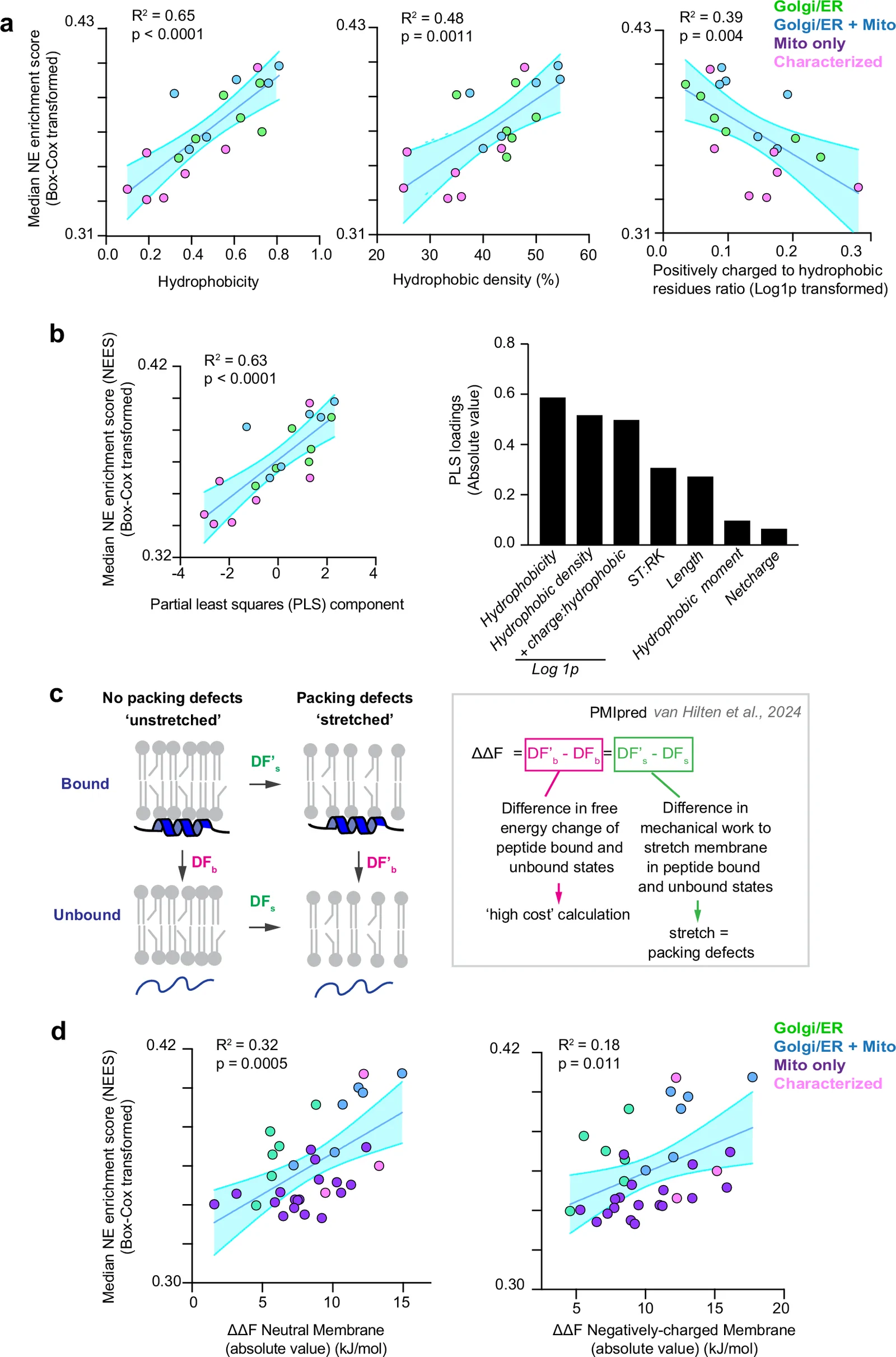
Correlation analyses of nuclear envelope enrichment scores (NEES) and amphipathic helix (AH) properties. **a** Linear regression between median NEES (Box–Cox transformed) and indicated AH properties. *n* = number of independent AH sequences. Lines represent linear regression; shaded areas indicate 95% confidence intervals. *P* values and *R*² were obtained by linear regression (two-sided). Exact *P* values are provided in Source Data. Established AHs and candidate AHs with ER/Golgi-localization when in the cytosol are included (see Fig. 4c). **b** Same as (**a**) with PLS component. The bar graph shows PLS loadings of the AH parameters. **c** Schematic of *ΔΔF* calculation. **d** Linear regression between *ΔΔF* and NEES for all AHs ≤24 amino acids in length (also see Supplementary Data 17). *n* = number of AH sequences. Lines represent linear regression; shaded areas indicate 95% confidence intervals. *P* values and *R*² were obtained by linear regression (two-sided). Source data are provided.

We reasoned that the NEES likely depends on a combination of parameters, given that both highly and weakly hydrophobic helices bind to the INM and that comparable hydrophobicity or hydrophobic densities displayed different NEES values (Supplementary Data 16). Pairwise correlation analysis of 38 nuclear-targeted AHs (31 candidate AHs and seven characterized AHs, Fig. 4c) did not show a strong relationship between the NEES and any of the descriptors (Fig. S11A–D). When we eliminated mito-only AHs, the majority of which did not bind the INM (Fig. 4c), pairwise analysis revealed a positive correlation between the NEES of the remaining 19 AHs and hydrophobicity (Fig. 5a,  *R*^2^= 0.63; *P* < 0.0001). There was a statistically significant, although weaker, positive correlation with hydrophobic density (*R*^2^ = 0.48) and a weak negative correlation to the ratio of positively charged to hydrophobic residues (*R*^2^ = 0.39) (Fig. 5a). These data suggest that a single parameter alone does not fully account for the NEES variation and thus the degree of AH association to the INM.

To identify if a combination of analyzed properties covary with the NEES, we applied Partial Least Squares (PLS) analysis (Fig. 5b). A dominant multivariate trend emerged from PLS analysis, characterized primarily by hydrophobicity, hydrophobic density, and the relative contribution of basic residues versus hydrophobic amino acids (PLS component to NEES *R*² =  0.63, Fig. 5b). Other parameters (e.g., helix length) contributed minimally to this component. Note that we used this analysis descriptively, to summarize potential covariance rather than a predictive model. The PLS analysis suggests that a combination of these parameters contribute to AH binding to the INM, providing an explanation for why AHs with high hydrophobicity can bind effectively to the INM even in the presence of charged amino acids on the hydrophilic face, while relatively low hydrophobic density and low charge can also be permissive for INM binding. Together, correlation analyses support the interpretation that the hydrophobic effect contributes more strongly than electrostatics to INM association, leading us to infer that the INM contains lipid packing defects with a low surface charge. Note that, given the *R*^2^ value of 0.63, other descriptors not accounted for also contribute to the NEES.

We also used PMIpred, a computational predictor based on a transformer neural network trained on data derived from molecular dynamics (MD) simulations^71^, to provide further support that AH association with the INM involves detection of packing defects. PMIpred calculates the difference in free energy reduction upon AH helix association to stretched and non-stretched membrane bilayers containing a constant number of lipids (*ΔΔF*) based solely on peptide sequence (Fig. 5c)^71–73^. Since stretched membranes result in the exposure of acyl chains to the solvent and to the increase of packing defects^39,41^, high *ΔΔF* values indicate that the hydrophobic effect is a major driving force for membrane insertion. PMIpred calculates *ΔΔF* values either with PC-only (neutral) or PC + 20% PG (negatively charged) membranes. NE-enrichment scores (for AHs <24 aa in length; Supplementary Data 17) were significantly correlated with *ΔΔF* values for neutral and negatively charged membranes (Fig. 5d). While the moderate strength of the correlations implicates mechanisms of INM binding beyond the hydrophobic effect and packing defect detection, the *ΔΔF* values overall support our conclusion that AH binding to the INM can be favored by the presence of lipid packing defects.

### INM-enrichment of AHs upon nuclear swelling versus stretch

We hypothesized that for AHs sensitive to lipid packing defects at the INM, there should be an increase in their binding in response to membrane tension^39,41^. If the increased density of packing defects is high enough, it should also promote partitioning of ALPS motifs to the flat membrane^43^. We used cell-substrate stretch and hypotonic shock to test this idea, which also allowed us to compare how membrane tension resulting from distinct mechanical inputs might influence lipid packing.

**Fig. 6:**
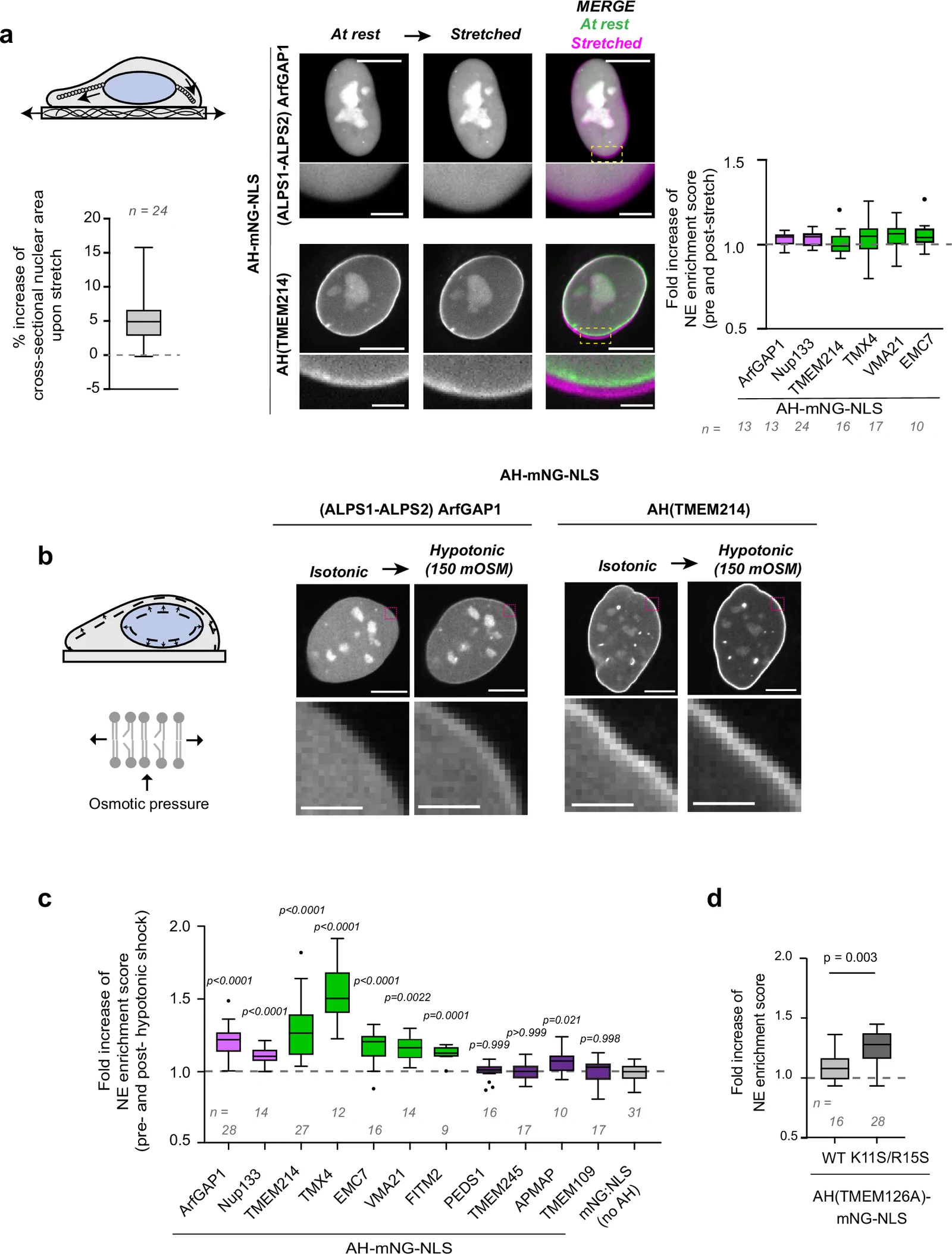
Response of nuclear-targeted amphipathic helices (AHs) to hypotonic shock and cell stretch. **a** Schematic representation of cell-substrate stretch from the cell exterior and corresponding nucleo-cytoskeletal linkages directly imposing local forces on the nuclear envelope (NE). Box plot of percentage increase in the cross-sectional nuclear area upon application of cell-substrate stretch. Spinning disk confocal images of a single z-section of living HeLa cells expressing the indicated AH-mNG-NLS construct before (at rest) and during cell-substrate stretch (stretched). Overlay of pseudocolored images of nuclei before (green) or during (magenta) cell-substrate stretch is shown. Box plot of fold increase in NE enrichment scores after cell-substrate stretch. *n* = number of nuclei (individual cells). Data pooled from two to three independent biological replicates. **b** Representative images with a magnified region of living U2OS cells with indicated AH-mNG-NLS before and after hypotonic shock. **c**, **d** Box plot of fold change in NE enrichment after hypotonic shock with indicated AH constructs normalized to the no AH control. *n* = number of nuclei (individual cells analyzed). Data pooled from two independent biological replicates. *P* values, Brown–Forsythe and Welch ANOVA followed by Dunn’s multiple comparison test (two-sided) in (**c**), and unpaired two-sided *t* test in (**d**). Exact *P* values are provided in Source Data. Box plots follow the Tukey method (center line: median; box: 25–75th percentiles; whiskers: 1.5× IQR; points: outliers). Scale bars, 10 µm or 2 µm (magnified images). Source data are provided.

We used a custom membrane stretch device to impose equibiaxial stretch on living HeLa cells^74^. In short, this device induces cell-stretch that transmits to cytoskeletal forces imposed directly on the NE to deform the nucleus^74^. The maximal cell stretch that could be applied that deforms the nucleus but doesn’t cause plasma membrane rupture (Fig. 6a) did not promote INM-association of ALPS motifs of ArfGAP1 and Nup133 (Figs. 6a and S12A). Candidate AHs predicted to sense lipid packing defects also did not enrich at the INM under cell stretch conditions (Figs. 6a and S12A). These results suggested that INM forces transmitted from cell-substrate stretch may not induce sufficient membrane tension to increase the density of packing defects.

We then tested how AHs respond to membrane tension induced by nuclear swelling^33,36^. The ALPS motifs of ArfGAP1 and Nup133 displayed significantly increased binding with the INM in U2OS cells treated with mild hypotonic shock (~150 mOSM) (Fig. 6b, c), which does not induce organellar vesiculation^42,75^. A similar response was observed for candidate AHs with high S/T:R/K ratios as well as the K11S/R15S mutant AH(TMEM126A), but not for the mito-only AHs (Figs. 6c, d and S12B). AH(TMEM214) behaved similarly in multiple cell types (Figs. 6c and S12C), suggesting that its INM enrichment in response to nuclear swelling is not cell type specific.

Together, the fact that candidate AHs with high S/T:R/K ratios and ALPS motifs increase in enrichment at the INM in response to hypotonic shock further substantiates our hypothesis that these AHs bind lipid packing defects. Additionally, these results suggest that different forces should be treated as highly distinct in their consequences on INM lipid packing, with implications for the response of the nucleus to specific mechanical inputs.

### Structural analysis of AH(TMEM214)

**Fig. 7:**
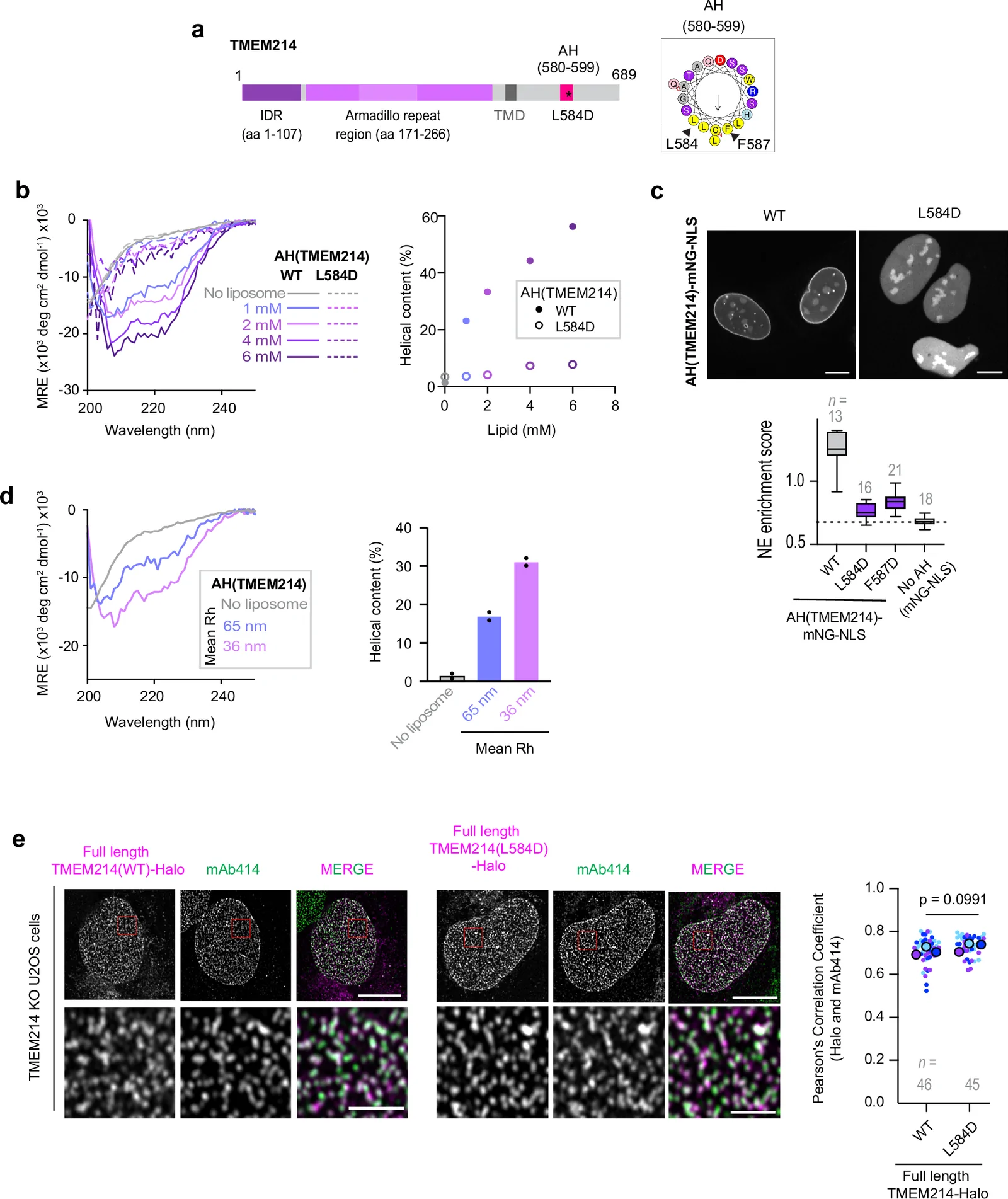
AH(TMEM214) folds upon membrane binding, and full-length TMEM214 localizes to nuclear pore complexes. **a** Schematic of domain architecture of TMEM214. Helical wheel diagram of AH(TMEM214). Arrowheads mark amino acid residues mutated in subsequent panels. **b** CD spectra of indicated peptides with increasing liposome concentration (+10% DOPS + 12% DOG). Plot of helical content estimated from MRE at 222 nm (right). Mean of *n* = 2 independent experiments shown. See also Fig. S13. **c** Representative spinning disk confocal images of a single section in living U2OS cells with indicated constructs. Box plots of NE enrichment score are shown by the Tukey method (the middle line: median; box: 25th and 75th percentiles; whiskers: the largest/smallest values or 25th/75th percentiles −/+ 1.5IQR; dots: outliers). *n* = number of nuclei. **d** CD spectra with liposomes of varying size. 4 mM liposomes (+10% DOPS) were extruded with different hydrodynamic radii, as indicated. Plot of helical content estimated from MRE at 222 nm shown. Mean of *n* = 2 independent experiments. See also Fig. S13. **e** Airyscan single-section images of fixed TMEM214 KO cells expressing indicated full-length TMEM214-Halo and immunostained for nuclear pore complexes. Plot of Pearson correlation coefficients shown. *n*  = number of cells; *N* = 3 independent biological replicates. *P* value, paired two-sided *t* test. Exact *P* values are provided in Source Data. IDR Intrinsically disordered region, TMD transmembrane domain, Rh hydrodynamic radius. Source data are provided as a Source Data file.

We performed structural analysis of AH(TMEM214), which enriches at the flat INM when nuclear targeted (Fig. 4c, f), to determine if our screening approach successfully identified an AH (1) in a previously uncharacterized NE-associated protein that (2) prefers lipid packing defects. Amongst the other proteins with AHs that met the criteria of binding the INM (see Fig. 4), TMEM214 (Fig. 7a; see Fig. S13A for AlphaFold predicted structure) was intriguing because it is an uncharacterized protein with evidence for INM localization in multiple cell types^46–48^ and has been uncovered as an interactor of nuclear pore complex proteins by proteomics^76^.

Circular dichorism (CD) spectra analysis showed that WT AH(TMEM214) is α-helical in structure in the presence of liposomes containing negatively charged lipids and the conical lipid DOG, and this was dependent on liposome concentration (Figs. 7b and S13B, C). The amphipathic nature of AH(TMEM214) was required because introducing a charged amino acid residue in the apolar face abolished α-helix formation (AH(TMEM214)-L584D; Figs. 7b and S13C, D). Importantly, this mutation prevented localization of AH(TMEM214)-L584D with the INM (Fig. 7c). These data suggest that AH(TMEM214) folds into a helix upon binding to the INM.

Curved membranes contain a high density of lipid packing defects that increase in size and density with increasing membrane curvature^10–12^. If AH(TMEM214) binds lipid packing defects, then there should be an increase in helicity in the presence of increasing membrane curvature^8,11,12^. AH(TMEM214) incubated with vesicles without DOG but with defined and uniform hydrodynamic radii of increasing curvature (mean Rh 65 nm or 36 nm) showed binding in a curvature-dependent manner accompanied by an increase in helicity (Figs. 7d and S13E). These data, combined with the fact that the AH(TMEM214) binds the flat INM, provide further support for lipid packing defect detection at the INM.

Using CRISPR/Cas9 gene edited U2OS cells deleted of TMEM214, we determined the localization of exogenously expressed full-length TMEM214 (Fig. S14A, B). Expression of TMEM214-mNG in U2OS cells carrying endogenous TMEM214 resulted in highly aberrant localization even at moderate levels of expression (Fig. S14C). In contrast, low-level expression of TMEM214-mNG in the TMEM214-knockout background showed a punctate nuclear rim pattern (Fig. S14D), which was co-localized to NPCs as shown by super-resolution imaging (Fig. 7e). The L584D mutation introduced in full-length TMEM214 did not impact its NPC localization (Fig. 7d), which may be expected if TMEM214 contains other regions that interact with NPC components. The fact that AH(TMEM214) independent of its protein backbone binds strongly to the flat INM suggests that the population of packing defects at the INM is sufficient for favorable binding by this AH^4,8,11,43^. Furthermore, this AH in isolation may not have a high degree of curvature sensitivity like ALPS motifs because the TMD region of the protein backbone is already embedded in the target membrane (see Fig. S15 for a schematic), although its curvature binding within the full-length protein remains to be tested. Thus, TMEM214 is a nuclear pore-localized transmembrane containing protein with an AH that binds lipid packing defects, which are intrinsic to the curved nuclear pore membrane characterized by both negative and positive curvature on the nucleoplasmic/cytoplasmic and luminal facing regions (Fig. S15).

## Discussion

Mechanistic studies suggest that AHs bind membranes in three steps^4,7,62,68,70,77–79^. First, the peptide sequence accumulates near membranes. Then, it is transferred to the membrane driven by the hydrophobic effect. Finally, a transition from random coil to α-helix occurs, which significantly contributes to the free energy of binding. Collectively, we provide evidence for select AHs that bind the INM through this mechanism, suggesting features of the INM permissive for such interactions. We further provide a rich resource of previously uncharacterized AHs in transmembrane containing proteins.

Through screening >60 AHs, we uncover AH candidates that associate with the INM mainly through the hydrophobic effect with a minor electrostatic contribution—these AHs have amino acid properties that fit with binding to membranes with acyl chain accessibility through lipid packing defects. Our mutagenesis experiments on AH(TMEM126A) as well as our computational and correlation analyses from image-based screening of candidate and well-characterized AHs further support our model that sensitivity to lipid packing defects with a minor contribution from electrostatics promotes INM association. Structural analysis of AH(TMEM214), and evidence for NPC-association of full-length TMEM214, provides independent support for AHs favoring bilayer phospholipid accessibility at the INM and shows that our screening approach uncovered an AH in a NE-associated protein. Future work is required to determine if even cationic AHs uncovered in our study may be relevant to INM binding in specific contexts^30,80–82^; however, some caution should be taken as some may not be part of a protein resident to the NE given our filtering criteria or may not be membrane-binding AHs because they are predicted to be part of a structured domain.

Prior work using nuclear lipid biosensors showed that the conical lipid DAG^31^ and the negatively charged lipid PS^42^ are present at the INM; however, the relative abundances of these lipids and thus the impact on the overall properties of the INM were not clear. Our study analyzes features of AH association with the INM, suggesting lipid packing defects and a low surface charge at the INM, compatible with the presence of DAG and anionic PS lipid. MD simulations have shown that the size and density of packing defects promoted by DAG in flat membranes resemble those in positively curved membranes^43^, further supporting our model that lipid packing defects are a property of the INM, the density of which is lower than that of convex membranes that favor partitioning of weak ALPS motifs.

We used nuclear-targeted ALPS motifs and candidate AHs that bind loosely packed membranes to show that an increase in INM lipid packing defects results from nuclear swelling, but not whole cell stretch. It is possible that in response to cell stretch, which induces tension on the NE on relatively longer time scales, membrane flow from the ER^34^ or nuclear invaginations or even a lipid metabolism program is induced limiting lipid packing defects. In light of this, we can not exclude the possibility that a metabolic program in response to nuclear swelling induces more lipid packing defects (e.g., hydrolysis of lipid chains resulting in more lysophospholipids). Cell stretch may also only result in local membrane tension or not high enough tension on the membrane, partly through dissipation by NPCs and the nuclear lamina. Thus, different mechanical forces imposed on the NE have distinct effects on lipid packing, with expected differences in the response of lipid-protein interactions and thus activation of downstream processes.

Evidence for arachidonic acid attached to INM phospholipids^32,33^ as well as PUFA-rich DAG at the INM^31^ suggests that the INM may be made up of shallow packing defects. The depth of lipid packing defects depends on the composition of fatty acyl chains attached to phospholipids and can impact AH binding^63,83,84^. Shallow lipid packing defects occur when acyl chain exposure is closer to lipid headgroups induced by polyunsaturated fatty acids^63,83^. Our data showing that the mutated AH(TMEM126A)^KR/SS^ associated with the INM and bound to GUVs containing branched chain PC, which is known to induce shallow packing defects in membranes^63^, supports the possibility of shallow lipid packing defects at the INM. It is interesting to note that there is a Lysine residue at the polar–apolar boundary of WT AH(TMEM126A), which contributes both to electrostatics through the positively charged amino group and hydrophobicity through the carbon chain, and may be less adapted to binding shallow lipid packing defects at the INM. Lysine at this position prefers deep packing defects through snorkeling in the lipid bilayer such that the charged amino group positions at the charged membrane surface while the hydrocarbon part of the side chain embeds in the hydrophobic part of the membrane^4,8,85,86^. Future work using 3D modeling of AHs in the membrane and calculations of accessible hydrophobic and polar areas in lipid compositions that mimic the INM, as well as calculations of interaction energy with lipids, may be needed to fully understand the cellular data.

Among the candidate AHs we uncovered, we identified an AH in TMEM214 that binds to lipid packing defects and showed that TMEM214 localizes to nuclear pore complexes. The predicted structure of TMEM214 shows that its nucleoplasmic domain is made up of stacked alpha helices (Fig. S13A) with sequence homology to Armadillo (ARM) repeat regions that exist in many signaling and structural adapter proteins, including the nuclear transport factor importin-alpha^87,88^. TMEM214 is predicted to be a single or dual pass transmembrane protein, depending on the prediction tool used, and in either case, this would place its AH(TMEM214) facing the lumen rather than the nucleoplasmic side of the membrane (Fig. S15). We cannot exclude the possibility that this region may have distinct membrane properties from chromatin-facing INM and that the AH in the context of the full-length protein prefers distinct membrane compositions, but it is intriguing that AH(TMEM214) binds the flat INM in isolation, as it suggests that the INM may have similar features to the curved nuclear pore membrane. The pore membrane contains both positive and negative curvature on the nucleoplasmic/cytoplasmic and luminal facing regions (Fig. S15). Thus, an AH facing the luminal face of the pore membrane might be required to help stabilize or to stably associate NPCs with the curved membrane, which unlike vesicles are stable for long time scales. We were unable to find homologs of TMEM214 in lower organisms. Future work is needed to determine its function in the structure and biogenesis of the pore membrane, and further characterize its AH preferences for specific membrane compositions.

Membrane packing defects may help accommodate INM remodeling, mechanics, and repair^17,89^. In addition, the lipid content of the INM can be altered by both local and global lipid metabolism impacting these properties and lipid-protein interactions^29,31,80,82,90–98^. Our work examining features correlated with INM-association of a subset of AHs, as well as mutagenesis and in vitro studies on AH(TMEM126A) and AH(TMEM214), suggests physicochemical features promoting amphipathic helix-INM interactions and provides a framework for understanding the properties of the INM in mammalian cells, an important area to consider in both healthy conditions and in diseases associated with the NE.

## Methods

**Table:** Reagents and resources

Reagent or resource	Source	Identifier
**Antibodies**		
Mouse anti-mNeonGreen [32F6]	ChromoTek	Cat# 32f6-100, RRID:AB_2827566
Mouse anti-α-tubulin [DM1a]	Millipore	Cat# 05-829, RRID:AB_310035
Rabbit polyclonal anti-TMEM214	Thermo Fisher Scientific	Cat#: PA5-98198, RRID:AB_2812811
Mouse anti-Nuclear Pore Complex Proteins [mAb414]	BioLegend	Cat# 902901, RRID:AB_2565026
**Bacterial and viral strains**		
Escherichia coli BL21(DE3) cells	New England Biolabs	Cat# C2527
**Chemicals, peptides, and recombinant proteins**		
18:1 (Δ9-Cis) PC (DOPC)	Avanti	Cat# 850375 C
18:1 DG (DOG)	Avanti	Cat# 800811 C
18:1 PS (DOPS)	Avanti	Cat# 840035 C
18:1 PI (DOPI)	Avanti	Cat# 850149 P
4ME 16:0 PC (DPhPC)	Avanti	Cat# 850356 C
Custom peptide (purity > 95%) AH of TMEM214 (WT): CASHLAWFGDSLTSLSQRLQ	LifeTein	N/A
Custom peptide (purity > 95%) AH of TMEM214 (L584D): CASHDAWFGDSLTSLSQRLQ	LifeTein	N/A
Custom peptide (purity > 95%) AH of TMEM126A (WT): NILSYWIRTSKPVFRKMLFPILL	LifeTein	N/A
Custom peptide (purity > 95%) AH of TMEM126A (K11S/R15S): NILSYWIRTSSPVFSKMLFPILL	LifeTein	N/A
JF552-HaloTag ligand	Luke Lavis lab at Janelia Research Campus^129^	N/A
**Experimental models: cell lines**		
U2OS	Frank Slack lab (Harvard Medical School)	N/A
U2OS TMEM214 KO	This study	N/A
HeLa	Single Molecule Biophotonics group at ICFO (The Institute of Photonic Sciences)	N/A
MEF	Nadya Dimitrova Lab (Yale University)	N/A
COS-7	Bewersdorf lab (Yale School of Medicine)	N/A
**Recombinant DNA**		
AH candidates-mNG (pCMVΔ5)	This study	N/A
AH candidates-mNG-NLS (pCMVΔ5)	This study	N/A
mNG-NLS (No AH) (pCMVΔ5)	This study	N/A
mNG alone (pmNG-N3)	This study	N/A
DsRed-KDEL	Bewersdorf lab (Yale School of Medicine)	N/A
pmScarlet-H_Giantin_C1	Dorus Gadella^130^	Addgene plasmid # 85049; RRID:Addgene_85049
mRuby-Mito-7	Michael Davidson	Addgene plasmid # 55874; RRID:Addgene_55874
AH(Nup133)-mNG-NLS (pCMVΔ5)	This study	N/A
ALPS1-ALPS2(ArfGAP1)-mNG-NLS (pCMVΔ5)	This study	N/A
ALPS (GMAP210)-mNG-NLS (pCMVΔ5)	This study	N/A
AH (Arf1)-mNG-NLS (pCMVΔ5)	This study	N/A
AH (Sar1)-mNG-NLS (pCMVΔ5)	This study	N/A
AH (EndophilinA1)-mNG-NLS (pCMVΔ5)	This study	N/A
AH (Magainin2)-mNG-NLS (pCMVΔ5)	This study	N/A
M domain (CCTa)-mNG-NLS (pCMVΔ5)	This study	N/A
P2 AH (CCTa)-mNG-NLS (pCMVΔ5)	This study	N/A
6xHis-SUMO-AH(TMEM126A) WT-AcGFP1 (pET SUMO)	This study	N/A
6xHis-SUMO-AH(TMEM126A) K11S/R15S-AcGFP1 (pET SUMO)	This study	N/A
TMEM214-HaloTag (pCMVΔ5) WT, L584D and L543E/L584D	This study	N/A
LongHelix (TMEM214)-mNG with/without NLS (pCMVΔ5)	This study	N/A
**Software and algorithms**		
FIJI v2.16	Ref. ^131^	https://imagej.net/Fiji
GraphPad Prism v9 and v10	GraphPad Software	https://www.graphpad.com/scientificsoftware/prism/
SnapGene v8.0	GSL Biotech LLC	https://www.snapgene.com/
TMHMM v2.0	Ref. ^50^	https://services.healthtech.dtu.dk/service.php?TMHMM-2.0
HeliQuest v2	Ref. ^51^	https://heliquest.ipmc.cnrs.fr/
BeStSel v2024	Refs. ^60,128^	https://bestsel.elte.hu/sexamin.php
Python version 3.8 or 3.11	Python Software Foundation	https://www.python.org/
Anaconda v2.6.3	Anaconda Inc	https://www.anaconda.com/products/distribution
AlphaFold Protein Structure Database model v4	Ref. ^53^	https://alphafold.ebi.ac.uk/
PAE viewer (no versions)	Ref. ^127^	https://pae-viewer.uni-goettingen.de/
PyMol v2.5.0	Schrödinger, LLC	https://www.pymol.org/
PMIpred v3.1	Ref. ^71^	https://pmipred.fkt.physik.tu-dortmund.de/curvature-sensing-peptide/

### Computational screening

Putative NE proteins were curated through integrative data mining using custom Python scripts^99^. Five published NE proteomics datasets^44–48^ were combined using UniProt identifiers as the primary key for human proteins. When UniProt IDs were not provided, NCBI accession numbers^44^ or gene names^45–47^ were converted using the UniProt Retrieve/ID Mapping tool or API. Obsolete or invalid entries were excluded. For mouse and rat datasets, human orthologues were identified via UniProt API queries using gene names (e.g., *ctdnep1*, *Homo sapiens*). This yielded an initial candidate NE protein list (~405 proteins). A subsequently published NE proteomics dataset^100^ was not included.

To provide independent support for nuclear localization, a separate reference set of nuclear proteins was obtained from the Human Protein Atlas (Supplementary Data 6 in ref. ^101^). Proteins were included if (i) the “IF location score” contained terms including “nucle” (e.g., NM, nucleoplasm, nucleoli) and (ii) “Reliability” was classified as “Supported” or “Validated,” yielding ~3000 nuclear proteins. This dataset was used only as an external reference and was not incorporated into the candidate NE protein list. A broad definition of nuclear localization was intentionally used to include all nuclear compartments, accommodating proteins with mixed localization (e.g., NE and nucleoplasm) and reflecting the design of the screening pipeline, which intentionally tolerates false positives to minimize loss of true NE proteins.

For secondary curation, UniProt subcellular localization annotations were retrieved via API for the candidate NE protein list (405 proteins after consolidation and removal of redundancies). Proteins were classified as NE- or ER-associated if they met all of the following criteria: (i) localization annotations included nuclear or ER-related terms (including NM, NE, nuclear lamina, nuclear pore complex, ER, ER membrane, ER–Golgi intermediate compartment, or ER lumen); (ii) annotations were supported by manually curated evidence codes (ECO:0000269, ECO:0000305, ECO:0000250, ECO:0000255, ECO:0000312, or ECO:0007744); and (iii) annotations were not solely derived from any of the five NE proteomics studies (PMIDs: 12958361, 20693407, 20876400, 22990521, 31142202), to avoid circularity. ER localization was included because NE proteins frequently exhibit ER pools, and some ER proteins access the INM, and UniProt annotations and proteomics datasets were treated as complementary sources.

Finally, proteins were classified as NE-associated if they met either of the following criteria: (i) presence in two or more NE proteomics datasets, or (ii) presence in a single dataset together with independent support from either Human Protein Atlas nuclear localization or UniProt-derived NE/ER localization.

#### Processing and integration of MemBrain predictions

MemBrain prediction results for 11,759 proteins across multiple organisms were downloaded from the MemBrain database^49^. Custom Python scripts were used for processing^102^. Protein/gene names and organism information were retrieved via the UniProt API, and lineage information was used to restrict the dataset to metazoans. Non-human proteins were mapped to human proteins by matching gene names, as described above for NE proteome datasets. The resulting MemBrain-derived protein list was merged with the curated NE protein list using UniProt IDs as the unique identifier.

Candidate AHs were assessed using a combination of computational and manual criteria. Sequences were analyzed using HeliQuest to evaluate amphipathicity, including visual inspection of hydrophilic and hydrophobic faces and calculation of the discrimination factor (D-factor), with a cutoff of >0.68^51^. This combined approach was used because D-factor alone can yield false positives, particularly for highly charged helices. Transmembrane helices were predicted using TMHMM^50^; candidates were excluded if more than 50% of the AH overlapped with a predicted transmembrane domain. Partial overlaps were retained, as functionally relevant AHs can partially coincide with transmembrane segments (e.g., IRE1)^16^.

### Plasmid construction

Gene insertion was performed using In-Fusion HD Cloning Plus or Snap Assembly (Takara), unless otherwise stated. Site-directed mutagenesis was carried out by whole-plasmid PCR followed by circularization. All constructs were verified by DNA sequencing. Where indicated, a bipartite nuclear localization signal (NLS) from Xenopus laevis nucleoplasmin (KRPAATKKAGQAKKKK)^103,104^ was used.

The mNeonGreen (mNG) construct was generated by replacing EGFP in pEGFP-N3 with mNG (gift from Hiroyuki Arai), expressed under the CMV promoter.

For AH screening, pooled oligonucleotides encoding candidate AH sequences were synthesized (Twist Biosciences), PCR-amplified, and inserted into a modified pCMVΔ5-Sun2-mNG backbone, in which the Sun2 coding sequence was removed^31,105^. Glycine linkers were incorporated to standardize oligonucleotide length. In short, sequences included AAACGGGCCCTCTAGAGCCACC*ATG*GGA (GGA)n XXX (GGT)n GGATCGAATTCTGCAGTCGACGGTAC, where XXX indicates each AH sequence, underlined sequences indicate the annealing sites for primers, *ATG* is the start codon, (GGA)n and (GGT)n are glycine linkers with n greater than or equal to zero and less than or equal to 7. The length of glycine linkers was chosen to keep the length variance of DNA oligos in the pool within +/− 10% of the median length for better efficiency of oligo pool synthesis, as instructed by Twist Biosciences. PCR was performed with the single-stranded oligo pool used as a template by forward and reverse primers (complementary to underlined sequences indicated above) with KAPA HiFi HotStart ReadyMix PCR Kit (50-196-5217; Roche Diagnostics) and purified with NucleoSpin Gel and PCR Clean-Up (740609; Takara). (3) Purified amplicons were inserted into a linearized plasmid with In-Fusion HD Cloning Plus (638909; Takara). The resulting plasmid has the glycine linker introduced from the pooled oligos and an amino acid sequence NSAVDGTAGPGSAT linker after the AH sequence originating from the multiple cloning site of pEGFP-N3, followed by the mNG sequence. Approximately 120 colonies were screened to obtain 57 AH-mNG constructs.

For cloning of established AHs, the following sequences were synthesized as a single-stranded DNA and PCR-amplified: the ALPS-like motif (residues 245–263) of human Nup133 (UniProt ID Q8WUM0^8,25^); AH of human EndophilinA-1^66^; AH of *X. laevis* Magainin2^67^; AH of human Arf1^106^; AH of *S. cerevisiae* Sar1^107^. The AH (ALPS1-ALPS2) of human ArfGAP1 was amplified from pGEX-6P-1-hs-ALPS1-ALPS2-mCherry (a gift from Philipp Niethammer (Addgene plasmid # 187114; RRID: Addgene_187114)^35^). The M domain and P2 AH of human CCTa^68^ were amplified from human CCTa-EGFP (a gift from Neale Ridgway^108^). The ALPS motif of human GMAP210^8^ was amplified from pJAF205 plasmid (a gift from Gregory Pazour (Addgene plasmid; #60407 RRID:Addgene_60407^109^). The amplicons were inserted into the same backbone plasmid as the AH candidates with the sequence encoding the nucleoplasmin NLS.

For recombinant constructs, AH sequences were cloned into a pET-SUMO vector upstream of AcGFP1^110^. The AH DNA sequence was amplified from the corresponding AH-mNG construct and inserted in between SUMO and AcGFP1 with RQAMGG and GGSNSAVDGTAGPGSAT linkers being before and after the AH, respectively.

TMEM214 cDNA (NM_017727) was amplified from U2OS cells and cloned into a pCMVΔ5 vector with a C-terminal HaloTag. Reverse transcription from total RNA using the iScript Reverse Transcription Supermix (1708840; Bio-Rad) was used for cDNA isolation. The cDNA was identical to the transcript variant 1 in the NCBI database (NM_017727).

### Cell culture and transfection

U2OS cells were grown at 37 °C in 5% CO_2_ in DMEM low glucose (11885; Gibco) supplemented with 10% heat inactivated FBS (F4135) and 1% antibiotic–antimycotic (15240112; Gibco). MEF and COS-7 cells were grown at 37 °C in 5% CO_2_ in DMEM high glucose (11965; Gibco) supplemented with 10% heat inactivated FBS (F4135) and 1% antibiotic–antimycotic (15240112; Gibco). HeLa cells were grown in DMEM (Thermo Fisher Scientific; 11960-044) supplemented with 10% FBS (Thermo Fisher Scientific; 10270-106), 1 mM sodium pyruvate (Thermo Fisher Scientific; 11360039), and 1% penicillin–streptomycin (Thermo Fisher Scientific; 10378-016). Cells were used below passage 25 and monitored for contamination using SiR-DNA staining.

DNA transfections were performed with Lipofectamine2000 (11668; Thermo Fisher Scientific) in Opti-MEM (31985; Gibco) with DNA concentrations ranging from 50 to 300 ng DNA per cm^2^ of growth surface. HeLa cells were transfected with 6 µg of plasmid using the Neon transfection device (Thermo Fisher) according to the manufacturer’s instructions and parameters listed for HeLa cells (2 pulses of 35 s at 1005 V). In both cases, cells were imaged 24 h after DNA transfection.

### Generation of TMEM214 knockout cell lines

CRISPR/Cas9-mediated knockout was performed using guide RNAs designed with CRISPick^111,112^. Two sequences with the highest Combined Rank were chosen: #1 AGGTCCTTCAACCATATGGA and #2 TGAGTATGCTGGCTCAGCGT. The guide RNA sequences were synthesized as two oligos with BbsI overhangs and were cloned into pSPCas9(BB)−2A-Puro (PX459) v2.0 (a gift from Feng Zhang, Addgene plasmid #62988; ref. ^113^) that had been digested with BbsI-HF (New England BioLabs #R3539). The vector was transfected into U2OS cells using Lipofectamine2000 and selected with 1 µg/ml puromycin (Invitrogen) for 48 h. The remaining cells were grown up, and genomic DNA was isolated from the bulk population using a QiaAmp DNA Mini kit (Qiagen 51304). Bulk populations were screened by sequencing and TIDE analysis^114^, followed by clonal isolation and validation by immunoblotting and sequencing. One clone from each guide was used for experiments.

### Imaging and microscopy

Live-cell imaging was performed under temperature-, CO₂-, and humidity-controlled conditions. Cells were plated in µ-Slide 8-well Glass Bottom chamber (80827; ibidi) and imaged in appropriate media, with optional SiR-DNA labeling^115,116^. In short, cells were incubated with 250 nM SiR-DNA^117,118^ in the growth media for 1 h prior to imaging.

TMEM214-Halo expressing cells were incubated with 100 nM JF552 HaloTag ligand for 1 h, washed four times with pre-warmed media, then incubated for an additional 10 min in media. Cells were then fixed in 4% paraformaldehyde in PBS for 20 min at room temperature, permeabilized in 0.1% Triton X-100 for 5 min at room temperature, and then blocked in 3% BSA in PBS for 30 min at room temperature. Samples were then incubated with mAb414 (1:500) in 3% BSA in PBS for 2 h at room temperature. Samples were washed with PBS three times, then incubated with anti-Mouse IgG Alexa Flour 647 (Thermo Fisher) in 3% BSA in PBS for 45 min at room temperature with rocking. Samples were then washed with PBS three times. Coverslips were mounted with ProLong Gold Antifade reagent without DAPI.

Airyscan imaging was performed using an LSM 980 confocal microscope (Carl Zeiss Microscopy). A Zeiss Plan-Apochromat 63×, 1.40-NA DIC M27 oil objective was used. The sample was excited by a diode-pumped solid-state laser at 561 and 639 nm (intensities of 2.0% and 0.2%, respectively) and detected using the Airyscan detector. BP 420–480 + BP 570–630 and BP 495–555 + BP 660-750 filters were applied to avoid bleed-through between channels for emission from 561 and 639 nm excitation, respectively. Images were of a size of 1140 × 1140 pixels. Following the acquisition, images were deconvoluted using Airyscan processing with the Standard processing strength. Image processing was performed with ZEN software (version 3.11).

### Osmotic perturbations

Hypotonic treatment was performed by diluting culture media 1:1 with water (~150 mOsm) and imaging within ~3 min.

### Cell stretch experiments

#### Preparation of stretchable PDMS membranes

Stretchable polydimethylsiloxane (PDMS) (Sylgard Silicone Elastomer Kit, Dow Corning) membranes were prepared, as previously described^115,119^. Briefly, a mix of 10:1 base to crosslinker ratio was degassed and spin-coated for 1 min at 500 rpm and cured at 65 °C overnight on methacrylate plates. Once polymerized, membranes were peeled off and assembled onto a metal ring that can subsequently be assembled in the stretch device.

#### Mechanical stimulation of cells and microscopy for live imaging of stretched cells

Cell mechanical stimulation was done as previously described^115^ using a custom-built stretching device that produced uniform biaxial deformations of stretchable PDMS membranes and of overlying cells. Briefly, a 150 μL droplet of a 10 μg/mL fibronectin solution (Sigma, F1141) was deposited at the center of the PDMS membrane mounted in the ring. After overnight incubation at 4 °C, the fibronectin solution was rinsed with PBS (Gibco, 14200-067), and cells were seeded on the fibronectin-coated membranes and allowed to attach for 30–90 min in the presence of Spy650-DNA (1:1000 dilution, Spirochrome, SC501). Before the experiment, the media was exchanged to CO_2_-independent media supplemented with rutin^116^. Then ring-containing membranes were mounted in the stretch system previously described^115,119^. The stretch system was mounted inside an inverted microscope (Nikon Eclipse Ti) equipped with an incubation chamber, and the temperature was kept at 37 °C during the experiment. Images of cells were acquired with a ×60 objective (NIR Apo 60X/WD 2.8, Nikon) on the inverted microscope, with a spinning disk confocal unit (CSU-W1, Yokogawa), a Zyla sCMOS camera (Andor) and using the Micromanager software. For each cell, a z-stack of nuclei was acquired at rest (0.2 μm step) and recorded using the 488 nm excitation wavelength for mNeon-green and 640 nm excitation wavelength for SpyDNA. Several cells (5–6) were imaged at rest (before stretching) in each stretch experiment, and their positions were recorded. After the PDMS substrate was stretched (12–15% substrate strain), substrate movement due to stretch resulted in cell movement refocusing, followed by imaging with the same settings as pre-stretch.

### Image analysis

Subcellular localization of AH constructs was assessed in a blinded manner and categorized based on organelle-like patterns.

NE enrichment was quantified using a custom ImageJ macro^120^. Nuclear regions were segmented using the Li method^121^, and rim versus nucleoplasmic fluorescence intensities were calculated to derive an NE enrichment score (Fig. S10a).

GUV binding assays were quantified using a custom ImageJ macro^120^ with segmentation-based approaches to calculate 'rim'-to-'outside' fluorescence intensity ratios with the 'background' subtracted (see Fig. S10b for a schematic of 'rim', 'outside' and 'background').

Pearson correlation coefficients were calculated using custom Python scripts following segmentation, masking, and normalization. The correlation coefficient between the two channels was calculated using the corrcoef function of the NumPy module.

### Amino acid sequence analyses

Sequence properties, including hydrophobicity and hydrophobic moment, were calculated using custom Python scripts^122^ based on established hydrophobicity scales^123^. Amphipathic helix geometry was analyzed using helical wheel projections. The hydrophobic residues counted for hydrophobic residue density were V, L, I, F, W, M, Y, C, and P.

Mapping of hydrophobic and hydrophilic faces of predicted AHs was performed with custom Python scripts^122^ inspired by HeliQuest^51^.

### Immunoblotting

Cells were lysed in RIPA buffer (25 mM Tris pH 7.4, 1% NP-40, 0.5% sodium deoxycholate, 0.1% SDS, 150 mM NaCl, and 1 tablet/50 ml cOmplete Mini protease inhibitor cocktail [11836153001; Roche]), and proteins were separated by SDS–PAGE and transferred to nitrocellulose membranes. After blocking in 1% BSA, membranes were incubated with primary and secondary antibodies and visualized by chemiluminescence. Clarity ECL reagent (1705060S; Bio-Rad) or ECL select (RPN2235; Cytiva) (in case of low expression level of AH-mNG proteins) was used to visualize chemiluminescence with a Bio-Rad ChemiDoc Imaging System. Antibody concentration was the following: anti-mNG 1:3000; α-tubulin 1:5000; anti-TMEM214 1:1500. Secondary antibodies (goat anti-mouse IgG; 31430, Thermo Fisher Scientific) were used at 1:10,000.

### Recombinant protein purification

6×His-SUMO-tagged AH proteins were expressed in *Escherichia coli* BL21(DE3), purified by Ni-NTA chromatography, and cleaved with recombinant 6xHis-Ulp1 protease^124^ at 4 °C for 1 h following incubation with Ni-NTA agarose to remove the 6xHis-SUMO tag and 6xHis-Ulp. Proteins were buffer-exchanged into PBS using Amicon centrifugal filter MWCO 10 kDa (Millipore Sigma Cat# UFC5010), flash frozen, and stored at −80 °C.

### Giant unilamellar vesicle (GUV) preparation

Giant Unilamellar Vesicles (GUVs) were prepared by polyvinyl alcohol (PVA)-assisted swelling^98,125^. PVA (weight-average molecular weight of 146,000 to 186,000; Cat# 363065; Sigma-Aldrich) was solubilized in water to 5% (w/w) stirred and heated at 90 °C for 1 h and stored at room temperature until used. Forty microliters of PVA solution was spread on a glass coverslip (1.2 cm diameter), which was pre-washed with 70% ethanol and distilled water, and dried at 50 °C for 30 min. PVA-coated coverslips were stored at room temperature. Lipid mixture in chloroform was prepared in a glass tube (Fisher #14-961-26) containing 0.25 mol/mol% of Rhodamine-PE to label GUV. Two microliters of lipid mixture (1 mg/ml) dissolved in chloroform were spread on top of the PVA layer, and chloroform was allowed to evaporate for 1 h. Two hundred microliters of swelling solution (280 mM sucrose) was added to each coverslip placed in a 24-well cell culture dish and incubated for 1 h at room temperature with gentle rocking to induce vesicle formation. The solution containing vesicles was collected and pelleted by centrifugation at 500× *g* for 5 min. Vesicles in pellet were suspended in 80 µL of 1× PBS and kept at room temperature for immediate use. Vesicles were mixed with purified proteins (final protein concentration 500 nM) and immediately imaged in an ibidi eight-well chamber on a spinning disk confocal microscope, as described above. Wells were blocked for 1 h with 2.5 mg ml^−1^ bovine serum albumin in PBS and were washed with 1× PBS before loading the samples.

### Liposome preparation

Liposomes were prepared by lipid film hydration and extrusion, and sizes were measured by dynamic light scattering using DynaProTitan (Wyatt Technology). In short, Lipids were mixed in a glass tube and dried under a flow of nitrogen gas, followed by incubation in a vacuum at room temperature for 1 h. Dried lipid film was incubated in 10 mM Tris-HCl, 100 mM NaF buffer at 37 °C for 1 h, then vortexed at room temperature for 1 min. The resulting liposomes were subjected to five cycles of flash-freezing and thawing. For the size-dependency assay, liposomes were extruded using Avanti Extruder sequentially through polycarbonate membranes of pore size 0.1 µm (Avanti #610005), 0.05 µm (Avanti #610003), and 0.03 μm (Avanti #610002). Liposomes were used within 24 h.

### Circular dichroism

Circular dichroism spectra were collected using purified peptides (>95% purity) under defined conditions using Chirascan (Applied Photophysics). For liposome experiments, one- to six-mM liposomes in 10 mM Tris-HCl, 100 mM NaF buffer were mixed with 0.04 mg/mL peptide and analyzed in a Hellma cuvette with a 0.5 mm path length (Millipore Sigma #Z800058) from 260 to 200 nm at 20 °C. The scan speed was set to 60 nm/min at 1.0 nm intervals. The final spectra were averaged over five to ten scans. Control spectra of buffer and liposomes without peptides were subtracted from the spectra with peptides. Helical content was estimated using MRE at 222 nm with the following equation^126^:

$${Helical\; content}=-({MRE\_}222+2,\,340)/30,\,300\times 100$$

Fitted curve and secondary structure estimate from CD spectra were obtained using BeStSel^127^ (https://bestsel.elte.hu/index.php).

### In silico prediction of *ΔΔF* values

The affinity of each AH sequence for membranes with increased lipid packing defects, quantified by *ΔΔF* values (van Hilten et al.^72^), was predicted using PMIpred (van Hilten et al.^71^) (https://pmipred.fkt.physik.tu-dortmund.de/curvature-sensing-peptide/) with a negatively charged membrane (20% PG) as the target. Note that PMIpred can be calculated for AHs 7-24 amino acids and thus AHs with >=25 aa were eliminated from the analysis. This analysis yielded ΔΔF value for a neutral (PC alone) membrane (ΔΔF_neutral_membrane) as well. Since the *ΔΔF* for negatively charged membrane (ΔΔF_adj) from PMIpred is adjusted to 24 aa length, we calculated *ΔΔF* for negatively charged membrane without the length adjustment (ΔΔF_negative_charge) using the following formula from van Hilten et al.^71^ using a custom Python script.

$$\varDelta \varDelta {F\_negative\_charge}=\varDelta \varDelta {F\_neutral\_membrane}+{C\_z}*Z$$

where *C_z* = − 0.93 kJ mol and *z* is the net charge of the helix.

### Analysis of the ALPS-like motif list from Drin et al.^8^

The list of proteins with putative ALPS-like motifs was obtained from the Supplementary Table 1 in ref. ^8^. UniProt IDs in the list were validated in UniProt using API, which resulted in the removal of 3 obsolete IDs. The remaining, valid proteins (193 in total) were crossed with our lists of NE proteins and predicted AHs.

### AlphaFold predicted structure analysis

AlphaFold structure predictions were downloaded from AlphaFold Protein Structure Database [https://alphafold.ebi.ac.uk/], model v4 (accessed on June 15, 2025). Structure data in a PDB file with the AH(s) highlighted or colored by pLDDT were visualized using Pymol version 2.5.0, and Predicted Aligned Error (PAE) data were visualized using PAE viewer^128^ (no versions) [https://pae-viewer.uni-goettingen.de/].

### Statistical analysis and reproducibility

No data were excluded from the analyses. No statistical method was used to predetermine sample size. The experiments were not randomized because appropriate controls were used, and controls and experimental groups were treated in the same manner. Double blind image analysis was performed for categorizing the amphipathic helix localization patterns (Fig. 1b). For all other experiments, the Investigators were not blinded to allocation during experiments and outcome assessment because appropriate controls were used, and each experiment received equal treatment.

GraphPad Prism 8 was used for all statistical and correlation analyses otherwise specified in “Methods”. Box plots are shown by the Tukey method (the middle line: median; box: 25th and 75th percentiles; whiskers: the largest/smallest values or 25th/75th percentiles −/+ 1.5IQR; dots: outliers). Sample sizes and definitions of n and N are reported in Figures and/or Figure legends. For the Box–Cox transformation, the median values of NE enrichment scores were shifted by adding 1 to avoid negative values after transformation, then were transformed using the boxcox function of the SciPy Stats module of Python with the following equation:

*x*_transformed = (*x*^lambda – 1)/lambda

where the optimal lambda value was determined to be −2.44 for our dataset.

Linear Discriminant Analysis (LDA) was performed using a custom Python script^122^. Briefly, the LinearDiscriminantAnalysis classifier of the Discriminant_analysis submodule from the Scikit-Learn module was used to fit the dataset with the number of components = 1. Coefficient values were extracted with the coef_ attribute.

Partial Least Square Analysis was performed using a custom Python script^122^. Briefly, the PLSRegression method of the cross_decomposition submodule from the Scikit-Learn module was used to fit the dataset with the number of components = 1. The dataset was scaled using the StandardScaler of the Preprocessing submodule of the Scikit-Learn module.

### Reporting summary

Further information on research design is available in the Nature Portfolio Reporting Summary linked to this article.

## Supplementary information


Supplementary Information
Transparent Peer Review file
Reporting Summary
Description of Additional Supplementary Files
Supplementary Data 1-17


## Source data


Source Data


## Data Availability

The source data underlying all graphs and charts are provided with this paper. Raw imaging data underlying the figures, as well as sequencing data used for validation of knockout clones, have been deposited in FigShare and are available at [https://doi.org/10.6084/m9.figshare.32608785]. Source data are provided with this paper.

## Code availability

Python and ImageJ Macro scripts are available at GitHub and posted on Zenodo [https://zenodo.org/records/20312987]; [https://zenodo.org/records/20331385]; [https://zenodo.org/records/20312788]; [https://zenodo.org/records/20313035]^99,102,120,122^.
