# Supplementary material for: Screening of amphipathic helices identifies features linked to inner nuclear membrane properties
Source: Nat Commun. 2026 Jul 30;17:9316. doi: 10.1038/s41467-026-75561-0 (PMC13530154; doi:10.1038/s41467-026-75561-0)
Supplement: Supplementary file 1 [file 41467_2026_75561_MOESM1_ESM.pdf]

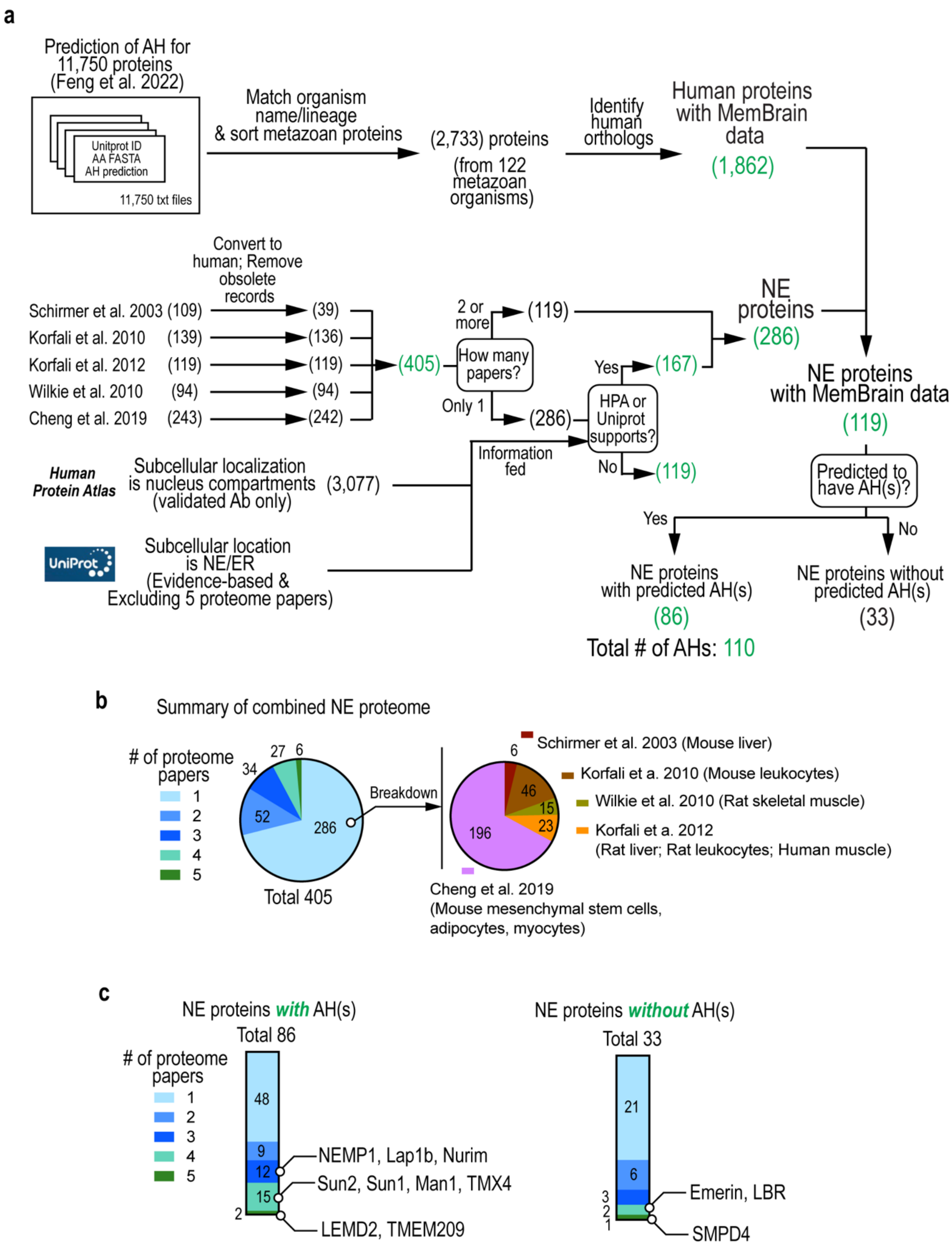

a
Prediction of AH for 11,750 proteins (Feng et al. 2022)
Unitprot ID
AA FASTA
AH prediction
11,750 txt files
Match organism name/lineage & sort metazoan proteins
(2,733) proteins (from 122 metazoan organisms)
Identify human orthologs
Human proteins with MemBrain data
(1,862)
Convert to human; Remove obsolete records
Schirmer et al. 2003 (109) (39)
Korfali et al. 2010 (139) (136)
Korfali et al. 2012 (119) (119)
Wilkie et al. 2010 (94) (94)
Cheng et al. 2019 (243) (242)
(405)
How many papers?
2 or more (119)
Only 1 (286)
HPA or Uniprot supports?
Yes (167)
No (119)
NE proteins (286)
NE proteins with MemBrain data (119)
Predicted to have AH(s)?
Yes
No
NE proteins with predicted AH(s) (86)
NE proteins without predicted AH(s) (33)
Total # of AHs: 110
Human Protein Atlas
Subcellular localization is nucleus compartments (validated Ab only) (3,077)
Information fed
UniProt
Subcellular location is NE/ER (Evidence-based & Excluding 5 proteome papers)
b
Summary of combined NE proteome
# of proteome papers
1
2
3
4
5
286
52
34
27
6
Total 405
Breakdown
6
46
15
23
196
Schirmer et al. 2003 (Mouse liver)
Korfali et a. 2010 (Mouse leukocytes)
Wilkie et al. 2010 (Rat skeletal muscle)
Korfali et a. 2012 (Rat liver; Rat leukocytes; Human muscle)
Cheng et al. 2019 (Mouse mesenchymal stem cells, adipocytes, myocytes)
c
NE proteins with AH(s)
Total 86
# of proteome papers
1
2
3
4
5
48
9
12
15
2
NEMP1, Lap1b, Nurim
Sun2, Sun1, Man1, TMX4
LEMD2, TMEM209
NE proteins without AH(s)
Total 33
21
6
3
2
1
Emerin, LBR
SMPD4

Fig. S1. Computational screen of AHs in putative nuclear envelope-associated proteins proteins.

a. Detailed flow chart of data mining for putative NE proteins and identification of predicted AHs. Numbers in parenthesis indicate the number of proteins. **b.** Breakdown of the 405 NE proteins by the number of the NE proteome papers. **c.** Breakdown of NE proteins with or without a predicted AH by the number of NE proteome papers.

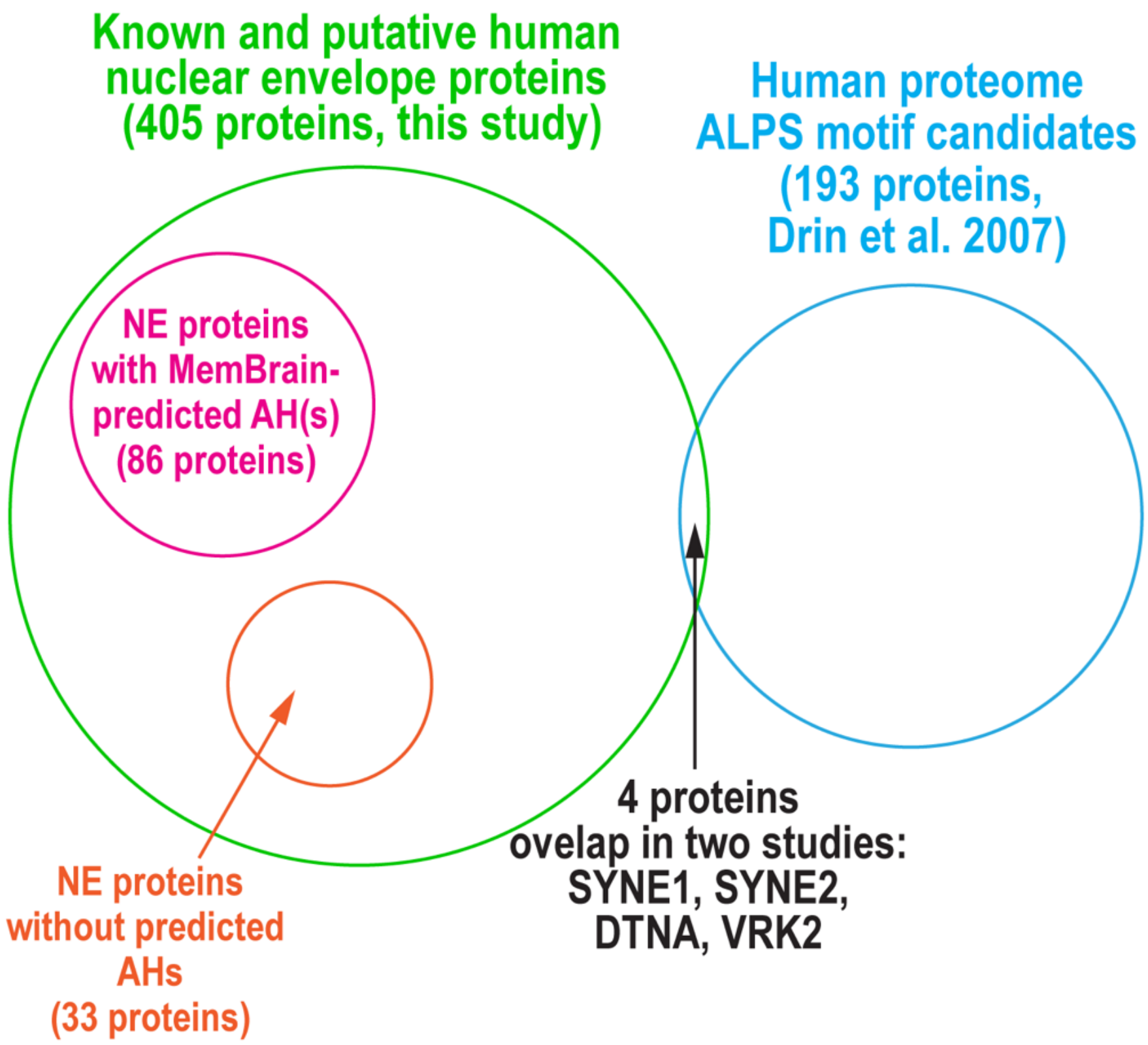

Fig. S2. Overlap of candidate AHs in this study and predicted ALPS-like motifs uncovered in Drin et al. [1]. Venn diagram representing putative NE proteins from this study, with or without a MemBrain-predicted AH, and proteins with a putative ALPS-like motifs [1].

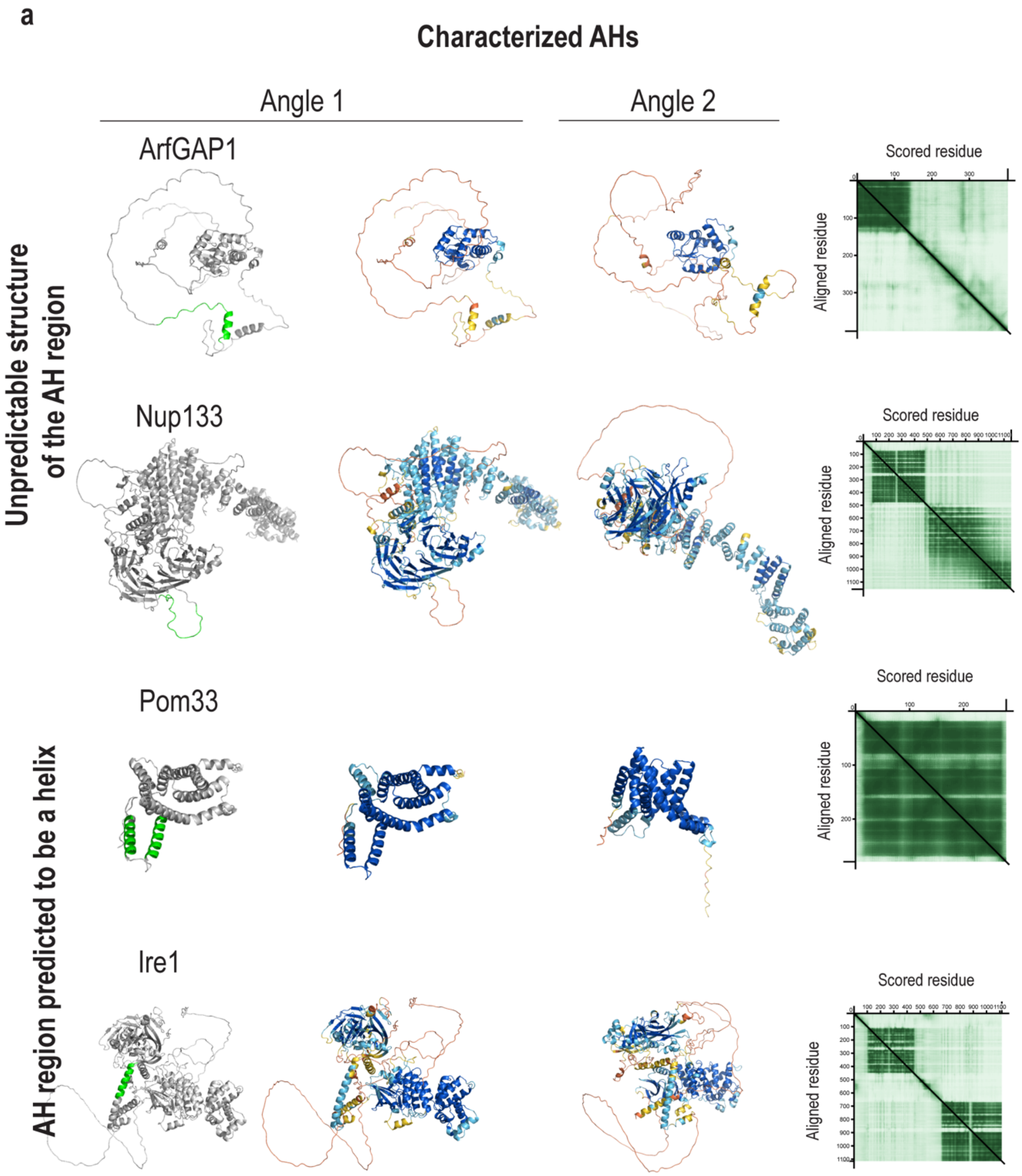
a
Characterized AHs
Angle 1
Angle 2
Unpredictable structure of the AH region
AH region predicted to be a helix
ArfGAP1
Nup133
Pom33
Ire1
Scored residue
Aligned residue

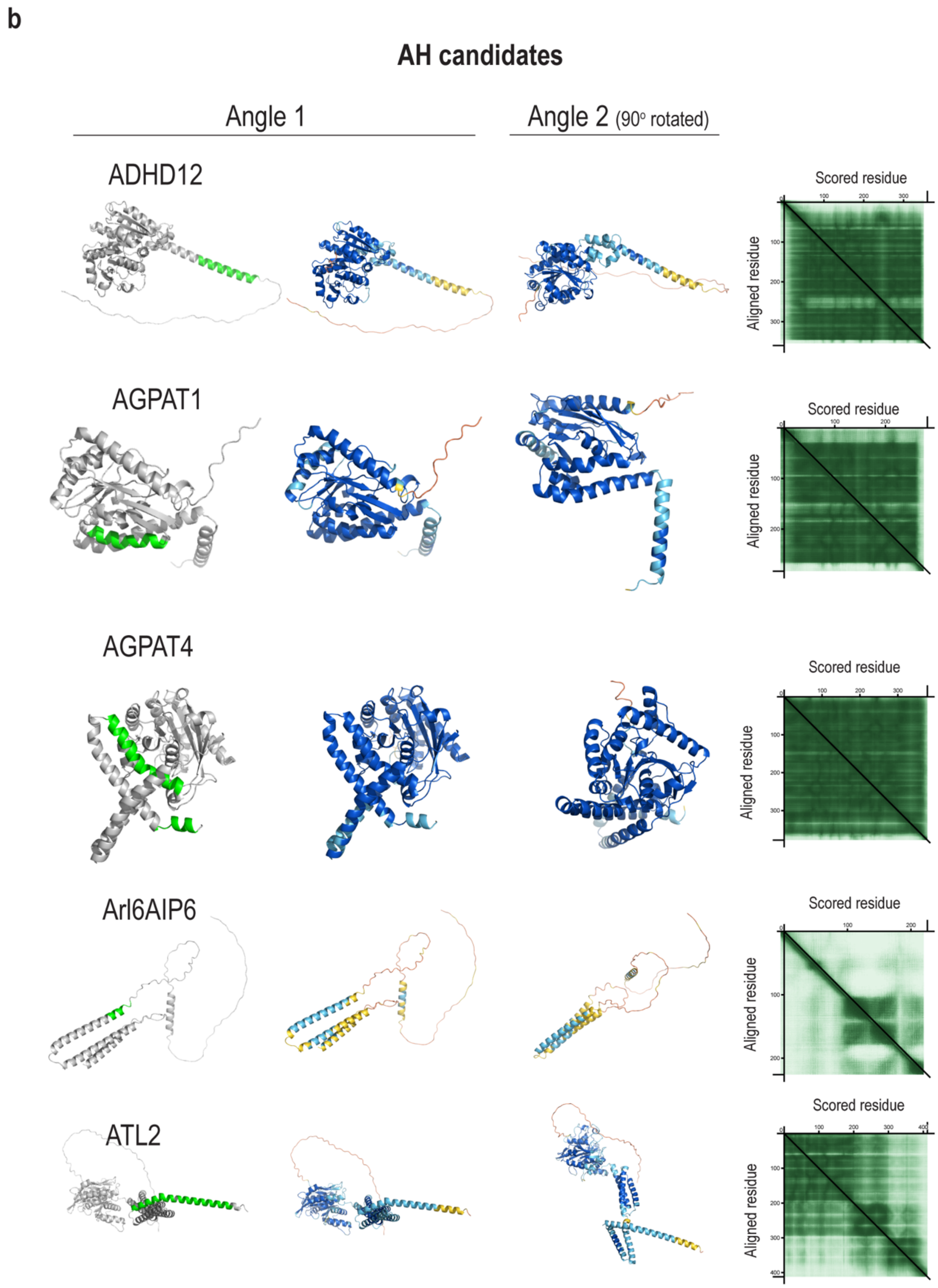

b
AH candidates
Angle 1
Angle 2 (90° rotated)
ADHD12
AGPAT1
AGPAT4
Arl6AIP6
ATL2
Scored residue
Aligned residue

c

AH candidates

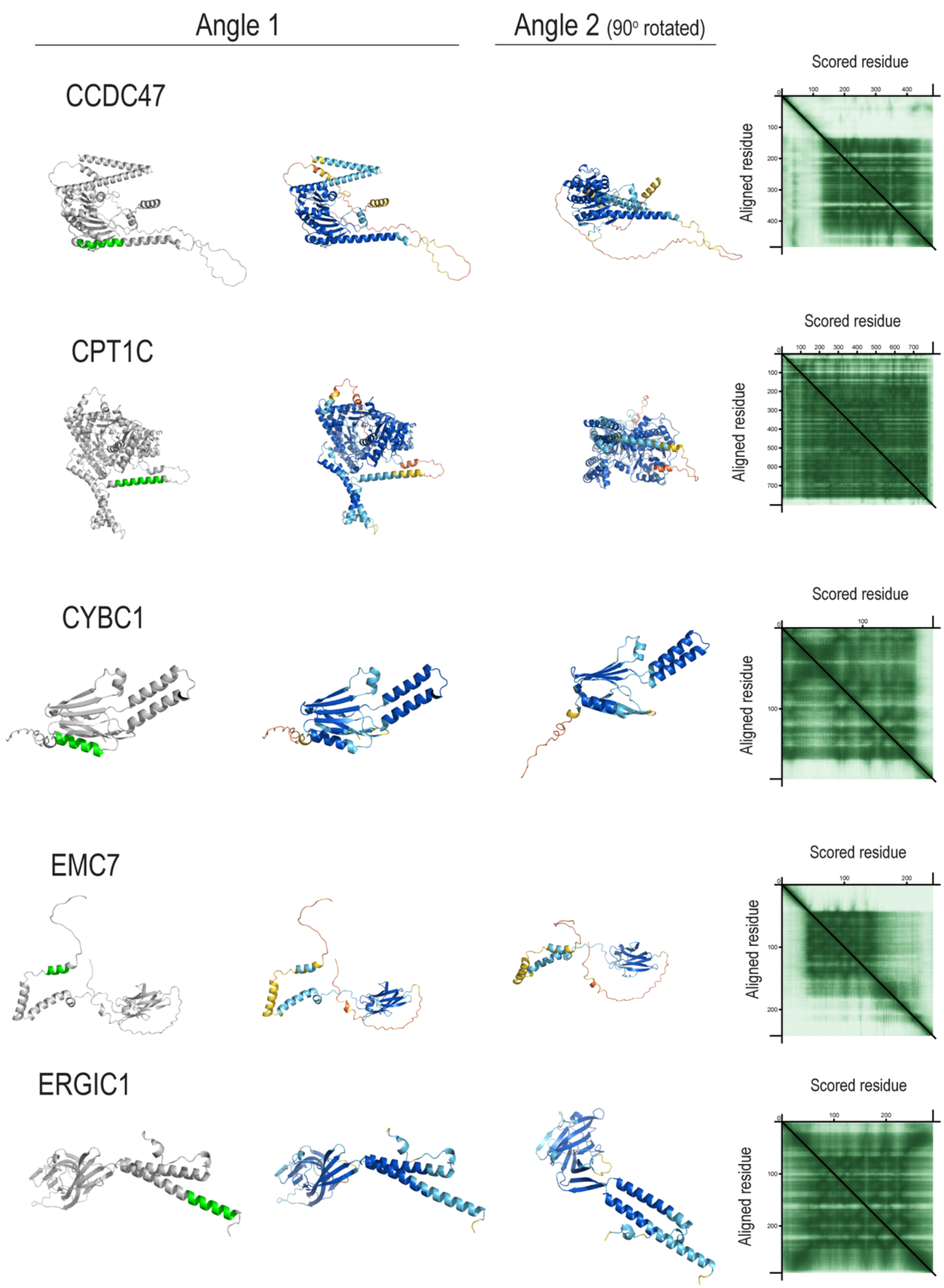

d

AH candidates

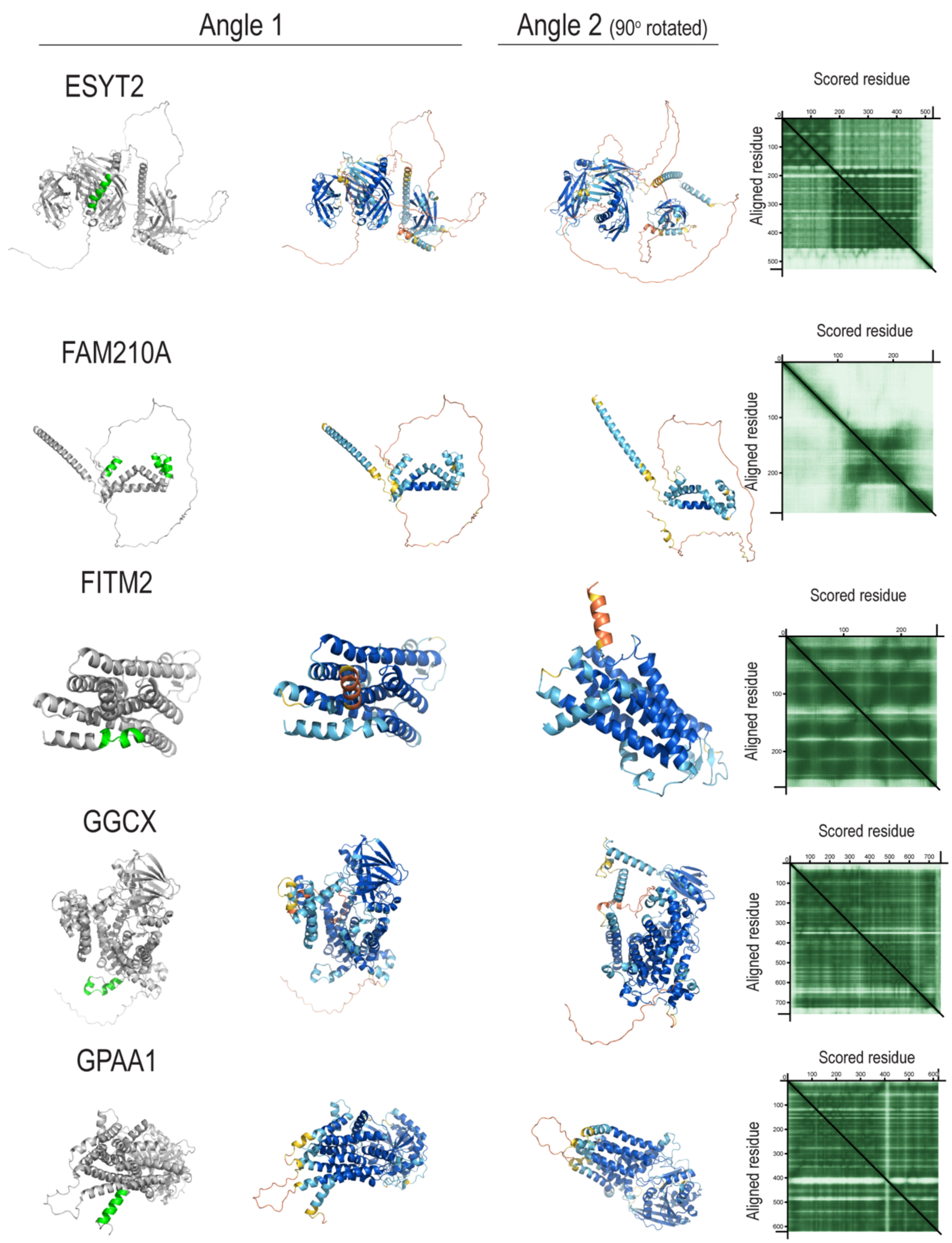

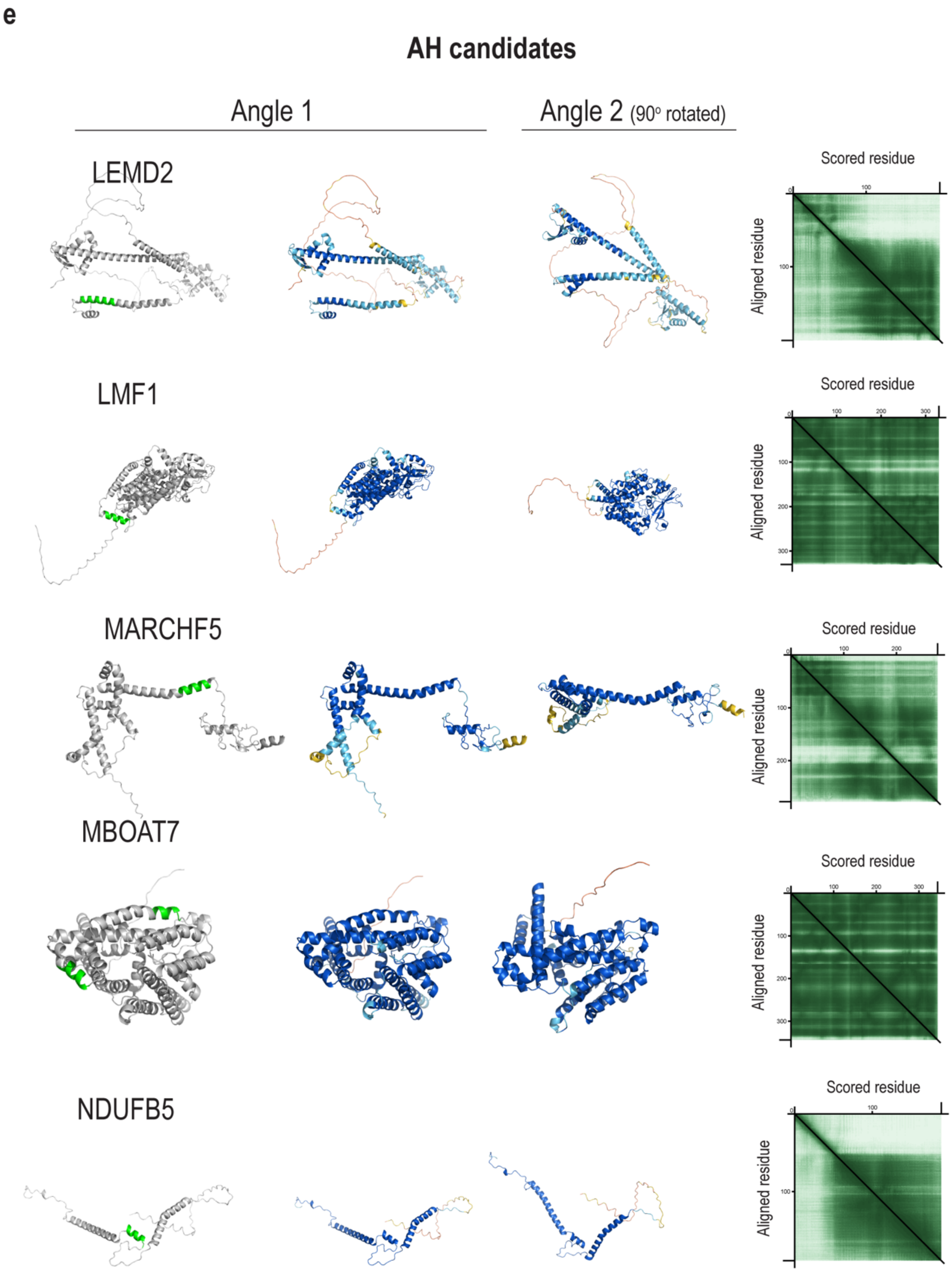
e
AH candidates
Angle 1
Angle 2 (90° rotated)
LEMD2
LMF1
MARCHF5
MBOAT7
NDUFB5
Scored residue
Aligned residue

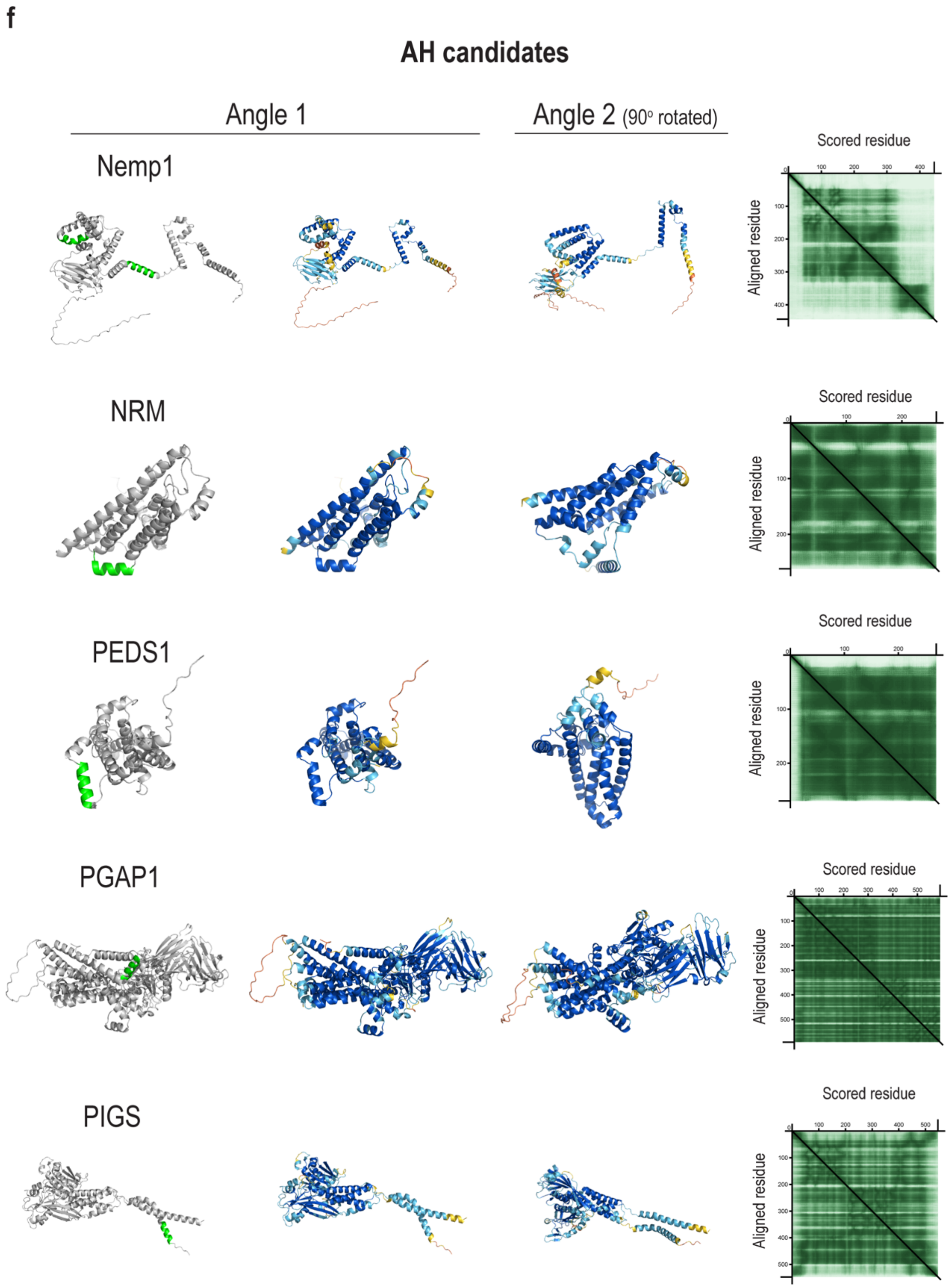
f
AH candidates
Angle 1
Angle 2 (90° rotated)
Nemp1
NRM
PEDS1
PGAP1
PIGS
Scored residue
Aligned residue

g

AH candidates

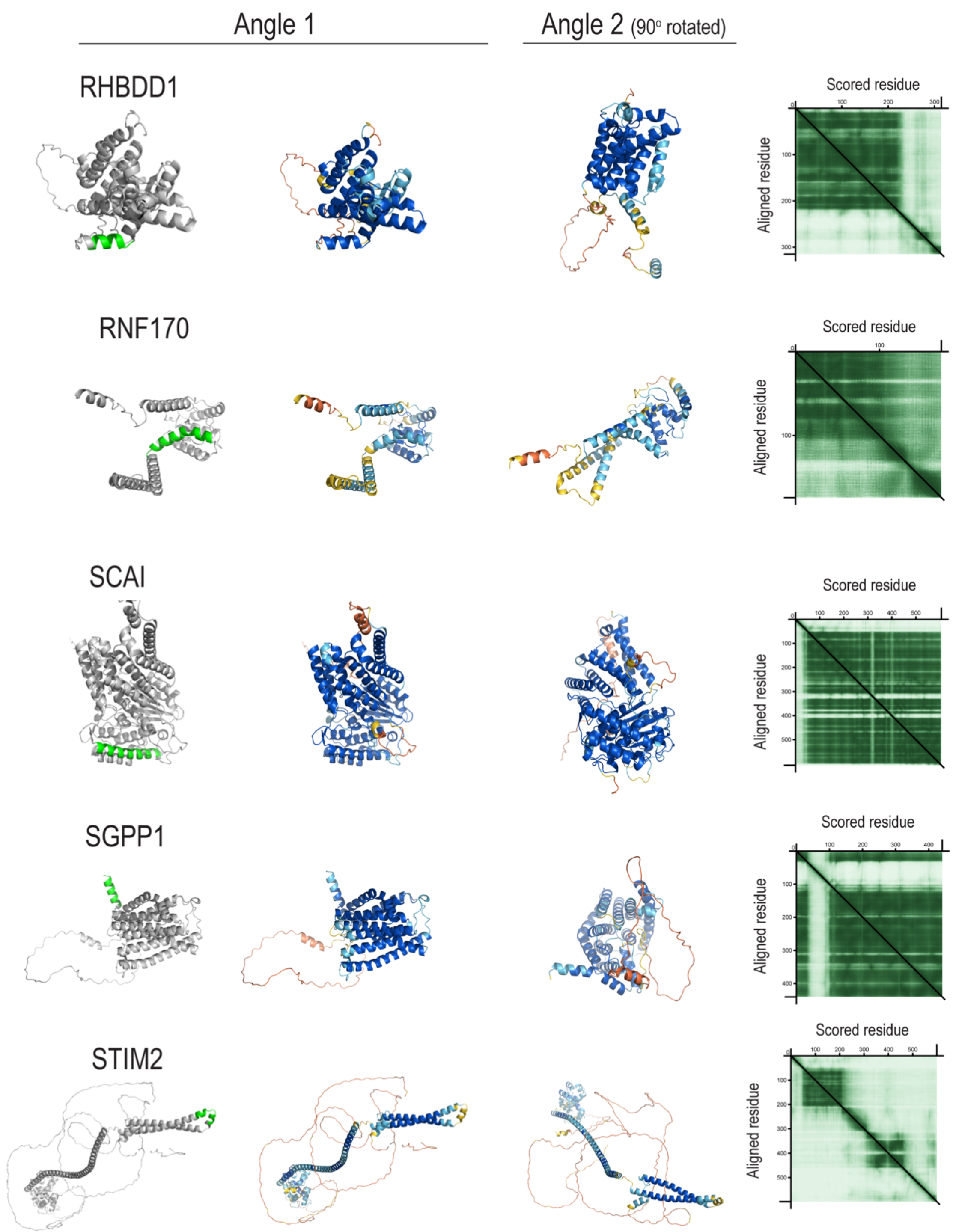

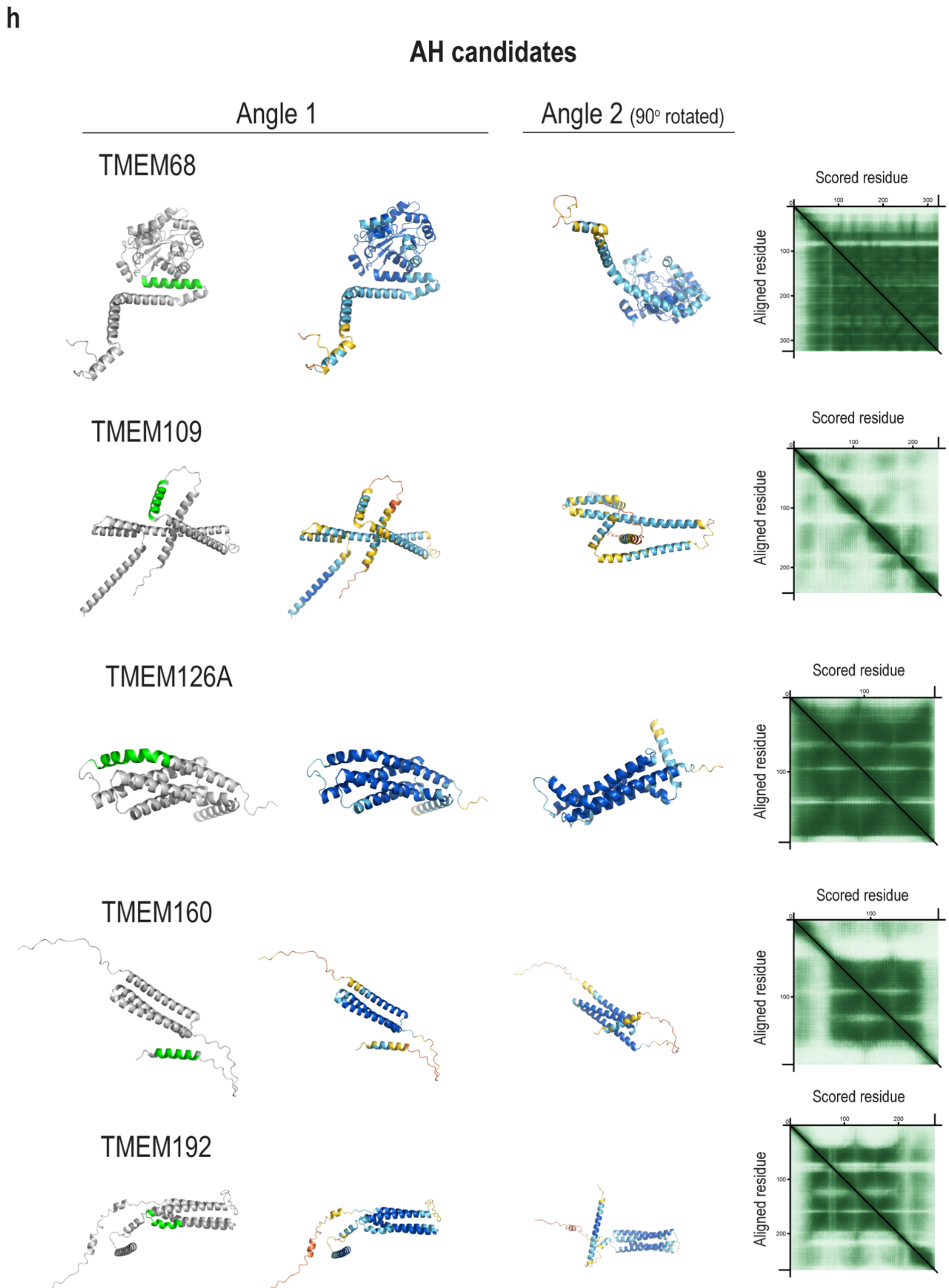
h
AH candidates
Angle 1
Angle 2 (90° rotated)
TMEM68
Scored residue
Aligned residue
TMEM109
Scored residue
Aligned residue
TMEM126A
Scored residue
Aligned residue
TMEM160
Scored residue
Aligned residue
TMEM192
Scored residue
Aligned residue

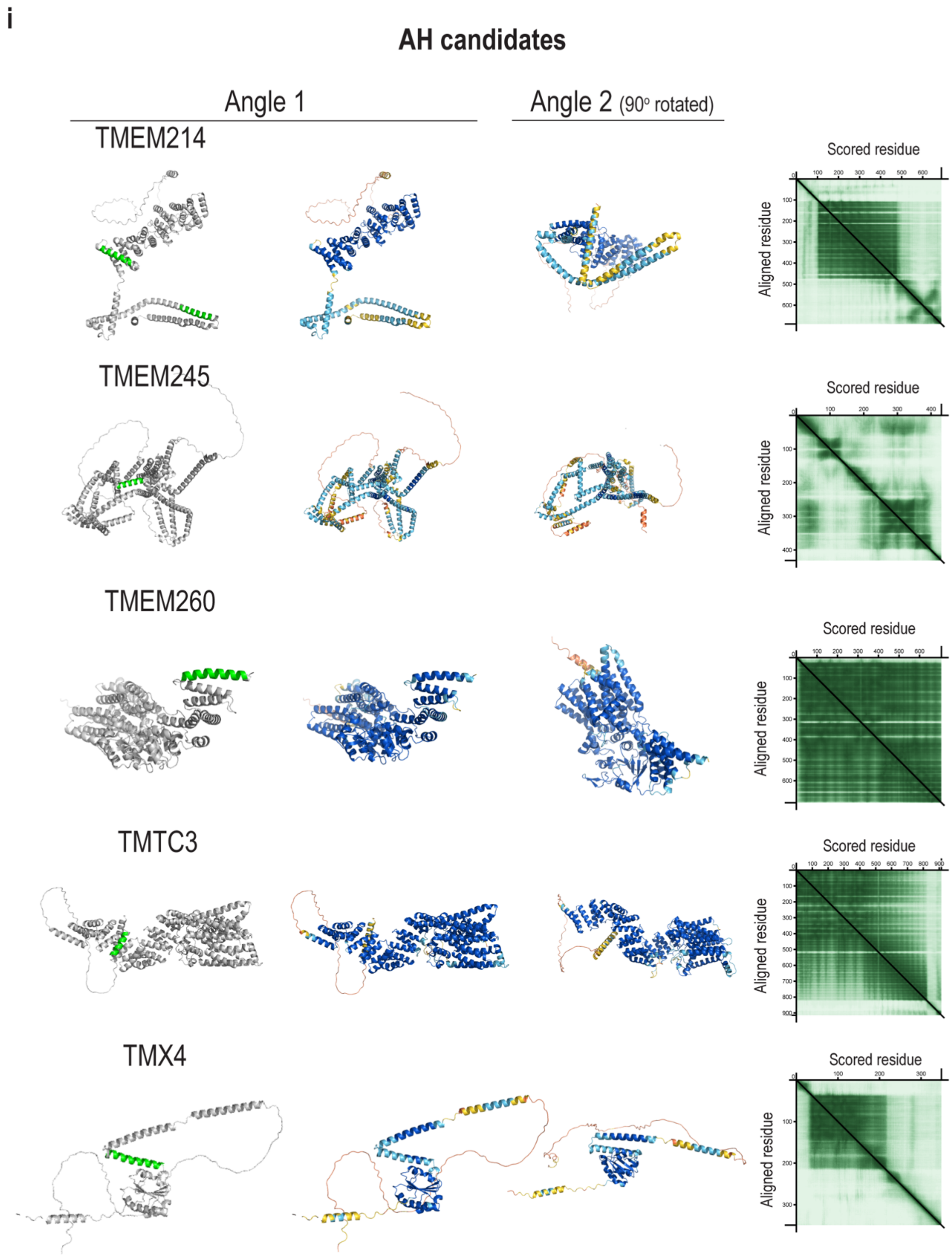
i
AH candidates
Angle 1
Angle 2 (90° rotated)
TMEM214
TMEM245
TMEM260
TMTC3
TMX4
Scored residue
Aligned residue

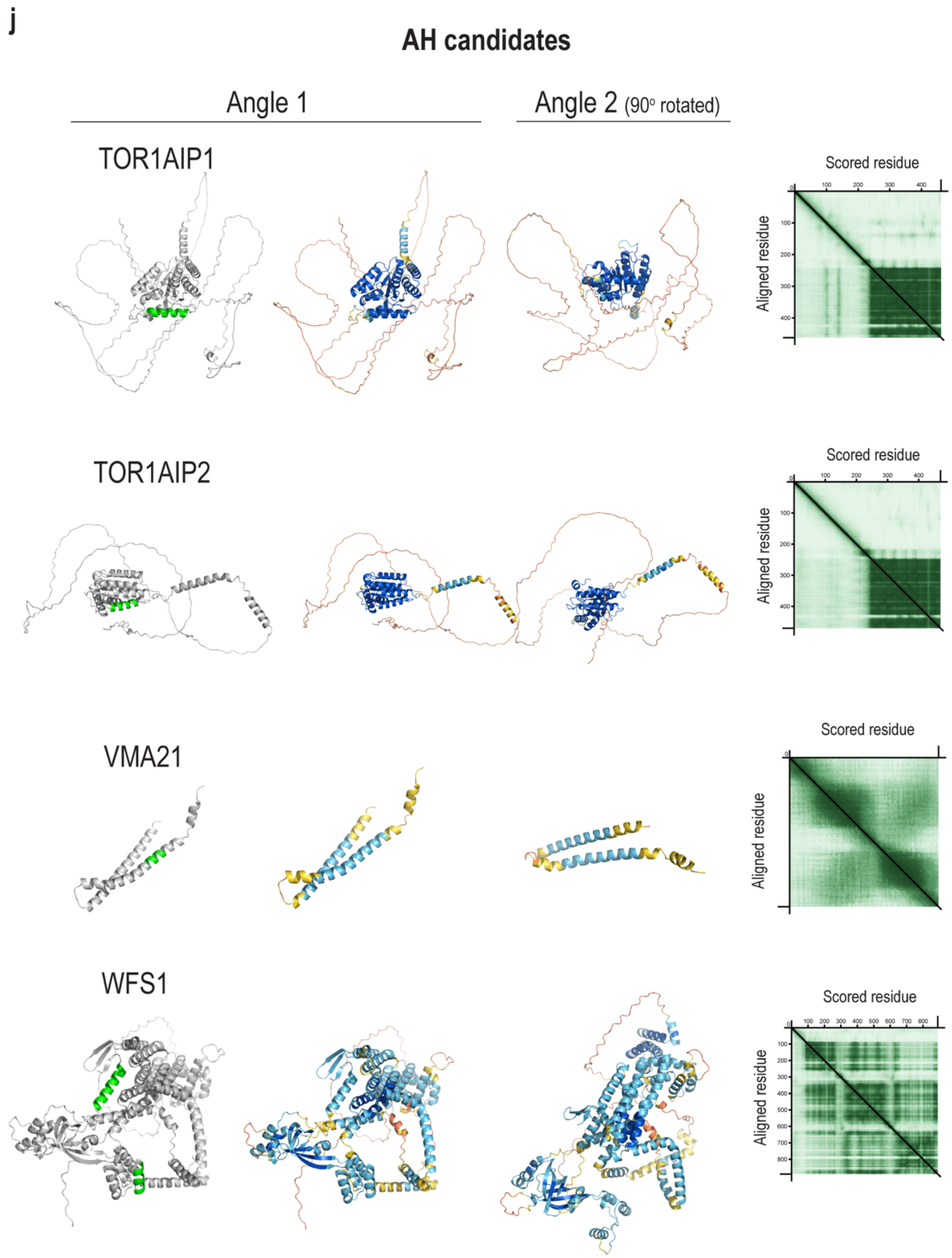
j
AH candidates
Angle 1
Angle 2 (90° rotated)
TOR1AIP1
Scored residue
Aligned residue
TOR1AIP2
Scored residue
Aligned residue
VMA21
Scored residue
Aligned residue
WFS1
Scored residue
Aligned residue

Fig. S3. AlphaFold predictions of established and candidate AHs in the context of their protein backbone.

a-j. Left, Pymol representations of AHs (green) in context of protein backbone (gray). Middle, protein prediction with pLDDT scores are color-coded (Blue: pLDDT > 90; Cyan: 90 > pLDDT > 70; Yellow: 70 > pLDDT > 50; Orange: pLDDT < 50) shown from two angles. Right, Predicted Aligned Error (PAE) scores (darkest green: 0, white: 32) are shown.

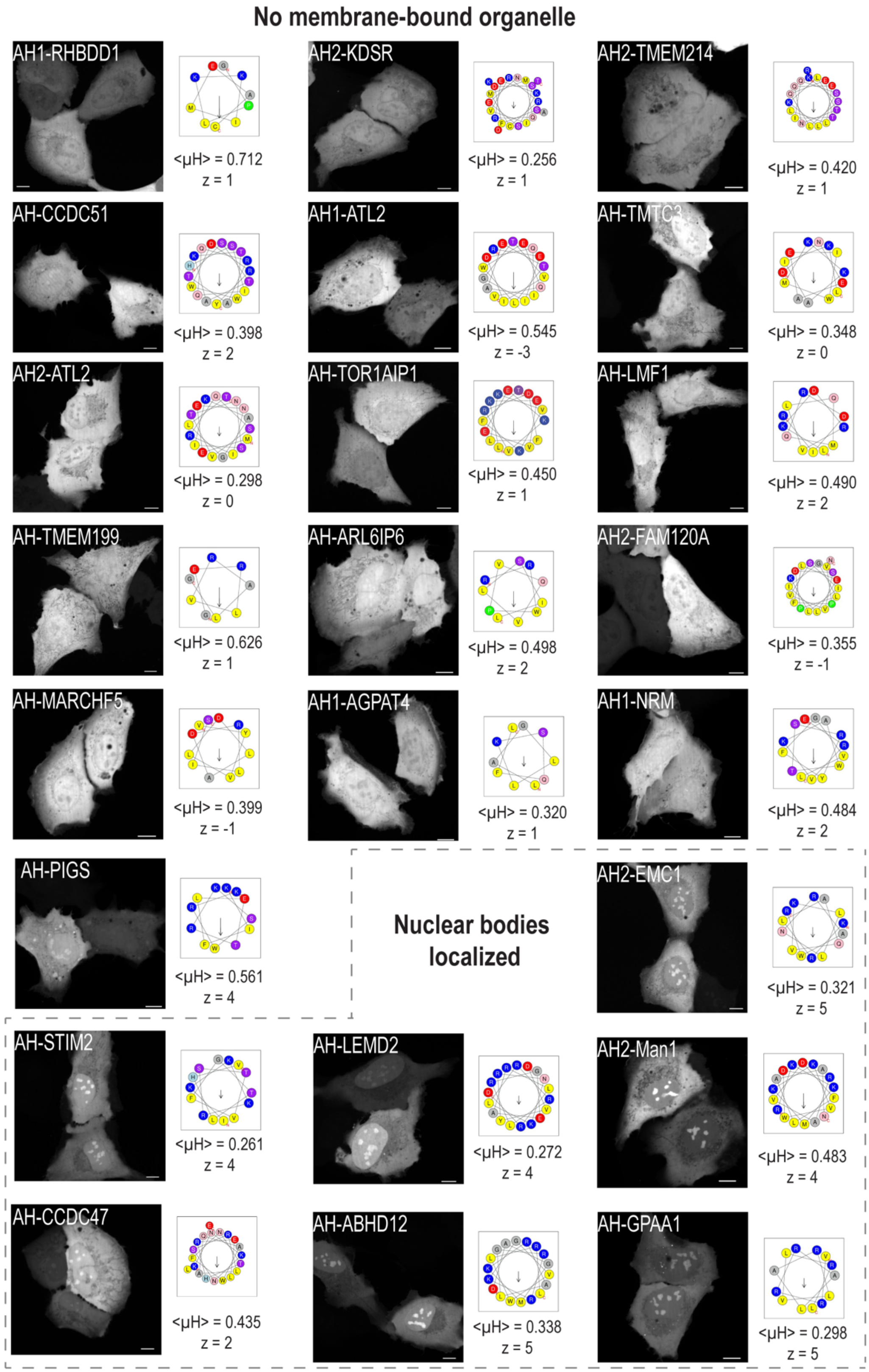
No membrane-bound organelle
AH1-RHBDD1
<μH> = 0.712
z = 1
AH2-KDSR
<μH> = 0.256
z = 1
AH2-TMEM214
<μH> = 0.420
z = 1
AH-CCDC51
<μH> = 0.398
z = 2
AH1-ATL2
<μH> = 0.545
z = -3
AH-TMTC3
<μH> = 0.348
z = 0
AH2-ATL2
<μH> = 0.298
z = 0
AH-TOR1AIP1
<μH> = 0.450
z = 1
AH-LMF1
<μH> = 0.490
z = 2
AH-TMEM199
<μH> = 0.626
z = 1
AH-ARL6IP6
<μH> = 0.498
z = 2
AH2-FAM120A
<μH> = 0.355
z = -1
AH-MARCHF5
<μH> = 0.399
z = -1
AH1-AGPAT4
<μH> = 0.320
z = 1
AH1-NRM
<μH> = 0.484
z = 2
AH-PIGS
<μH> = 0.561
z = 4
Nuclear bodies localized
AH2-EMC1
<μH> = 0.321
z = 5
AH-STIM2
<μH> = 0.261
z = 4
AH-LEMD2
<μH> = 0.272
z = 4
AH2-Man1
<μH> = 0.483
z = 4
AH-CCDC47
<μH> = 0.435
z = 2
AH-ABHD12
<μH> = 0.338
z = 5
AH-GPAA1
<μH> = 0.298
z = 5

Fig. S4. Subcellular localization patterns of AH candidates tagged with mNG that did not localize to membrane-bound organelles.

Representative spinning disk confocal images of a single section in living U2OS cells expressing the indicated AH-mNG constructs are shown. Experiments were conducted two or three times with similar results. Schematic representations of corresponding helical wheel projections of the predicted AHs with mean hydrophobic moment <μH> and net charge z is shown. AH-mNG proteins with a nuclear body-like pattern are highlighted at the bottom right. Scale bars, 10 μm.

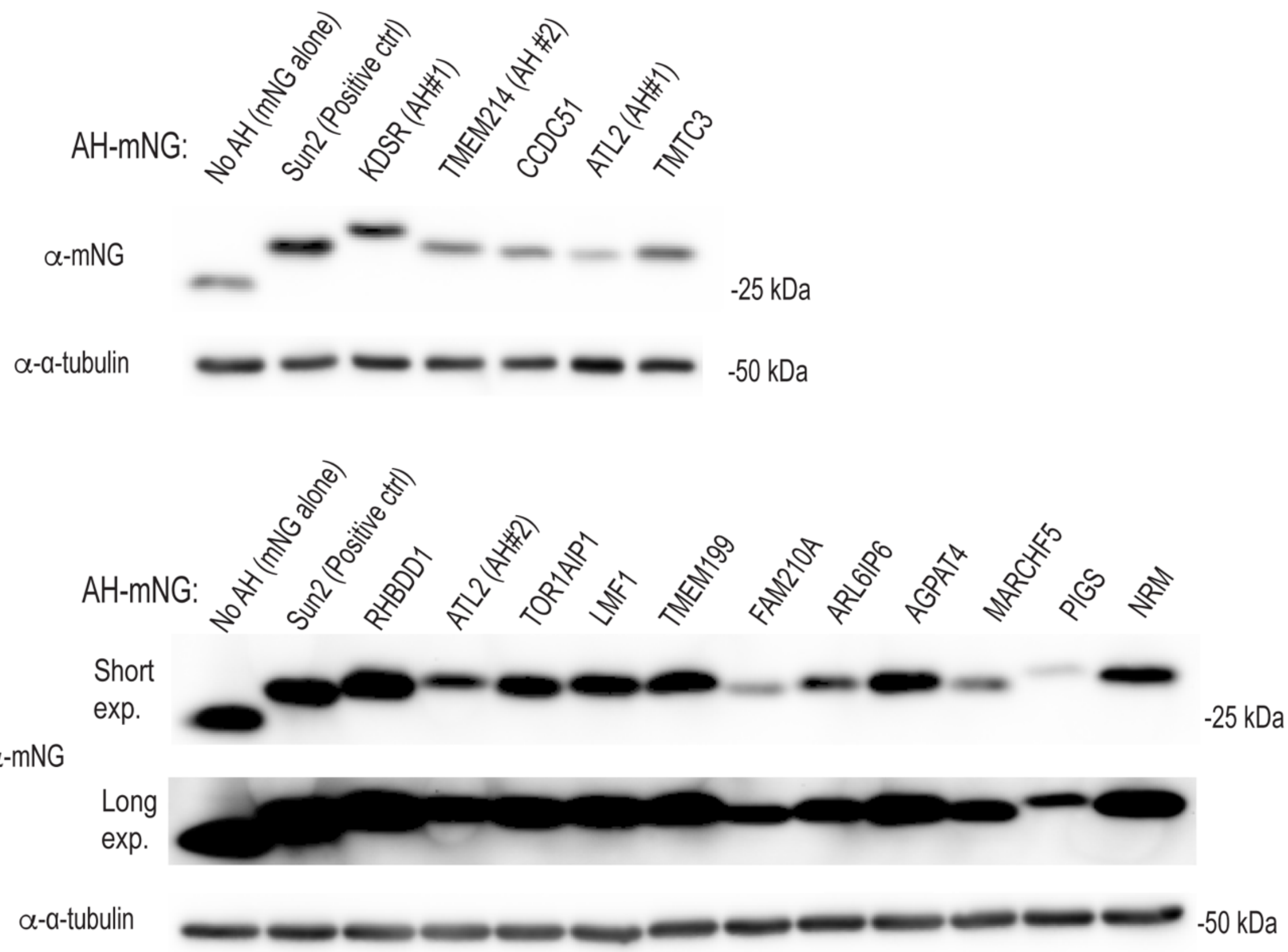

Fig. S5. AH-mNG that do not localize to membrane-bound organelles or nuclear bodies are expressed true to size.

Immunoblot of mNG alone or the indicated AH-mNG proteins transiently expressed in U2OS cells. Some of the AH-mNG proteins without membrane-bound organelle localization shown in Fig. S4 were chosen for immunoblot analysis. Experiments were conducted two or three times with similar results. Unprocessed blot images are provided in Source Data files.

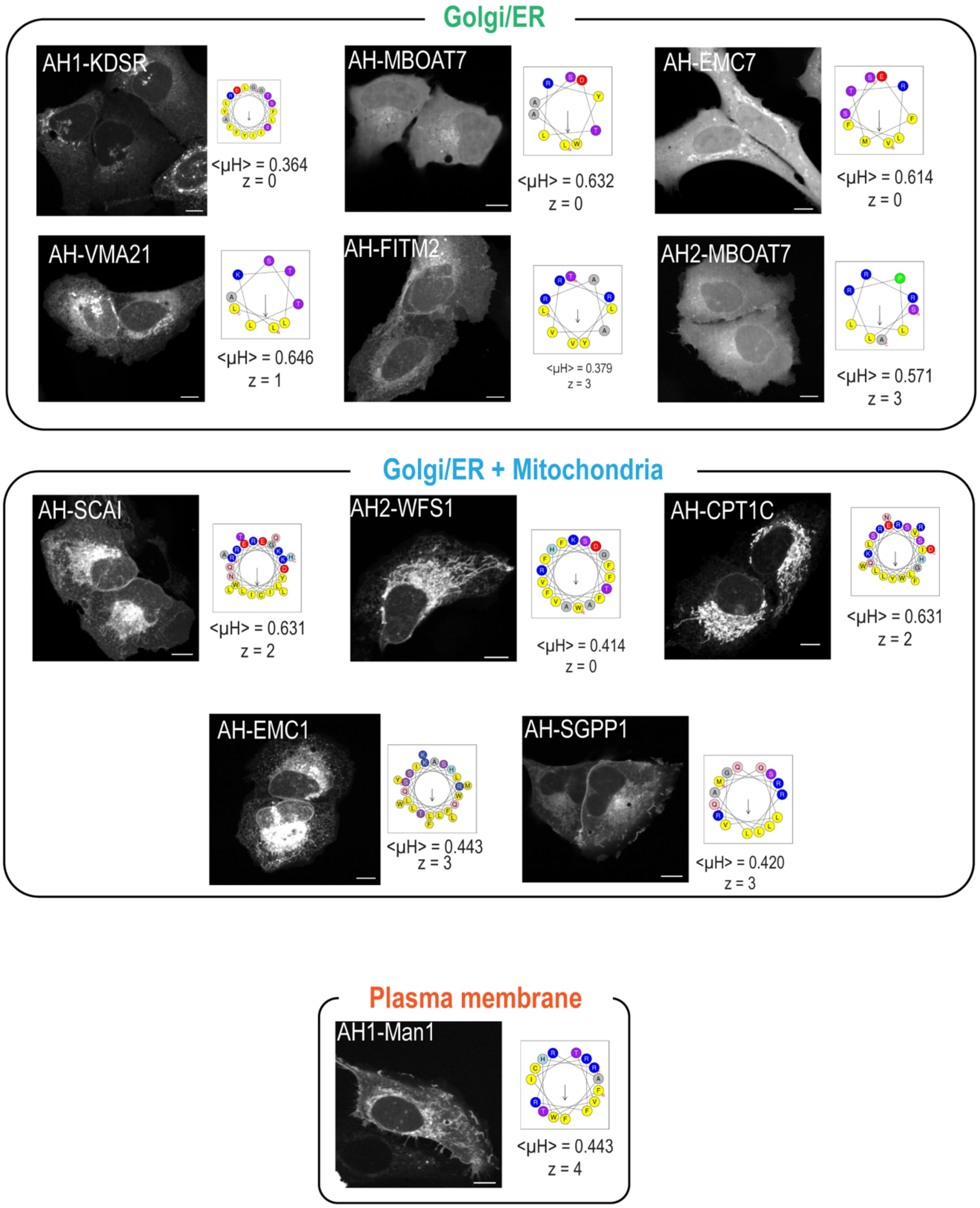

Fig. S6. Subcellular localizations of select AH candidates.

Representative spinning disk confocal images of a single section in living U2OS cells expressing the indicated AH-mNG protein are shown. Experiments were conducted two or three times with similar results. Schematic representations of corresponding helical wheel projections of the predicted AHs with mean hydrophobic moment <μH> and net charge z is shown. Scale bars, 10 μm.

Mitochondria

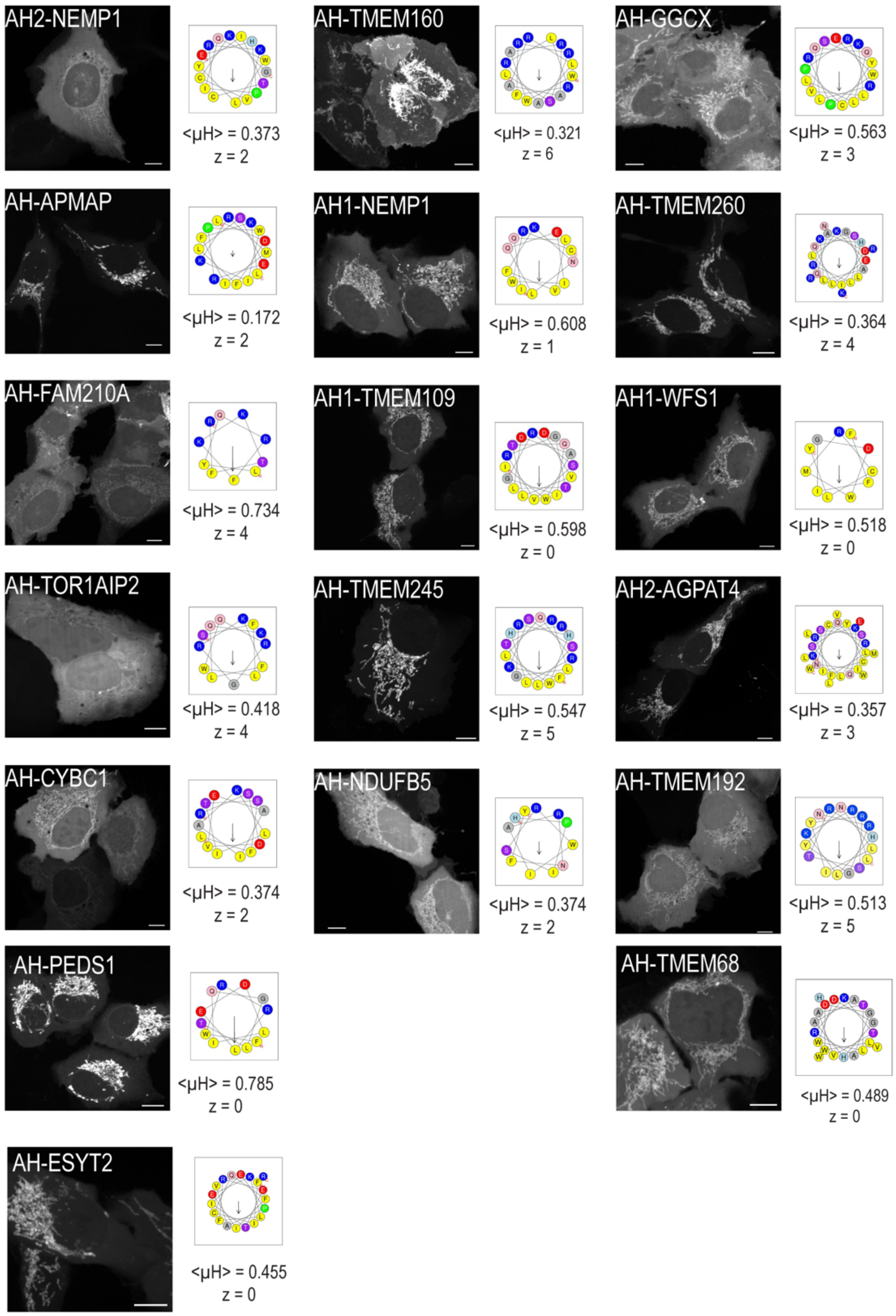

Fig. S7. Subcellular localization of AH candidates tagged with mNG that displayed exclusively mitochondria-like localization (mito-only). Representative spinning disk confocal images of a single section in living U2OS cells expressing the indicated AH-mNG protein are shown. Experiments were conducted two or three times with similar results. Schematic representations of corresponding helical wheel projections of the predicted AHs with mean hydrophobic moment $\langle \mu H \rangle$ and net charge z is shown. Scale bars, 10 μm.

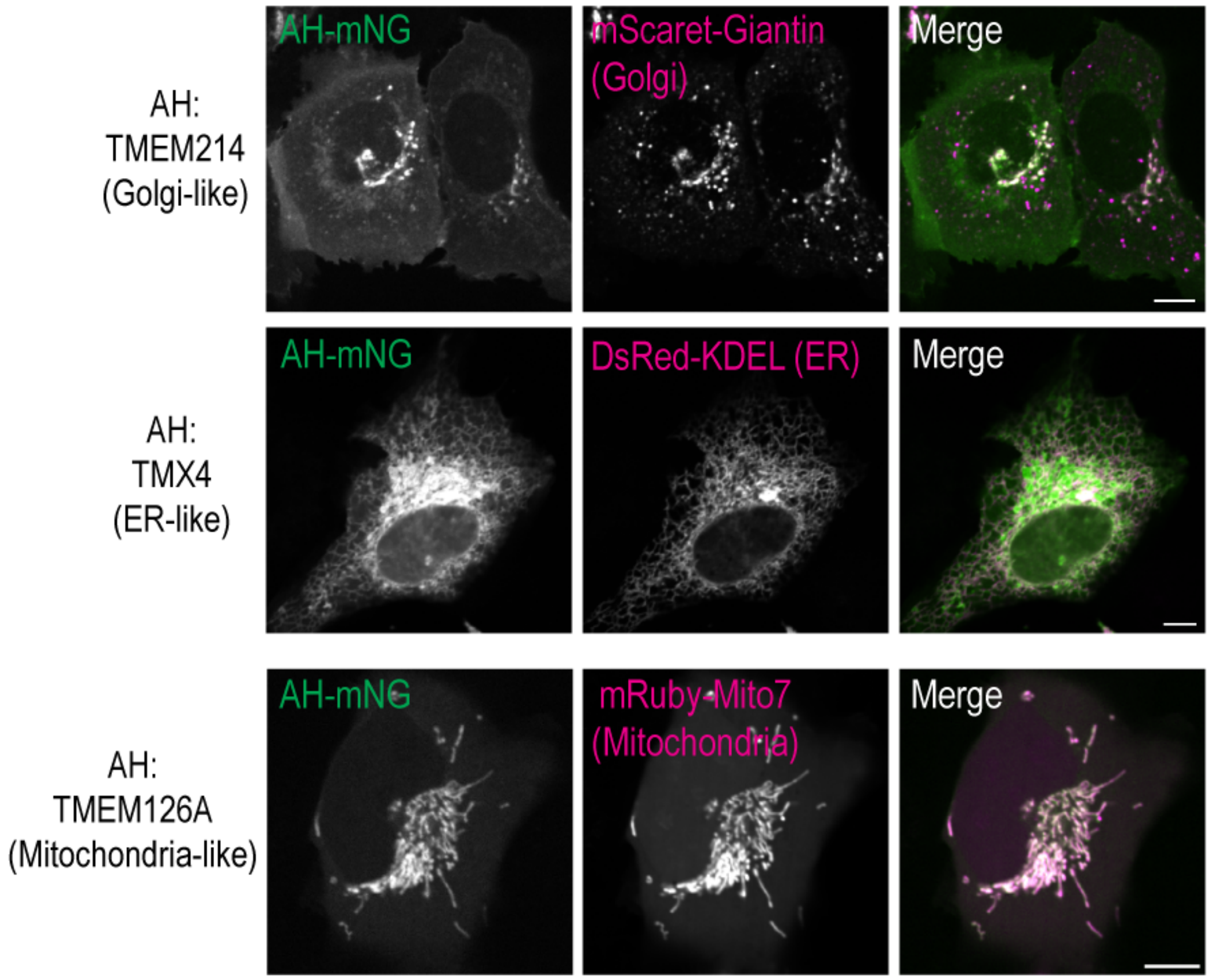

Fig. S8. Co-localization of AH candidates with organelle markers.

Representative spinning disk confocal images of a single section in living U2OS cells co-expressing the indicated AH-mNG and the indicated organelle marker are shown. Experiments were conducted two times with similar results. Scale bars, 10 µm.

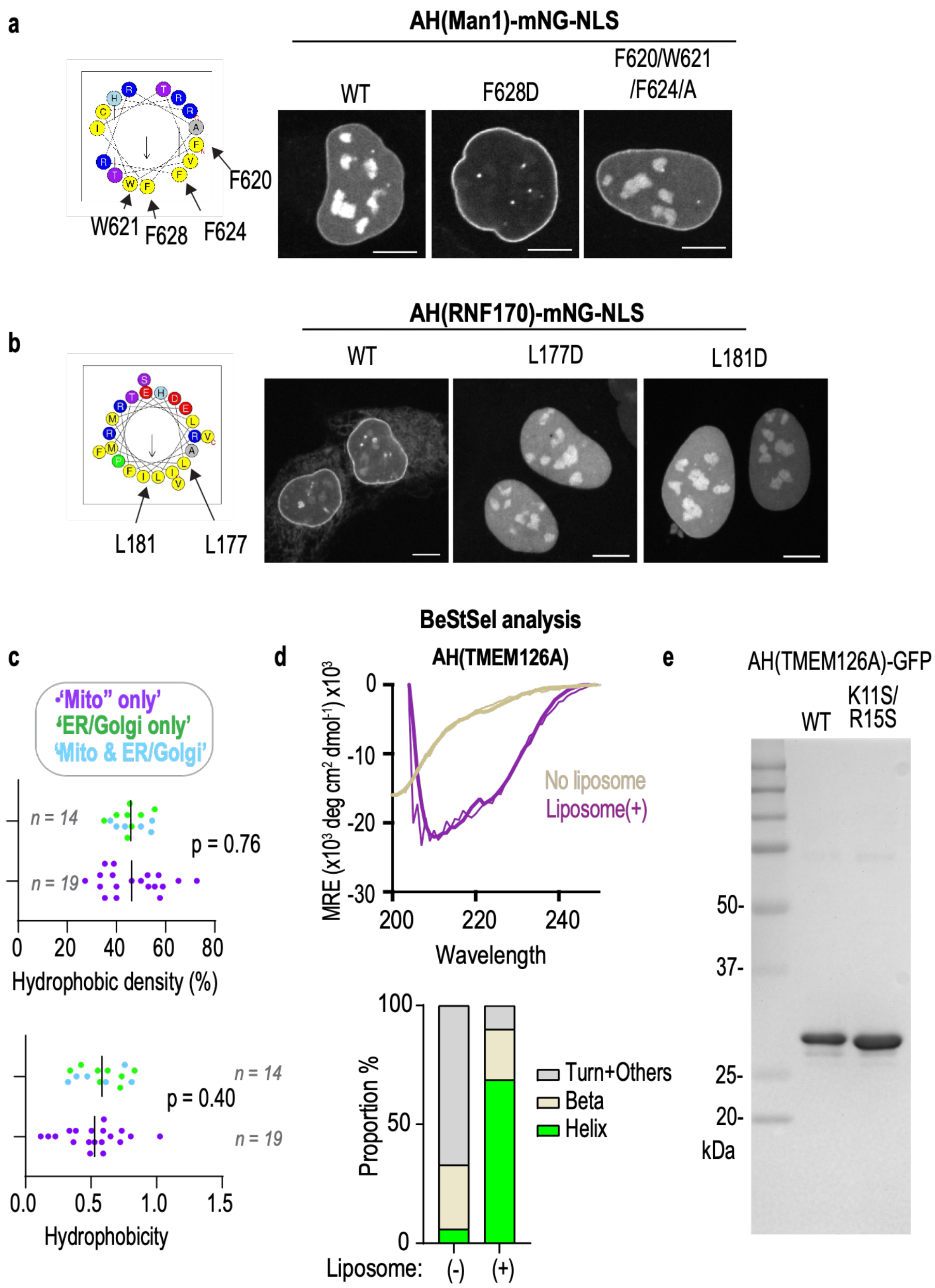
a
AH(Man1)-mNG-NLS
WT
F628D
F620/W621
/F624/A
F620
W621
F628
F624
b
AH(RNF170)-mNG-NLS
WT
L177D
L181D
L181
L177
c
'Mito" only'
'ER/Golgi only'
'Mito & ER/Golgi'
n = 14
n = 19
p = 0.76
Hydrophobic density (%)
p = 0.40
Hydrophobicity
BeStSel analysis
d
AH(TMEM126A)
MRE (x10³ deg cm² dmol⁻¹) x10³
Wavelength
No liposome
Liposome(+)
Proportion %
Turn+Others
Beta
Helix
Liposome:
(-)
(+)
e
AH(TMEM126A)-GFP
WT
K11S/
R15S
50-
37-
25-
20-
kDa

Fig. S9. Mutation analysis of nuclear-directed AH candidates Man1 and RNF170, AH properties analysis, and structural analysis of TMEM126.

a, b. Representative spinning disk confocal images of a single section in living U2OS cells expressing the indicated AH-mNG-NLS are shown. Experiments were conducted two times with similar results. Scale bars, 10 µm. **c.** Scatter plot of the indicated metrices of AH candidates categorized by their localizations as determined from experiments represented in Fig. 1B. **d.** Fitted curve and secondary structure estimate by BeStSel for representative CD spectra of AH(TMEM126A) peptide. Experiments were conducted two times with similar results. **e**. Coomassie stain of recombinant proteins as indicated for GUV experiments in Fig. 3 (only WT protein was used because mutant was unable to solubilize for CD analysis). Source data and unprocessed Coomassie stain image are provided as a Source Data file.

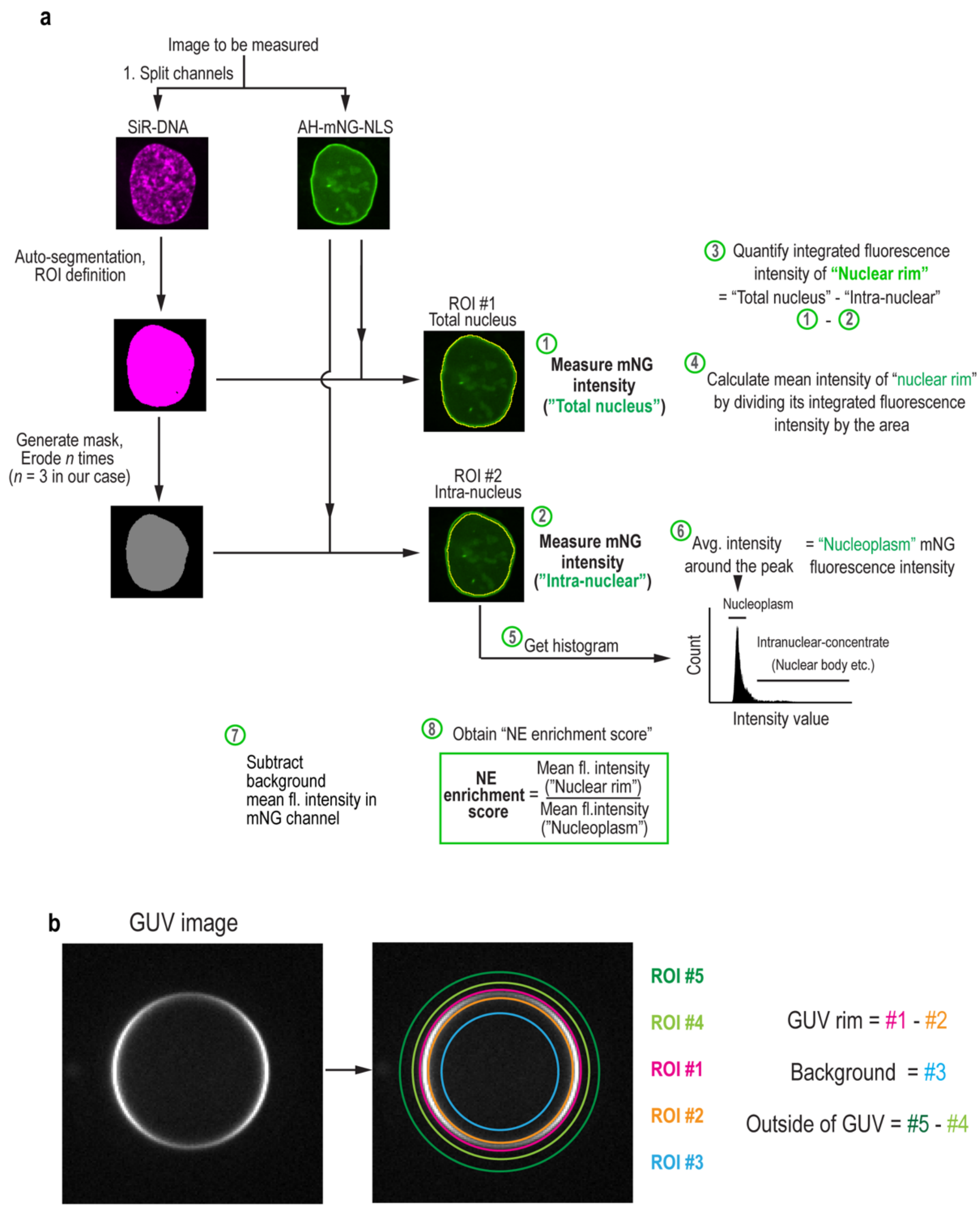

Fig. S10. Flow chart of the NE enrichment score (NEES) quantification and schematic of ROIs in AH-GUV binding quantification. a. Flow chart of the NE enrichment score quantification. **b.** Schematic illustration of ROIs in AH-GUV binding quantification.

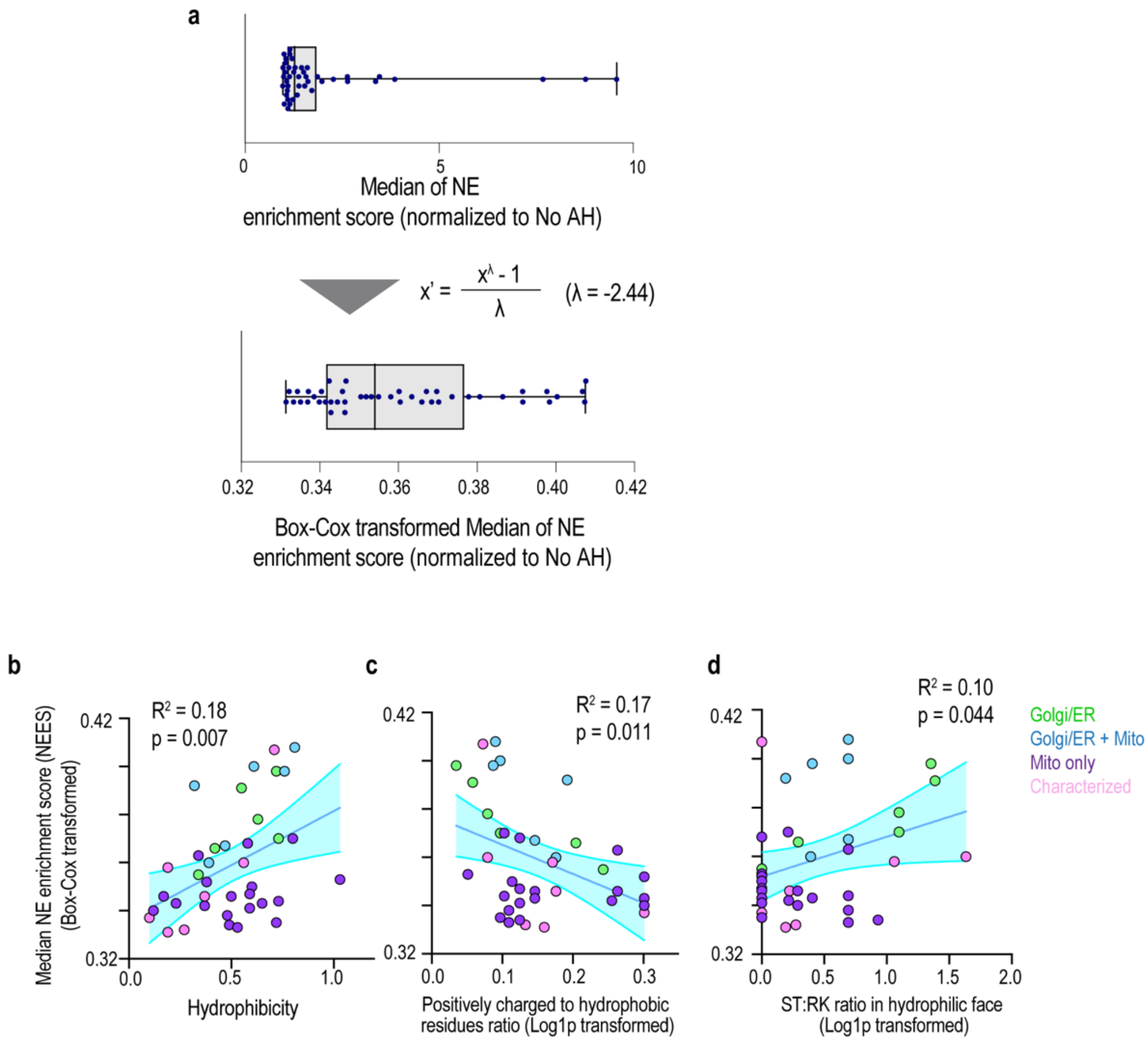

Fig. S11. Correlation analysis of AHs.

a. Median values of NE enrichment scores before and after Box-Cox transformation. **b-d.** Plot of linear correlation analysis of Median NE enrichment score (Box-Cox transformed) and the indicated properties for all nuclear-targeted AHs in Fig. 4C. P values and R^2 values are obtained by a linear regression analysis, and error bands indicate 95% confidence intervals. See Table S16 for the AH sequences and data values. Source data are provided as a Source Data file.

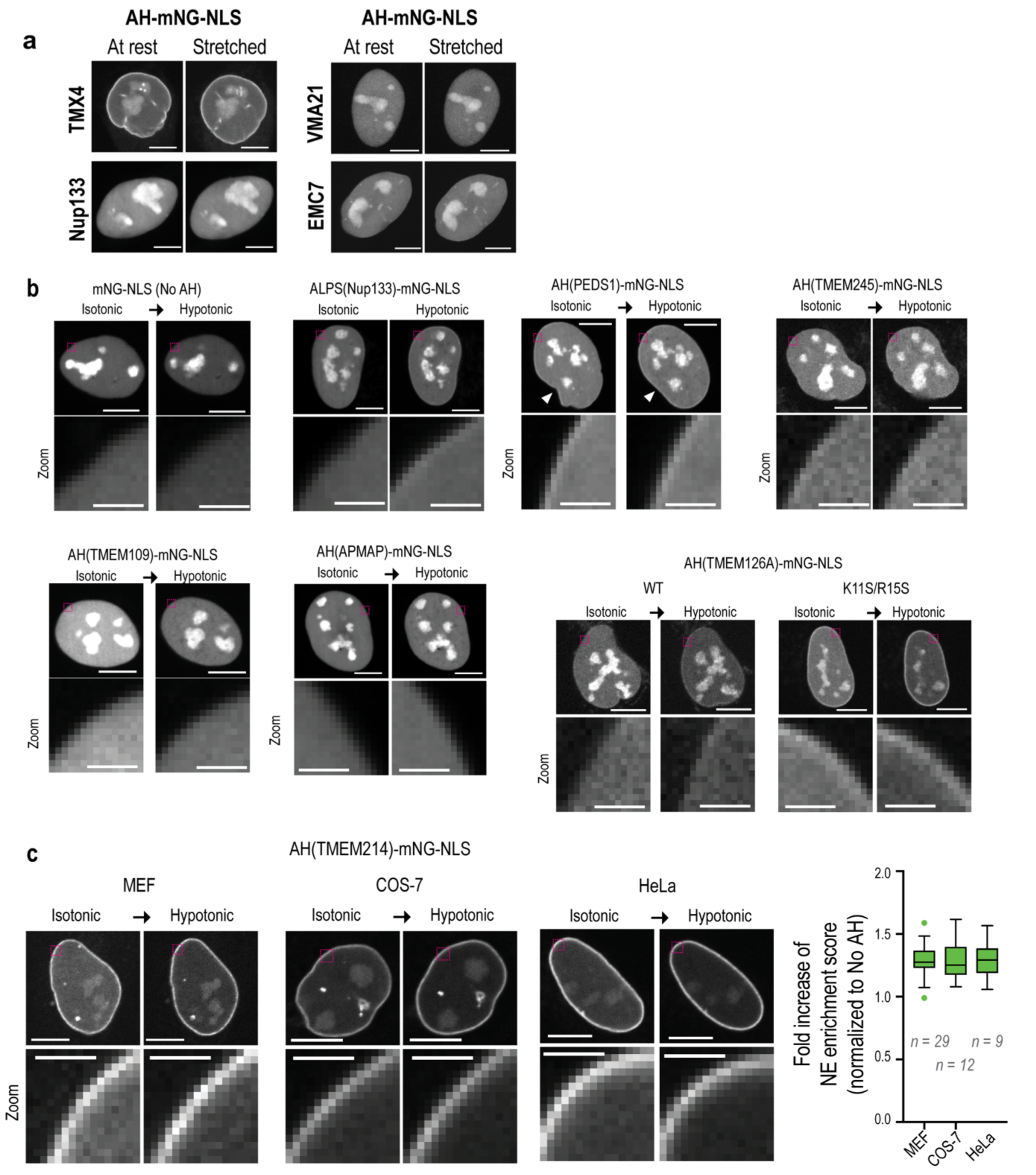

Fig. S12. Hypotonic shock, but not cell stretch, promotes AH binding to the inner nuclear membrane.

a. Spinning disk confocal images of single z-section of living HeLa cells expressing indicated AH-mNG-NLS construct before (at rest) and during cell-substrate stretch (stretched). **b.** Single z-section spinning disk confocal images of living U2OS cells expressing with the indicated AH-mNG-NLS constructs before and after the hypotonic shock. An arrowhead indicates the deformation of the nuclear rim wrinkle that is no longer present after hypotonic shock. Scale bars, 10 µm or 2 µm (zoom). **c**. Single z-section spinning disk confocal images of living MEF, HeLa and COS-7 cells with indicated AH-mNG-NLS before and after hypotonic shock. Box plot representing calculated fold increase in NE enrichment scores of indicated AH-mNG-NLS before and after hypotonic shock. Data are pooled from two independent experiments. box plots are shown by the Tukey method (the middle line: median; box: 25th and 75th percentiles; whiskers: the largest/smallest values or 25th/75th percentiles -/+ 1.5IQR; dots: outliers). Scale bars, 10 µm or 2 µm (magnified images). Source data are provided as a Source Data file.

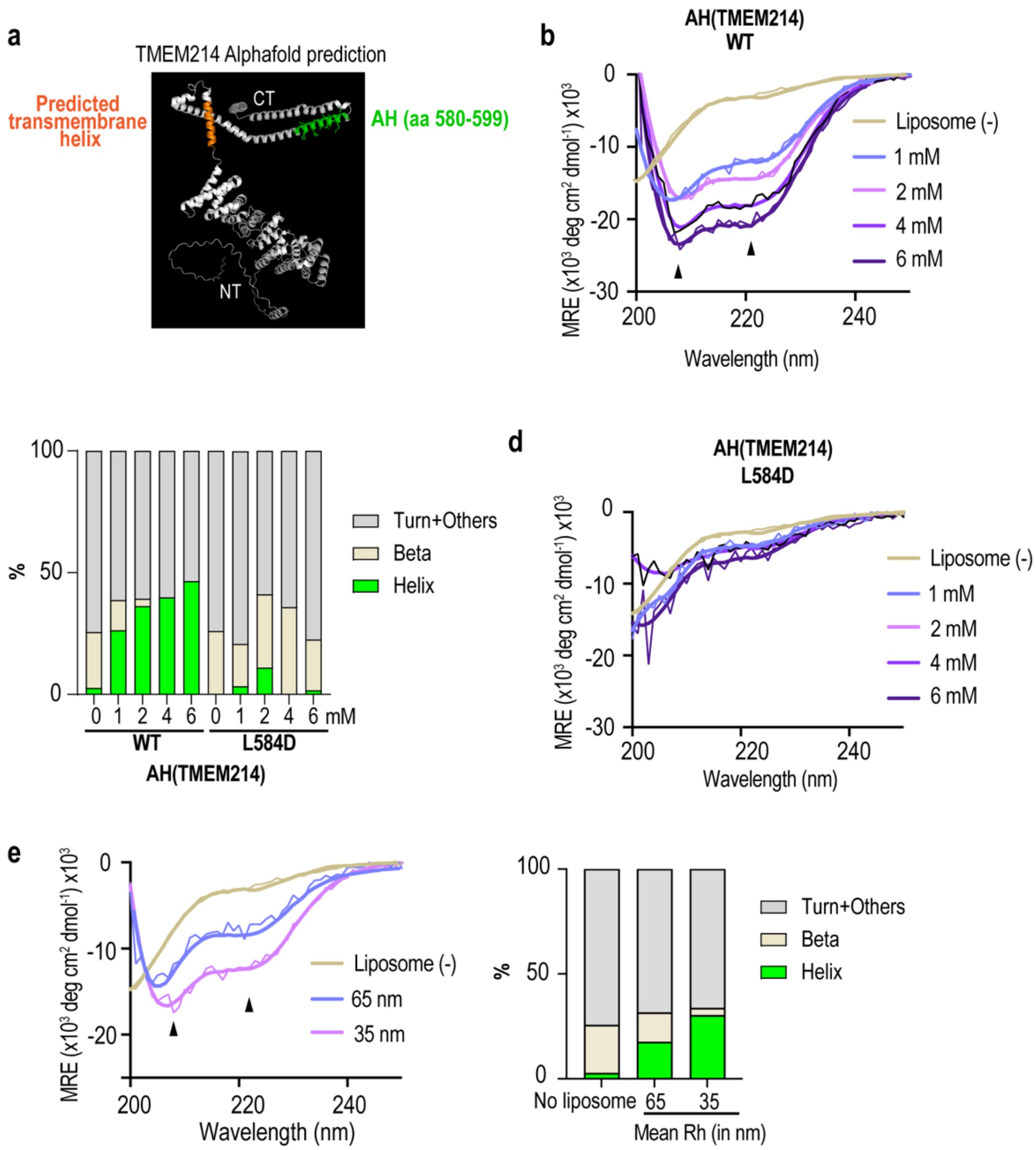

Fig. S13. Structural analysis of AH(TMEM214).

a. The putative AH is highlighted in green in predicted 3D structure of TMEM214 protein, (AlphaFold2 prediction presented by PyMol). A putative transmembrane (TM) domain predicted by TMHMM is shown in orange. The N and C termini are labeled (ID: Q6NUQ4). **b-e.** Fitted curve and secondary structure estimate by BeStSel for the representative CD spectra of the AH(TMEM214) peptides. Experiments were conducted two times with similar results.

Arrowheads indicate apparent local minima at 208 and 222 nm. Source data are provided as a Source Data file.

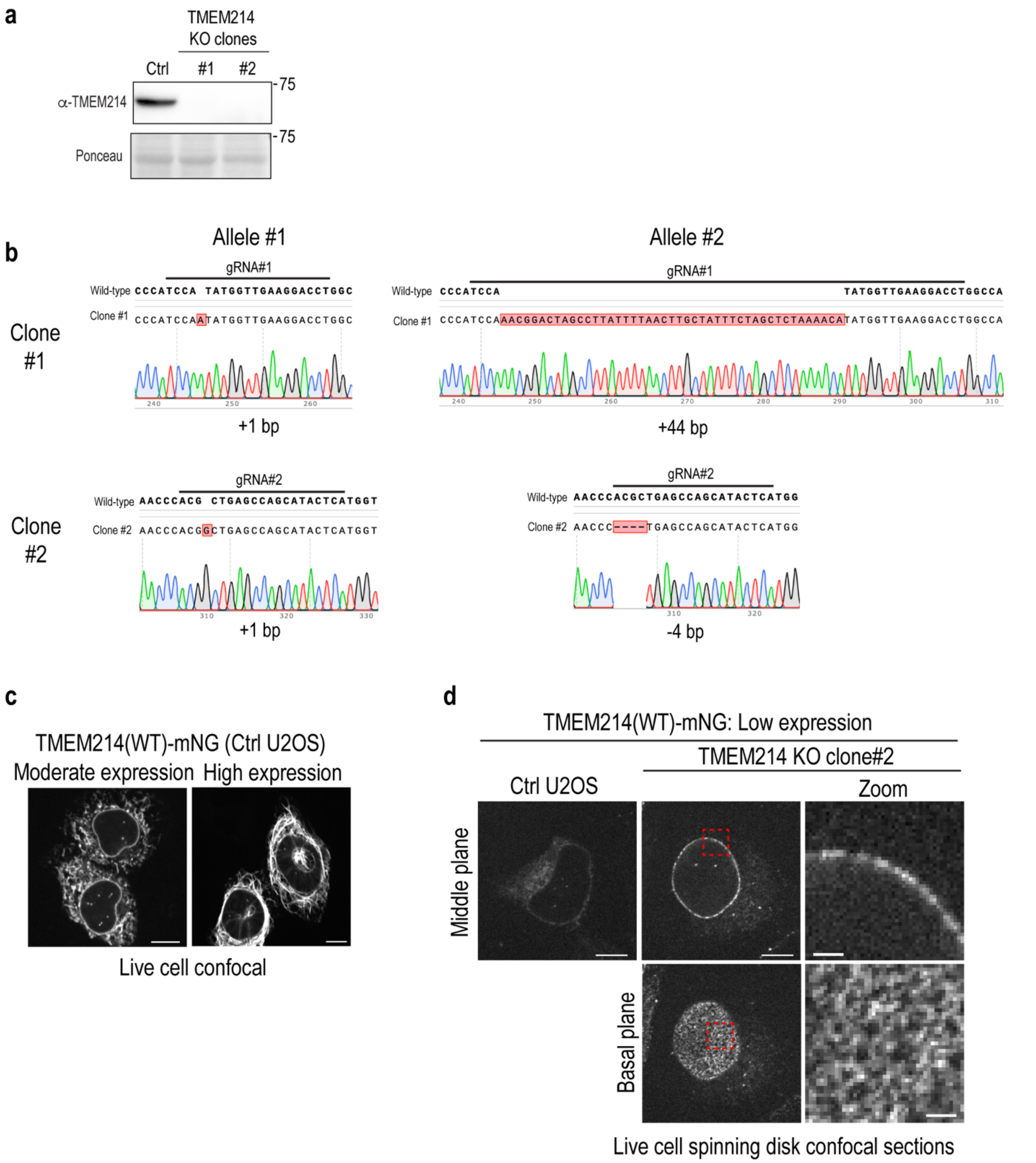

Fig. S14. Validation of TMEM214 KO U2OS cell lines and localization of exogenously expressed TMEM214 at different levels.

a. Representative immunoblot of cell lysates from control and TMEM214 KO U2OS cells. Experiments were conducted two times with similar results. **b.** Sequencing analysis of the genomic

regions that the sgRNA targets from the TMEM214 KO cell lines. **c-d.** Representative spinning disk confocal images of a single section in living control or TMEM214 KO (clone #2) U2OS cells expressing TMEM214-mNG are shown. Experiments were conducted two times with similar results.

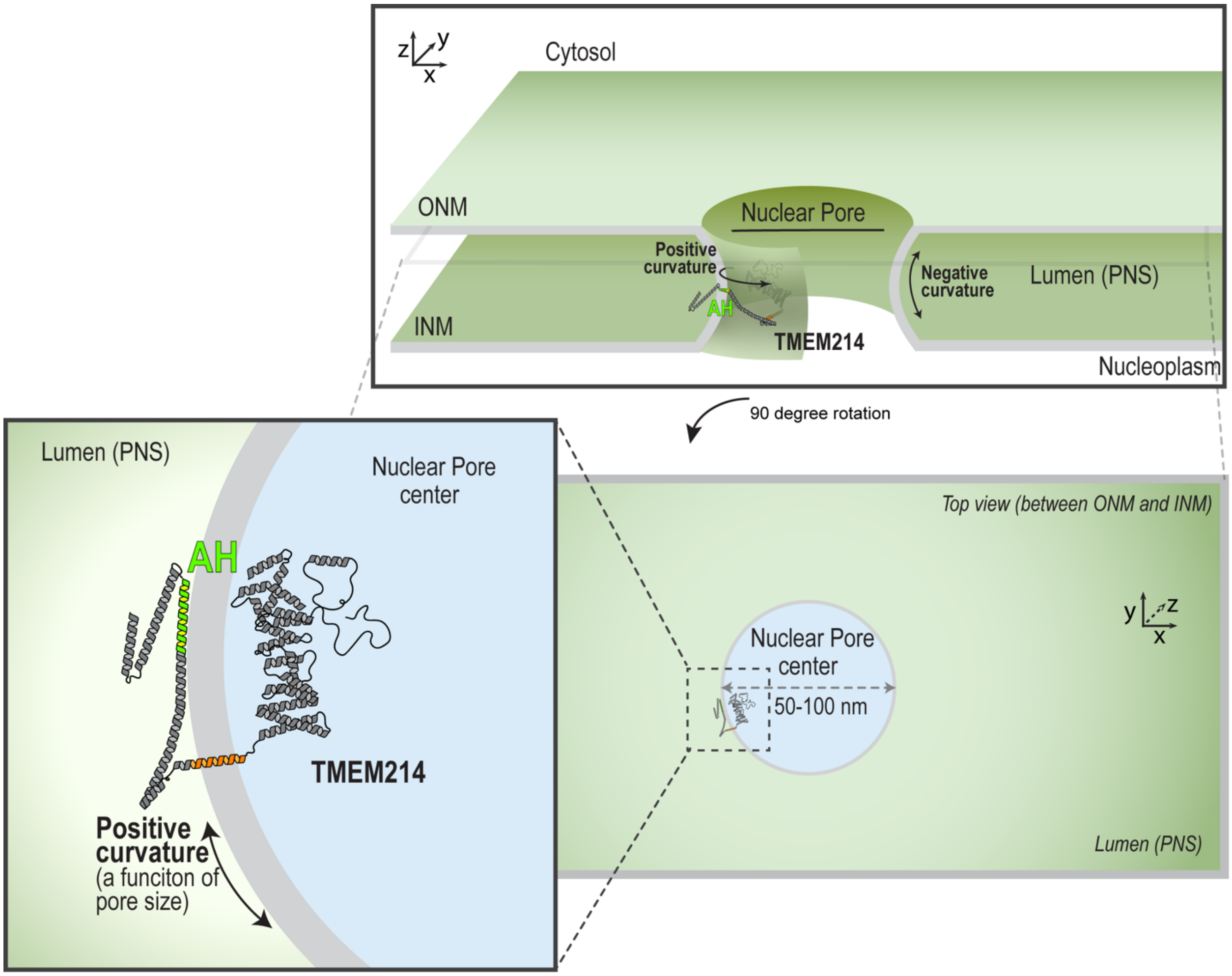

Fig. S15. Orientation of TMEM214 in the nuclear pore membrane relative to membrane curvature. Schematic representation of **t**he orientation of TMEM214 in the nuclear pore membrane based on predicted single pass transmembrane domain. Side view (top), top view of central cross section rotated 90 degrees, and zoom are shown. The nuclear pore membrane is similar to a saddle structure with both positive and negative curvatures, depending on the axis. Moving from the outer nuclear membrane (ONM) to the inner nuclear membrane (INM), the curvature on the lumen side is negative. In contrast, the curvature is positive around the pore circumference on the luminal side, and its value is a function of the pore size[2,3]. The predicted

orientation of TMEM214 is shown placing its AH facing the lumen of the nuclear pore, which may associate with the positively curved membrane.

Supplemental Figures References

1 Drin, G. *et al.* A general amphipathic alpha-helical motif for sensing membrane curvature. *Nat Struct Mol Biol* **14**, 138–146 (2007). https://doi.org/10.1038/nsmb1194

2 Zimmerli, C. E. *et al.* Nuclear pores dilate and constrict in cellulo. *Science* **374**, eabd9776 (2021). https://doi.org/10.1126/science.abd9776

3 Hoffmann, P. C. *et al.* Nuclear pore permeability and fluid flow are modulated by its dilation state. *Mol Cell* **85**, 537–554.e511 (2025). https://doi.org/10.1016/j.molcel.2024.11.038
