# Supplementary material for: Screening of amphipathic helices identifies features linked to inner nuclear membrane properties
Source: Nat Commun. 2026 Jul 30;17:9316. doi: 10.1038/s41467-026-75561-0 (PMC13530154; doi:10.1038/s41467-026-75561-0)
Supplement: Supplementary file 2 [file 41467_2026_75561_MOESM2_ESM.pdf]

nature portfolio

Peer Review File

Screening of amphipathic helices identifies features linked to inner nuclear membrane properties

Corresponding Author: Professor Shirin Bahmanyar

This manuscript has been previously reviewed at another journal. This document only contains information relating to versions considered at Nature Communications.

This file contains all reviewer reports in order by version, followed by all author rebuttals in order by version.

Version 1:

Reviewer comments:

Reviewer #1

(Remarks to the Author)
The authors have convincingly addressed all the points raised. I acknowledge that CD spectra obtained in the presence of liposomes are more difficult to analyze, and I appreciate the additional effort the authors made in this regard. I consider this work to be conceptually important for the field and therefore recommend it for publication.

(Remarks on code availability)

Reviewer #2

(Remarks to the Author)
By more carefully analyzing how the NE enrichment score relates to various features of AHs—particularly through a Partial Least Squares approach—the authors provide more refined conclusions about why certain AHs bind more strongly to the inner nuclear membrane. This addresses my final major criticism from the second round of revision for NSMB. I now recommend the publication of this work in Nature Communications.

Minor comments
1. The following sentence is unclear: "Neither AH(TMEM126) peptide bound to DOPC-only GUVs, as expected from the lack of electrostatic interactions or packing defects. However, both peptides bound strongly to GUVs containing both negatively charged lipids (DOPS) and conical lipids (dioleoyl-glycerol (DOG), a DAG analogue known to induce packing defects in model membranes (Čopič et al., 2018; Garten et al., 2015)) (Fig. 3D–E)."
2. Regarding Fig. S14E: Perhaps my previous request was unclear, but I would like to see a schematic illustrating the positions of the N- and C-terminal regions of TMEM124 relative to the nuclear membrane. Figure S14E does not currently show the protein.

(Remarks on code availability)

Open Access This Peer Review File is licensed under a Creative Commons Attribution 4.0 International License, which permits use, sharing, adaptation, distribution and reproduction in any medium or format, as long as you give appropriate credit to the original author(s) and the source, provide a link to the Creative Commons license, and indicate if changes were made.
In cases where reviewers are anonymous, credit should be given to 'Anonymous Referee' and the source.
The images or other third party material in this Peer Review File are included in the article's Creative Commons license, unless indicated otherwise in a credit line to the material. If material is not included in the article's Creative Commons license and your intended use is not permitted by statutory regulation or exceeds the permitted use, you will need to obtain permission directly from the copyright holder.
To view a copy of this license, visit https://creativecommons.org/licenses/by/4.0/

Response to Reviewers (2nd round of comments from NSMB)

We thank the Reviewers for the thorough evaluation of our work and for their suggestions. We addressed their concerns to the best of our ability, as outlined below. In addition to summarizing and directly quoting changes in the manuscript pertaining to their suggestions below, we provided a Word document with track changes.

Reviewer #1 (Remarks to the Author):

In the revised manuscript titled "Systematic Screening of Amphipathic Helices Reveals Inner Nuclear Membrane Properties," Lee et al. aim to establish rules governing the peripheral association of proteins with the inner nuclear membrane through amphipathic helices (AHs). In their rebuttal letter, they emphasize that data and analyses for a total of 44 AHs have now been included. However, this applies solely to localization studies involving fusions of the AHs with a fluorescent marker (with and without a nuclear localization signal), which ultimately lead to a nuclear envelope enrichment score.

Reviewer 1.1: *What remains lacking is a comprehensive analysis demonstrating whether these AHs can indeed bind to membranes; specifically, the giant unilamellar vesicle (GUV) assay has only been performed for the TMEM126 AH and its mutants. Similarly, circular dichroism (CD) spectroscopy has been conducted exclusively on TMEM126 and TMEM214 AHs.*

Response to Reviewer 1.1:

We have substantially rephrased the manuscript to clarify the scope of our conclusions and to make explicit which statements are directly supported by experimental data versus inferred from amphipathic helix (AH) behavior. Key changes and their locations are summarized below.

We revised the **Title** to read "*Screening of amphipathic helices identifies features linked to inner nuclear membrane properties.*" Removing the word "Systematic" and replacing the verb "reveals" with "identifies features linked to" avoids definitive statements about INM properties and to reflect inference based on AH behavior.

We revised the **Abstract (revised manuscript p. 2)** in several ways to rephrase the scope of the study and inferences that were made on INM properties, rather than implying universal applicability. The changes include but are not limited to:

1. We specify AH(TMEM126) as a single AH for which we performed *in vitro* GUV binding studies and mutational analysis and remove any over-generalization of the findings from this AH to all other AHs.

   "Consistent with this relationship, mutational analysis of a mitochondrial AH from TMEM126 altered its lipid-binding preference toward membranes containing lipid packing defects in vitro, shifted its localization toward ER/Golgi membranes, and enabled INM association upon nuclear targeting."

2. We replaced words such as "Defines" and "reveals" with "identify" and "suggest" to clearly distinguish experimental results from inferred properties.

   "Together, we identify features of AHs associated with INM binding that suggest corresponding biophysical properties of the INM, with relevance to nuclear mechanical responses."

We revised the **Introduction–Results** transition to provide clarity on which AHs were studied:

Revised manuscript p. 5, **lines 149–162**: *"We screened amphipathic helices (AHs) from putative nuclear envelope (NE) and ER associated proteins to relate amino acid sequence features to cytosolic organelle localization…."*

Immediately following this sentence, we specify the experimental context (isolated AHs, nuclear targeting, image-based screening) for the specific number of AHs we studied. We describe that *in vitro* binding and mutagenesis results were for a single AH(TMEM126) to make the scope explicit.

In the **Results** section, we made similar word replacements and removals and rephrased sentences to ensure clarity on the direct outcomes of our experimental strategies, rather than over-arching claims on our findings.

Pg. 6 (line 217): We removed the word "systematic" before screening.

pg. 9 (line 289): We replaced *"defining INM properties"* with "*inferring INM properties."*

pg. 11: We changed the concluding paragraph on analysis of AH(TMEM126) by replacing *"providing an important tool to help interpret our studies of INM association of nuclear-targeted AHs in cells"* to **(lines 353-357)** *"provides a framework for interpreting the features that promote its association with the INM when targeted to the nucleus (see below)"* thereby minimizing over-generalization and emphasizing the experimental results from this AH.

pg. 11, lines 363-365: We were more clear that our motivations was to make inferences from our correlative, imaged-based analysis: **"***We analyzed INM enrichment for each AH candidate and compared them to the characterized AHs to infer features important for INM-AH binding as well as corresponding INM properties (Fig. 4A-C)."*

pg. 12, lines 389-391, we are clear that the data are supportive of the interpretation for the subset of AHs that we analyzed: *"These data support the interpretation that AHs associating with the INM share features with ER/Golgi-localized, rather than mitochondrial, AHs, and that electrostatic interactions are not the dominant contributor for their INM association."*

pg. 13, lines 420-421, similar to the point above, these lines now read *"These data further supported that electrostatics is not the major contributor to INM association for a subset of AHs in this study…."*

pg. 13, lines 434-435: we remove any suggestion of definitive claims by writing *"consistent with the possibility that the INM has a relatively low-charge surface that can concentrate weak AHs and facilitate subsequent membrane binding."*

Pg. 14, lines 491-494, now reads *"Nevertheless, these correlation analyses support the interpretation that, for the subset of AHs examined, the hydrophobic effect contributes more strongly than electrostatics to INM association leading us to infer INM contains lipid packing defects with a low surface charge."*

6. Discussion:

By insertion of "*a subset of AHs*" and replacement of "*revealing*" with "*suggesting,*" as well as other wording changes specified below we explicitly limit the scope of conclusions and remove overgeneralization in our Discussion. We also explicitly state that the inferences on INM properties are a model, rather than a definitive claim.

p. 18, Lines 630-633 now reads: *"Collectively, we provide evidence for a subset of AHs that bind the INM through this mechanism, suggesting features of the INM permissive for such interactions…."*

p. 18, lines 636-639, we more explicitly clarify our experiments on specific AHs, and our image-based approach to avoid over-generalization: ***"****Our* <u>*mutagenesis experiments on AH(TMEM126) as well as our computational and correlation analyses from image-based screening of candidate and well-characterized AHs*</u> *further supports* <u>*our model*</u> *that sensitivity to lipid packing defects with a minor contribution from electrostatics promotes AH-INM binding."*

Pg. 19, lines 666-668, we write: *"further supporting* <u>*our model*</u> *that lipid packing defects are a property of the INM…."*

In the last line of the Discussion on **pg. 21**, we more explicitly state what we did and how this rationalizes our interpretations and model. **Lines 731-735** now reads *"Our work examining features correlated with INM-association of a subset of AHs, as well as mutatgenesis and in vitro studies on AH(TMEM126), suggests physicochemical features promoting amphipathic helix-INM interactions and provides a framework for understanding the properties of the INM in mammalian cells, an important area to consider in both healthy conditions and in diseases associated with the NE."*

In summary, the revised manuscript clarifies experimental scope, removes over-generalization, and clearly distinguishes measured AH behavior from inferred INM features, directly addressing the reviewer.

Reviewer 1.2: *I find it challenging to interpret the CD data as exhibiting a clear α-helical pattern, given that it lacks the characteristic minima at 208 nm and 222 nm typically associated with α-helices. Consequently, I remain unconvinced by these novel data, and the overall conclusions regarding the binding requirements of amphipathic helices to the inner nuclear membrane are not well substantiated.*

Response to Reviewer 1.2: We do not agree with the Reviewer on this point.

First, we consulted Dr. Guillaume Drin (CNRS), an expert in amphipathic helix membrane binding properties and circular dichroism (see (Drin et al., 2007; Drin et al., 2001a; Drin et al., 2001b)), as acknowledged in our manuscript (see **Acknowledgements**) and followed his advice on the protocol to minimize noise. We used (i) a cuvette with the shortest pathlength (0.05 cm) among those commercially available; (ii) fluoride instead of chloride in buffer (the latter of which abso2rbs a significant amount of UV); (iii) an average of 10 or more scans; and we avoided UV-absorbing solvent such as DMSO when solubilizing peptides (see **Materials and Methods**).

Second, while we are confident that our CD spectral analysis is of good quality, we further addressed the Reviewer's comment by applying the analysis software BeStSel (Micsonai et al., 2015)(cited by >1500) to our CD spectra. BeStSel provides an unbiased and objective estimate of the proportions of secondary structures considering the CD spectral pattern (note that in **Fig. 7C of the prior version of the manuscript**, we had only estimated the helical content solely from the MRE value at 222 nm, as stated in the **Materials and Methods**). This new analysis estimated the helicity of the AH(TMEM126A) (see **new panel in Fig. S9D**) and AH(TMEM214) (see **new panel in Fig. S13**) for all conditions in which we performed CD spectroscopy (except for **previous Fig. S9C**, which we removed in response to **Reviewer concern 1.4** – see below). We include the fitted curve generated by BeStSel to highlight the presence of local minimum at around 222 nm as well as at 208 nm (**see Fig. S9D** and **Fig. S13B, C**).

Finally, we believe our CD spectra (see **Fig. 3B and Fig. 7B and 7D**) is comparable to those published. We provide examples of Fig. 2A from (Hofbauer et al., 2018), Fig. 6B from (Daste et al., 2018), and Figs. 8 and 9 from (Hoshino and Sakane, 2020), where the CD spectroscopy and analysis helped establish the presence and biological significance of membrane binding amphipathic helices.

Fig. 2A of (Hofbauer et al., 2018)

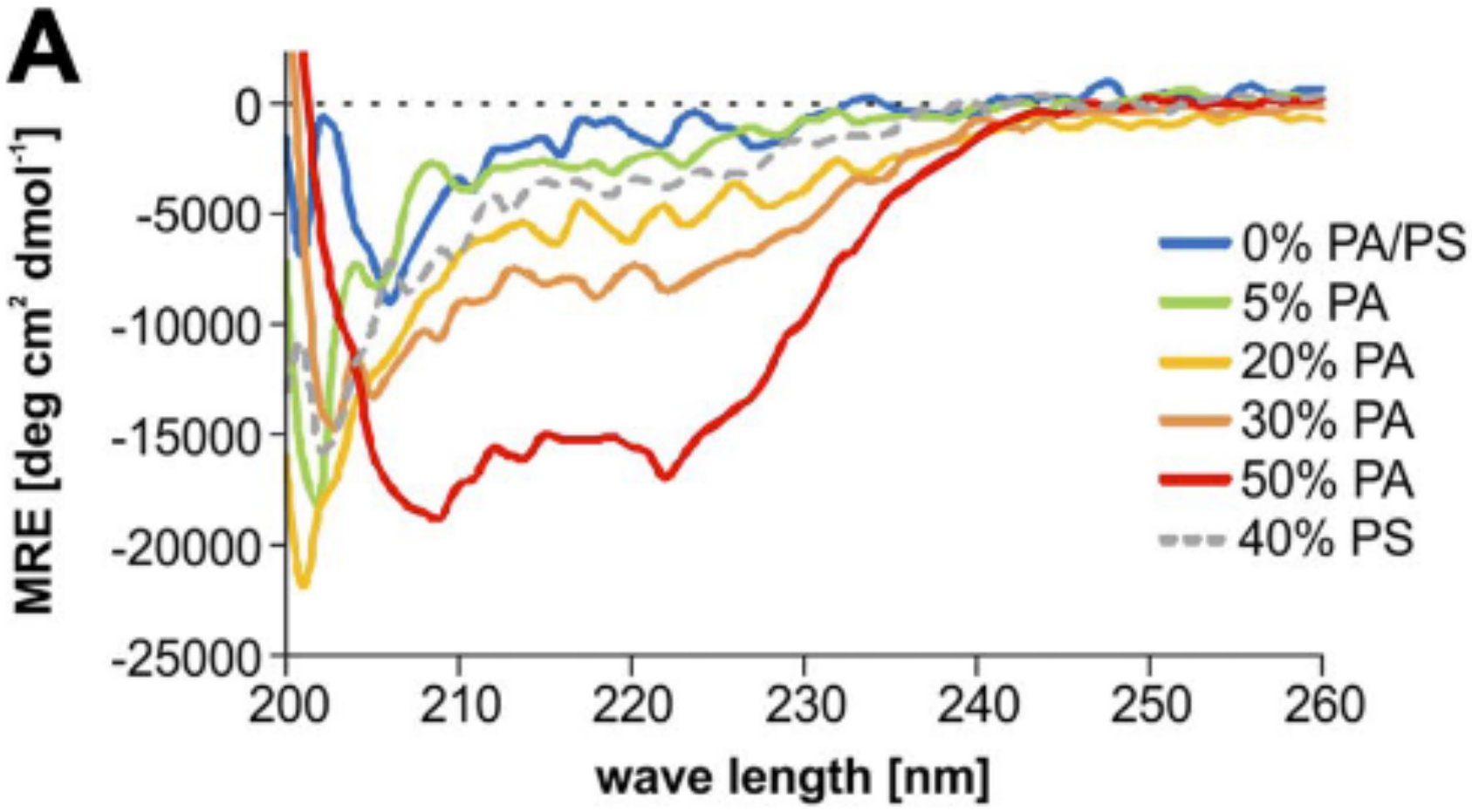

Fig. 6B of (Daste et al., 2018)

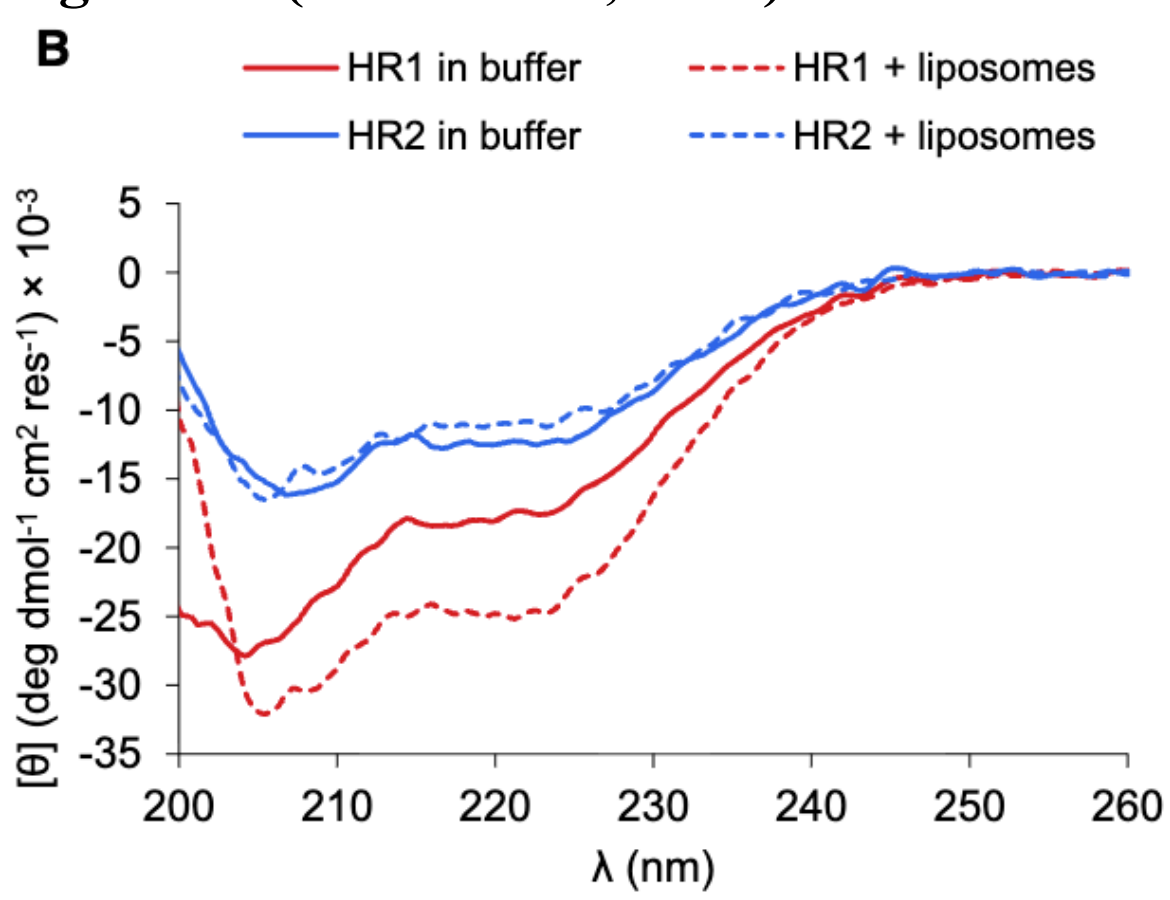

Fig. 9 of (Hoshino and Sakane, 2020)

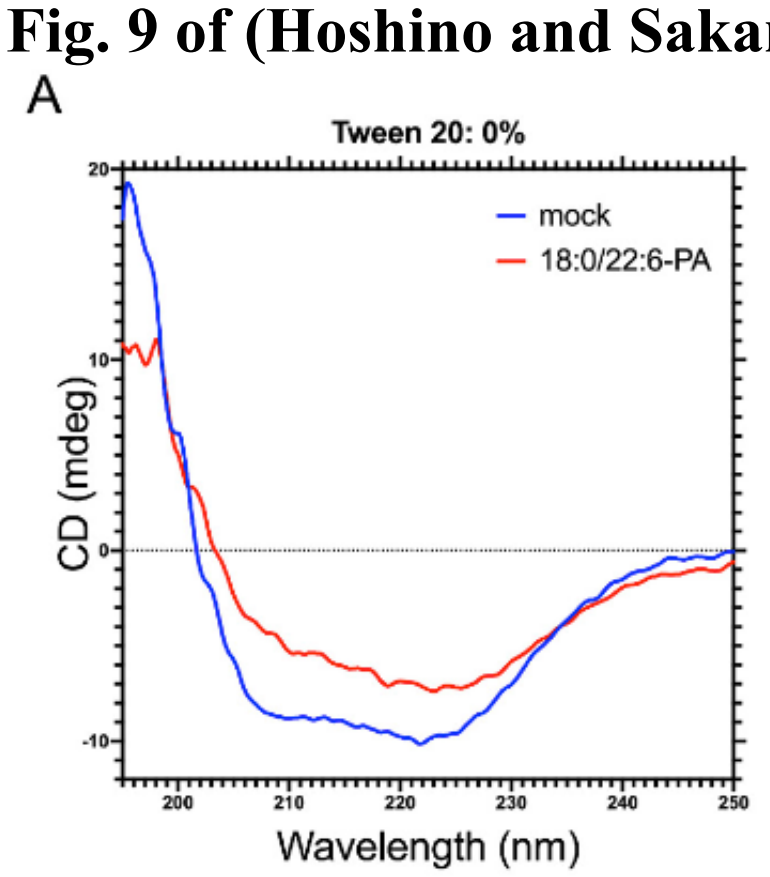

Reviewer 1.3: *Additionally, the work includes data indicating that TMEM214 is associated with the nuclear pore complex. While this finding is intriguing, its relevance remains unclear.*

Response to Reviewer 1.3: We used a screening approach to identify membrane proteins that may be nuclear envelope-associated and to predict sequences that are amphipathic helices in these candidate proteins. Inherently, screens may not be successful in addressing the original goal. The observation that TMEM214 contains an AH and is nuclear envelope-associated demonstrates that our screening approach was successful. We modified the text in the **Abstract** and **Discussion** to clarify that our analysis of the AH of TMEM214 and the localization of TMEM214 was important for (**Intro, lines 131-132**) *"demonstrating the utility of this screening approach*

for uncovering lipid-binding AHs at the nuclear envelope" and **(Abstract, lines 36-37)** *"providing independent validation of the screening approach."* Please note that this was a request in response to a comment from Reviewer 2 in the previous submission.

Specific Points:
Reviewer 1.4: *1. I was surprised by the use of 2,2,2-trifluoroethanol (TFE) for dissolving some of the TMEM126 peptides. TFE is commonly employed to stabilize and promote α-helix formation in peptides and proteins in solution; in practice, adding TFE often increases helical content observed via CD, NMR, or other structural probes. If TFE is utilized in this study, what conclusions can be drawn from these measurements? It should also be noted that even with TFE present, the CD spectrum does not exhibit a definitive profile indicative of α-helices.*

Response to Reviewer 1.4: This was not a major point, so we removed the CD spectrum where we used TFE from our **previous Fig. S9C**, as well as the associated text.

Reviewer 1.5: *2. In Supplementary Figure 9: Why does the Man1 F628D mutant display nuclear envelope localization? This result contradicts expectations as it should disrupt the hydrophobic surface of the amphipathic helix.*

Response to Reviewer 1.5: Yes, exactly - the finding that introducing a charged amino acid in the hydrophobic face does not disrupt AH(Man1) localization indicates that its localization is facilitated by a mechanism other than direct peripheral membrane binding, such as protein-protein association or covalent lipid modification. This predicted helix was the only one from the 56 that we screened that had the atypical localization to the plasma membrane when expressed in isolation. We eliminated it our follow up analysis because the mutation showed that the amphipathicity of the amino acid sequence was not important for its membrane localization. We modified the text (see **Main text, Results pg. 7, lines 248 to 254**) to make this more clear: *"Note that only one AH (AH(Man1)) localized to the plasma membrane, but replacing a hydrophobic residue with a charged residue on the hydrophobic face did not alter its membrane association when targeted to the nucleus, while the same mutation for a different AH (AH(RNF160)) did, indicating that the amphipathic nature of the predicted AH of Man1 does not facilitate membrane binding (see Fig. S9A, B) (Drin and Antonny, 2010). We therefore eliminated this predicted AH from our analyses because other mechanisms (such as protein-protein interactions or covalent lipid modifications) are likely involved in its plasma membrane localization."*

Reviewer 1.6: *3. TMEM214 has been identified in a proteomic screen as an interactor with several nuclear pore complex proteins (PMID: 26496610). It may be worthwhile to cite this work for further context.*

We added this to **Main Text, Results pg. 17.**

Reviewer #2 (Remarks to the Author):

Reviewer 2.1: *The authors have substantially improved the quality of their study and manuscript by:*
1. analyzing a larger set of amphipathic helices for their ability to bind the inner nuclear membrane (INM),
2. enhancing their statistical analysis,
3. providing structural insights using circular dichroism on amphipathic helices identified in the proteins TMEM126A and TMEM214, and
4. being more cautious about certain aspects regarding the identification of amphipathic helices in proteins.
Overall, the manuscript is now much more readable and convincing.

Response to Reviewer 2.1: We thank the Reviewer for the helpful and constructive feedback.

Reviewer 2.2: *However, I would like to provide what I hope is constructive criticism, which the authors may wish to address before potential acceptance by Nature Structural & Molecular Biology (NSMB).*

An analysis combining the frequency of amino acids in the polar and hydrophobic faces of amphipathic helices with Linear Discriminant Analysis (LDA) elegantly distinguishes helices that bind exclusively to mitochondria from those that bind the ER/Golgi or ER/Golgi/mitochondria (Fig. 2B, C, D). Meanwhile, the ST/KR ratio is not effective in separating the "ER/Golgi" and "ER/Golgi/mito" groups (Fig. 2E).
In Figure 4C, the amphipathic helices are again divided into "ER/Golgi" and "ER/Golgi/mito" groups, even though the ST/KR ratio cannot reliably distinguish them. Moreover, it is the helices from the "ER/Golgi/mito" group that bind most effectively to the INM. In other words, the helices with the highest ST/KR ratio are not the ones that bind best to the INM (if this were the case, the ALPS motif should be among the best binders, which it is not, as shown in Fig. 4C).
Faced with these results, the authors seem determined to find simple relationships between a single variable (e.g., hydrophobicity) and the INM binding index (NEES). While they identify statistically significant p-values, the low R^2 values suggest that additional explanatory parameters are involved. Why not accept that the NEES value depends on a combination of 2–3 parameters? Why not perform an LDA-type analysis to interpret the NEES values?

Ultimately, from what I understand, the authors conclude that amphipathic helices bind most effectively to the INM through a combination of low electrostatic interactions and a strong dependence on packing defects. These helices are hydrophobic, with a non-maximal ST/KR ratio. This suggests a bell-shaped relationship, which could explain why the "ER/Golgi/mito" group performs best. It would be valuable for the authors to explore this further and make their conclusions more straightforward.

Response to Reviewer 2.2: We agree with the Reviewer that AH association with the INM is governed by a combination of physicochemical properties or descriptors. In the revised manuscript, we edited the **Main text, Results**, **pp. 13-15** to more clearly reflect that the NEES likely depend on a combination of features, and not a single variable. We also now more explicitly state that the ST/RK ratio is not a reliable indicator for INM binding.

To address the Reviewers suggestion, we first attempted a non-linear fitting of the ST:RK ratio, which revealed a weak bell-shaped trend (**R^2 = 0.23, see Response to Reviewers Fig. 1**) with the highest NEES corresponding ER/Golgi+Mito-localized AHs that have relatively low to moderate S/T:R/K ratios, as the Reviewer pointed out. We decided not to include this analysis in the manuscript and instead focus on the combination of parameters that explain the NEES. Instead, we explicitly discuss that the S/T:R/K ratio is not a good indicator of INM binding (**Main text, Results, pp. 13-15**).

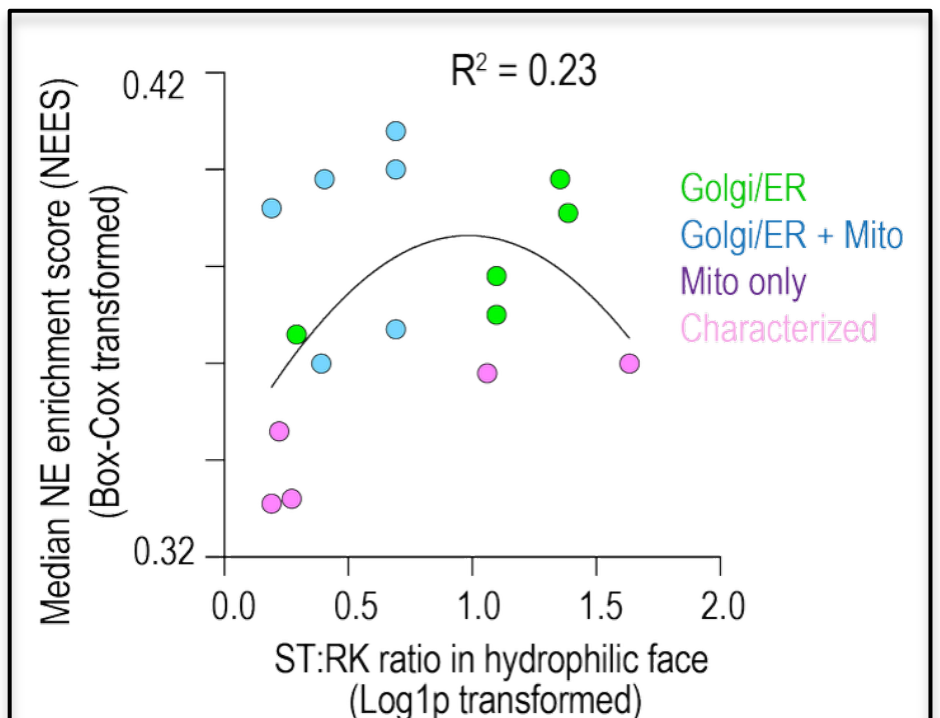

Response to Reviewers Fig. 1. Non-linear fitting for AH candidates indicated.

Second, we specifically state that a single variable alone does not fully account for the NEES variation (Results, **pg. 14)**. We then explicitly address the reviewer's point that NEES likely depends on a combination of parameters, by performing a Partial Least Squares (PLS) analysis (see **new Fig. 5B and Main text, Results, pp. 13-15**). In brief, because NEES is a continuous variable rather than a predefined class label, LDA is not an appropriate framework for interpreting NEES values. PLS is well-suited for this purpose because it identifies linear combinations of interrelated descriptors that covary with a continuous outcome, rather than treating each parameter independently. This is particularly appropriate here, as hydrophobicity, hydrophobic density, ST:RK ratio are inherently linked by amino-acid composition.

As stated in the text and shown in **Fig. 5B**, the PLS results support the conclusion that INM binding reflects a composite of physicochemical parameters in which the hydrophobic effect and sensitivity to membrane packing defects outweigh electrostatic attraction. This analysis helps explain why AHs with high hydrophobicity can bind effectively to the INM even in the presence of substantial charge, while AHs with relatively low hydrophobic density and low charge can also be permissive for INM binding. It also provides an unbiased approach to explain why the ST/KR ratio alone does not predict NEES values.

Minor remarks:
Reviewer 2.3: *I do not believe the Heliquest D criterion is useful for the analysis in Figure 2, as it appears to be a metric for determining whether a helix binds to a lipid membrane (and thus should be applied to explain why some amphipathic helices remain purely cytosolic).*

Response to Reviewer 2.3: The reviewer is right. D factor does not help distinguish AHs that bind different types of membrane by its definition where it predicts membrane-binding propensity. We removed D factor from **Fig. 2 and 5**.

Reviewer 2.4: *The authors should include a schematic of the expected topology of the TMEM124 protein in the nuclear membrane to clarify their discussion.*

Response to Reviewer 2.4: Done. **See new Fig. S14E**

Reviewer 2.5: *Typo: Figure 2B: "Hydrophylic face" should read "hydrophobic face."?*

Response to Reviewer 2.4: Thank you for pointing this out. It has been corrected.

Reviewer #3 (Remarks to the Author): *I am happy with the revisions and now recommend publication.*

Daste, F., C. Sauvanet, A. Bavdek, J. Baye, F. Pierre, R. Le Borgne, C. David, M. Rojo, P. Fuchs, and D. Tareste. 2018. The heptad repeat domain 1 of Mitofusin has membrane destabilization function in mitochondrial fusion. *EMBO Rep*. 19.

Drin, G., and B. Antonny. 2010. Amphipathic helices and membrane curvature. *FEBS Lett*. 584:1840–1847.

Drin, G., J.F. Casella, R. Gautier, T. Boehmer, T.U. Schwartz, and B. Antonny. 2007. A general amphipathic alpha-helical motif for sensing membrane curvature. *Nat Struct Mol Biol*. 14:138–146.

Drin, G., H. Déméné, J. Temsamani, and R. Brasseur. 2001a. Translocation of the pAntp peptide and its amphipathic analogue AP-2AL. *Biochemistry*. 40:1824–1834.

Drin, G., M. Mazel, P. Clair, D. Mathieu, M. Kaczorek, and J. Temsamani. 2001b. Physico-chemical requirements for cellular uptake of pAntp peptide. Role of lipid-binding affinity. *Eur J Biochem*. 268:1304–1314.

Hofbauer, H.F., M. Gecht, S.C. Fischer, A. Seybert, A.S. Frangakis, E.H.K. Stelzer, R. Covino, G. Hummer, and R. Ernst. 2018. The molecular recognition of phosphatidic acid by an amphipathic helix in Opi1. *J Cell Biol*. 217:3109–3126.

Hoshino, F., and F. Sakane. 2020. Polyunsaturated fatty acid-containing phosphatidic acids selectively interact with L-lactate dehydrogenase A and induce its secondary structural change and inactivation. *Biochim Biophys Acta Mol Cell Biol Lipids*. 1865:158768.

Micsonai, A., F. Wien, L. Kernya, Y.H. Lee, Y. Goto, M. Réfrégiers, and J. Kardos. 2015. Accurate secondary structure prediction and fold recognition for circular dichroism spectroscopy. *Proc Natl Acad Sci U S A*. 112:E3095–3103.

Response to Reviewers

Reviewer #1 (Remarks to the Author):

The authors have convincingly addressed all the points raised. I acknowledge that CD spectra obtained in the presence of liposomes are more difficult to analyze, and I appreciate the additional effort the authors made in this regard. I consider this work to be conceptually important for the field and therefore recommend it for publication.

Reviewer #2 (Remarks to the Author):

By more carefully analyzing how the NE enrichment score relates to various features of AHs—particularly through a Partial Least Squares approach—the authors provide more refined conclusions about why certain AHs bind more strongly to the inner nuclear membrane. This addresses my final major criticism from the second round of revision for NSMB. I now recommend the publication of this work in Nature Communications.

Minor comments

1. The following sentence is unclear: "Neither AH(TMEM126) peptide bound to DOPC-only GUVs, as expected from the lack of electrostatic interactions or packing defects. However, both peptides bound strongly to GUVs containing both negatively charged lipids (DOPS) and conical lipids (dioleoyl-glycerol (DOG), a DAG analogue known to induce packing defects in model membranes (Čopič et al., 2018; Garten et al., 2015)) (Fig. 3D–E)."

<u>*Response to Reviewer 2#1:*</u> We now changed it to "Neither AH(TMEM126) peptide bound to DOPC-only GUVs, consistent with the absence of electrostatic interactions and lipid-packing defects. In contrast, both peptides bound strongly to GUVs containing negatively charged lipids (DOPS) and conical lipids (dioleoyl glycerol, DOG)—a DAG analogue known to induce packing defects in model membranes[62,63] (Fig. 3D–E)."

2. Regarding Fig. S14E: Perhaps my previous request was unclear, but I would like to see a schematic illustrating the positions of the N- and C-terminal regions of TMEM124 relative to the nuclear membrane. Figure S14E does not currently show the protein.

<u>*Response to Reviewer 2#2:*</u> We now added a new Fig. S15 that schematizes both the positions of the N and C-terminal of TMEM214 in relation to the nuclear pore.
