# Supplementary material for: Screening of amphipathic helices identifies features linked to inner nuclear membrane properties
Source: Nat Commun. 2026 Jul 30;17:9316. doi: 10.1038/s41467-026-75561-0 (PMC13530154; doi:10.1038/s41467-026-75561-0)
Supplement: Supplementary file 3 [file 41467_2026_75561_MOESM3_ESM.pdf]

nature portfolio

Corresponding author(s): Shirin Bahmanyar

Last updated by author(s): May 22, 2026

Reporting Summary

Nature Portfolio wishes to improve the reproducibility of the work that we publish. This form provides structure for consistency and transparency in reporting. For further information on Nature Portfolio policies, see our Editorial Policies and the Editorial Policy Checklist.

Statistics

For all statistical analyses, confirm that the following items are present in the figure legend, table legend, main text, or Methods section.

n/a	Confirmed	
☐	☒	The exact sample size (*n*) for each experimental group/condition, given as a discrete number and unit of measurement
☐	☒	A statement on whether measurements were taken from distinct samples or whether the same sample was measured repeatedly
☐	☒	The statistical test(s) used AND whether they are one- or two-sided<br>*Only common tests should be described solely by name; describe more complex techniques in the Methods section.*
☐	☒	A description of all covariates tested
☐	☒	A description of any assumptions or corrections, such as tests of normality and adjustment for multiple comparisons
☐	☒	A full description of the statistical parameters including central tendency (e.g. means) or other basic estimates (e.g. regression coefficient) AND variation (e.g. standard deviation) or associated estimates of uncertainty (e.g. confidence intervals)
☐	☒	For null hypothesis testing, the test statistic (e.g. F, t, r) with confidence intervals, effect sizes, degrees of freedom and P value noted<br>*Give P values as exact values whenever suitable.*
☒	☐	For Bayesian analysis, information on the choice of priors and Markov chain Monte Carlo settings
☒	☐	For hierarchical and complex designs, identification of the appropriate level for tests and full reporting of outcomes
☐	☒	Estimates of effect sizes (e.g. Cohen's *d*, Pearson's *r*), indicating how they were calculated

Our web collection on statistics for biologists contains articles on many of the points above.

Software and code

Policy information about availability of computer code

Data collection	MemBrain data were downloaded from http://www.csbio.sjtu.edu.cn/bioinf/MemBrain/; PMIpred (no versions) (van Hilten et al. 2024) was used to collect ddF values for AHs; HeliQuest version 2 (Gautier et al. 2008; https://heliquest.ipmc.cnrs.fr/) was used for collecting AH parameters; AlphaFold predicted structure data were downloaded from AlphaFold Protein Structure Database model v4 (https://alphafold.com/)
Data analysis	Publicly available softwares used in this study are:<br>FIJI version 2.16(Schindelin et al., 2012), https://imagej.net/Fiji;<br>GraphPad Prism version 9 and version 10 (GraphPad Software), https://www.graphpad.com/scientificsoftware/prism/;<br>SnapGene version 8.0 (GSL Biotech LLC), https://www.snapgene.com/;<br>TMHMM version 2.0 (Krogh et al., 2001), https://services.healthtech.dtu.dk/service.php?TMHMM-2.0;<br>HeliQuest version 2 (Gautier et al., 2008), https://heliquest.ipmc.cnrs.fr/;<br>BeStSel version 2024 (Micsonai et al. 2015), https://bestsel.elte.hu/index.php;<br>Python version 3.8 or 3.11 (Python Software Foundation), https://www.python.org/;<br>Anaconda Navigator version 2.6.3 (Anaconda Inc), https://www.anaconda.com/products/distribution;<br>PyMol version 3.1 (Schrödinger, LLC), https://www.pymol.org/;<br>PAE viewer (no version), (Elfmann C, Stülke J. 2023) https://pae-viewer.uni-goettingen.de/<br><br>Custom python and ImageJ Macro scripts used in this study are available on GitHub as listed below (also posted on Zenodo) and cited in the Method section with DOI.<br>https://github.com/shokenlee/Nuclear_proteome; https://github.com/shokenlee/Find-AH-NE_MemBrain;

https://github.com/shokenlee/AH_AAcomposition; https://github.com/shokenlee/ImageAnalysis_Lee_etal_2026

For manuscripts utilizing custom algorithms or software that are central to the research but not yet described in published literature, software must be made available to editors and reviewers. We strongly encourage code deposition in a community repository (e.g. GitHub). See the Nature Portfolio guidelines for submitting code & software for further information.

Data

Policy information about availability of data

All manuscripts must include a data availability statement. This statement should provide the following information, where applicable:

- Accession codes, unique identifiers, or web links for publicly available datasets
- A description of any restrictions on data availability
- For clinical datasets or third party data, please ensure that the statement adheres to our policy

All data supporting the findings of the study are available in this paper. All source data for the graphs and unprocessed blots are also available. All raw image data for the representative images shown in the figures and the raw sequence data for TMEM214 KO cells are available on Figshare (https://doi.org/10.6084/m9.figshare.32608785). Other raw data generated in this study are available upon request to the corresponding author.

Research involving human participants, their data, or biological material

Policy information about studies with human participants or human data. See also policy information about sex, gender (identity/presentation), and sexual orientation and race, ethnicity and racism.

Reporting on sex and gender	Not applicable
Reporting on race, ethnicity, or other socially relevant groupings	Not applicable
Population characteristics	Not applicable
Recruitment	Not applicable
Ethics oversight	Not applicable

Note that full information on the approval of the study protocol must also be provided in the manuscript.

Field-specific reporting

Please select the one below that is the best fit for your research. If you are not sure, read the appropriate sections before making your selection.

[x] Life sciences [ ] Behavioural & social sciences [ ] Ecological, evolutionary & environmental sciences

For a reference copy of the document with all sections, see nature.com/documents/nr-reporting-summary-flat.pdf

Life sciences study design

All studies must disclose on these points even when the disclosure is negative.

Sample size	No sample size calculation was performed and sample size was determined based on previous experience in the lab.
Data exclusions	No data was excluded from analysis
Replication	AH-mNG live imaging screen, AH-mNG-NLS NE enrichment score measurement, hypotonic and cell-stretch experiments, western blotting experiments were performed in biological duplicates. GUV and circular dichroism experiments were performed in two or three independent experiments, as denoted in the figure legends.
Randomization	Randomization in the allocation of samples was not performed because appropriate controls were used, and both control and experimental groups were prepared in the same manner.
Blinding	Blind image analysis was performed for categorizing AH-mNG localization patterns. For other experiments, blinding was not performed because appropriate controls were used, and each experiment received equal treatment.

Reporting for specific materials, systems and methods

We require information from authors about some types of materials, experimental systems and methods used in many studies. Here, indicate whether each material, system or method listed is relevant to your study. If you are not sure if a list item applies to your research, read the appropriate section before selecting a response.

Materials & experimental systems

n/a	Involved in the study
	☒ Antibodies
	☒ Eukaryotic cell lines
☒	Palaeontology and archaeology
☒	Animals and other organisms
☒	Clinical data
☒	Dual use research of concern
☒	Plants

Methods

n/a	Involved in the study
☒	ChIP-seq
☒	Flow cytometry
☒	MRI-based neuroimaging

Antibodies

Antibodies used: Mouse anti-mNeonGreen [32F6] (ChromoTek Cat# 32f6-100, RRID:AB_2827566); Mouse anti-alpha-tubulin [DM1a] (Millipore Cat# 05-829, RRID:AB_310035); Rabbit polyclonal anti-TMEM214 (Thermo Fisher Scientific Cat#: PA5-98198, RRID:AB_2812811); Mouse anti-Nuclear Pore Complex Proteins [mAb414] (BioLegend Cat# 902901, RRID:AB_2565026)

Validation: Antibodies were validated by manufacturers and previous literature. Validation data and list of papers validating the antibodies are found in the manufacturers' websites.

Eukaryotic cell lines

Policy information about cell lines and Sex and Gender in Research

Cell line source(s): U2OS cells from Frank Slack lab (Harvard Medical School); TMEM214 KO U2OS cell lines were generated and validated in this study; HeLa cells from Single Molecule Biophotonics group at ICFO (The Institute of Photonic Sciences); MEF from Nadya Dimitrova Lab (Yale University); COS-7 from Bewersdorf lab (Yale School of Medicine)

Authentication: No cell line has been authenticated

Mycoplasma contamination: Cells lines tested negative for mycoplasma contamination frequently using a PCR-based method.

Commonly misidentified lines (See ICLAC register): No cell line from the list of cross-contaminated or otherwise misidentified cell lines was used.

Plants

Seed stocks: *Report on the source of all seed stocks or other plant material used. If applicable, state the seed stock centre and catalogue number. If plant specimens were collected from the field, describe the collection location, date and sampling procedures.*

Novel plant genotypes: *Describe the methods by which all novel plant genotypes were produced. This includes those generated by transgenic approaches, gene editing, chemical/radiation-based mutagenesis and hybridization. For transgenic lines, describe the transformation method, the number of independent lines analyzed and the generation upon which experiments were performed. For gene-edited lines, describe the editor used, the endogenous sequence targeted for editing, the targeting guide RNA sequence (if applicable) and how the editor was applied.*

Authentication: *Describe any authentication procedures for each seed stock used or novel genotype generated. Describe any experiments used to assess the effect of a mutation and, where applicable, how potential secondary effects (e.g. second site T-DNA insertions, mosiacism, off-target gene editing) were examined.*
