# Supplementary material for: Screening of amphipathic helices identifies features linked to inner nuclear membrane properties
Source: Nat Commun. 2026 Jul 30;17:9316. doi: 10.1038/s41467-026-75561-0 (PMC13530154; doi:10.1038/s41467-026-75561-0)
Supplement: Supplementary file 4 [file 41467_2026_75561_MOESM4_ESM.pdf]

Description of Additional Supplementary Files

Supplementary Data 1

Description: List of 39 human proteins with UniProt Entries, which resulted from validation of the Supplementary Data8 5 in Schirmer et al. 2003 (ref. 44) with latest NCBI and UniProt records.

Supplementary Data 2

Description: List of 139 human proteins from Supplementary Data5 in Korfali et al. 2010 (ref. 45).

Supplementary Data 3

Description: List of 119 human proteins from Supplementary Data4 in Korfali et al. 2012 (ref. 47).

Supplementary Data 4

Description: List of 94 human proteins from Supplementary Data6 in Wilkie et al. 2011 (ref. 46).

Supplementary Data 5

Description: List of 242 human proteins from Supplementary Data3 in Cheng et al. 2019 (ref. 48).

Supplementary Data 6

Description: List of the 405 proteins that resulted from merging the Tables S1-S5 11.

Supplementary Data 7

Description: 3,077 proteins were found to contain "nucle" in subcellular location information with its information fully/partially "validated" OR "supported".

Supplementary Data 8

Description: List of the 286 NE proteins.

Supplementary Data 9

Description: 2,733 metazoan proteins were found from the 11,750 proteins for which MemBrain predicted the presence/absence of AH.

Supplementary Data 10

Description: 1,862 human proteins from MemBrain.

Supplementary Data 11

Description: The 86 NE proteins with one or two predicted AH(s).

Supplementary Data 12

Description: The 33 NE proteins without a predicted AH.

Supplementary Data 13

Description: Assessment of AHs in Supplementary Data11 narrowed them down to 62 novel AH candidates from 51 proteins.

Supplementary Data 14

Description: List of 57 AH sequences that were tested by live cell imaging of AH appended with mNG. Visual pattern of subcellular localization is noted.

Supplementary Data 15

Description: Properties of the AH candidates such as net charge.

Supplementary Data 16

Description: Median values of the NE enrichment scores and amino acid properties.

Supplementary Data 17

Description: PMIpred values and other data for AHs included.
