# Supplementary material for: Screening of amphipathic helices identifies features linked to inner nuclear membrane properties
Source: Nat Commun. 2026 Jul 30;17:9316. doi: 10.1038/s41467-026-75561-0 (PMC13530154; doi:10.1038/s41467-026-75561-0)
Supplement: Supplementary file 5 [file 41467_2026_75561_MOESM6_ESM.zip › Source Data/Rawblots.pdf]

Fig. S5

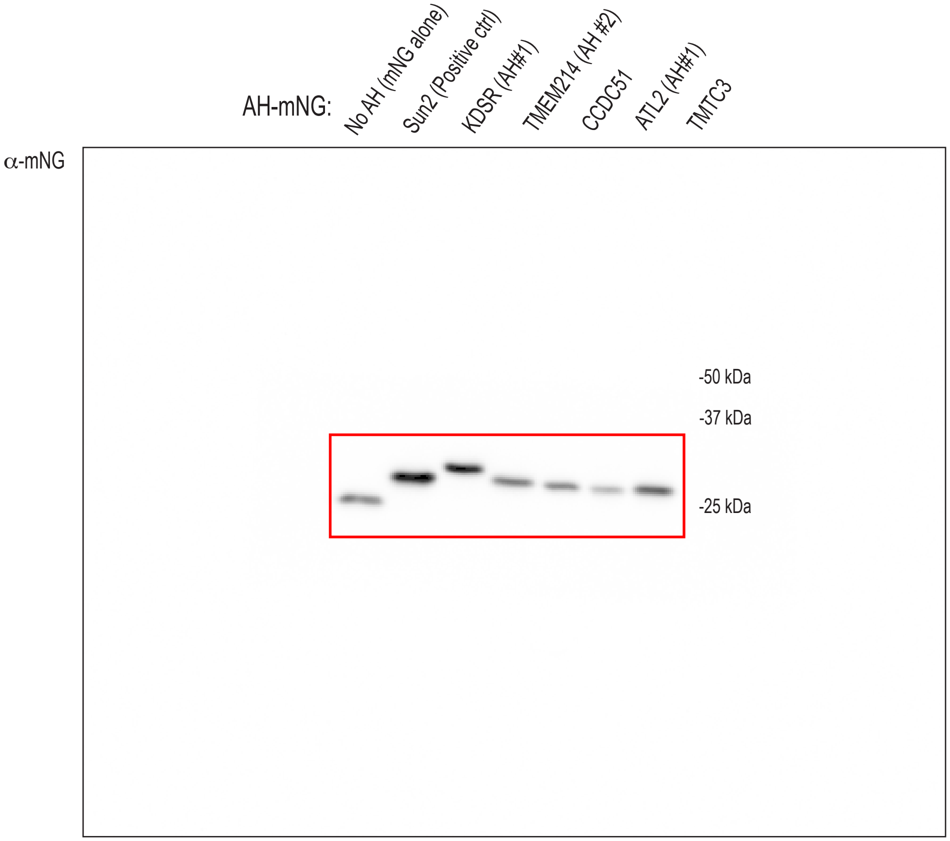
AH-mNG:
No AH (mNG alone)
Sun2 (Positive ctrl)
KDSR (AH#1)
TMEM214 (AH #2)
CCDC51
ATL2 (AH#1)
TMTC3
α-mNG
-50 kDa
-37 kDa
-25 kDa

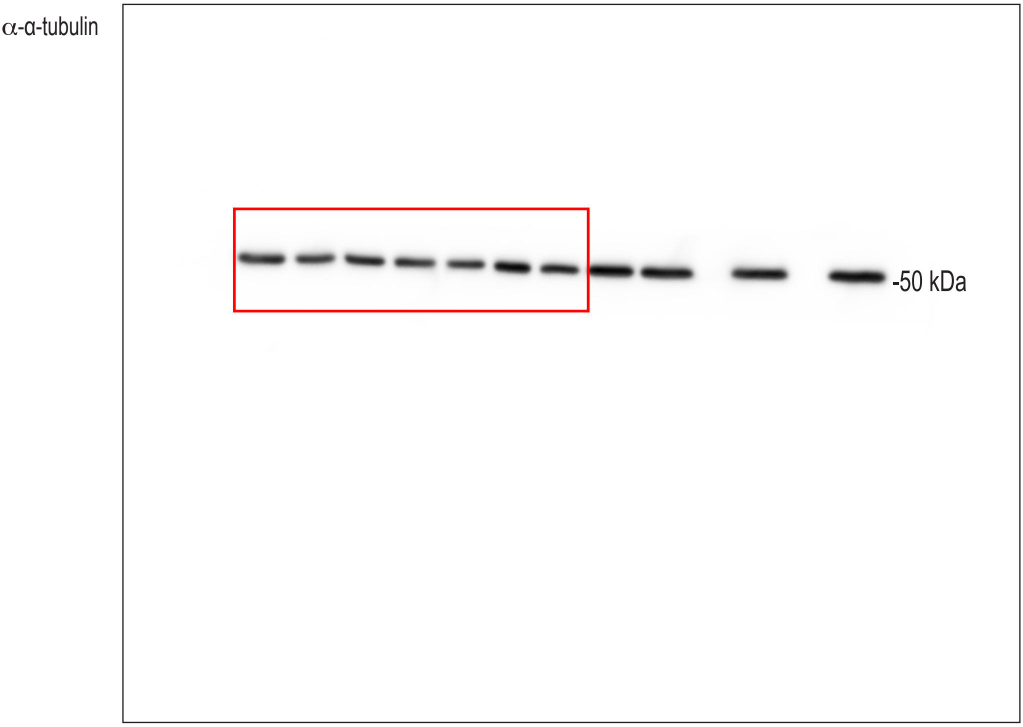
α-α-tubulin
-50 kDa

Fig. S5

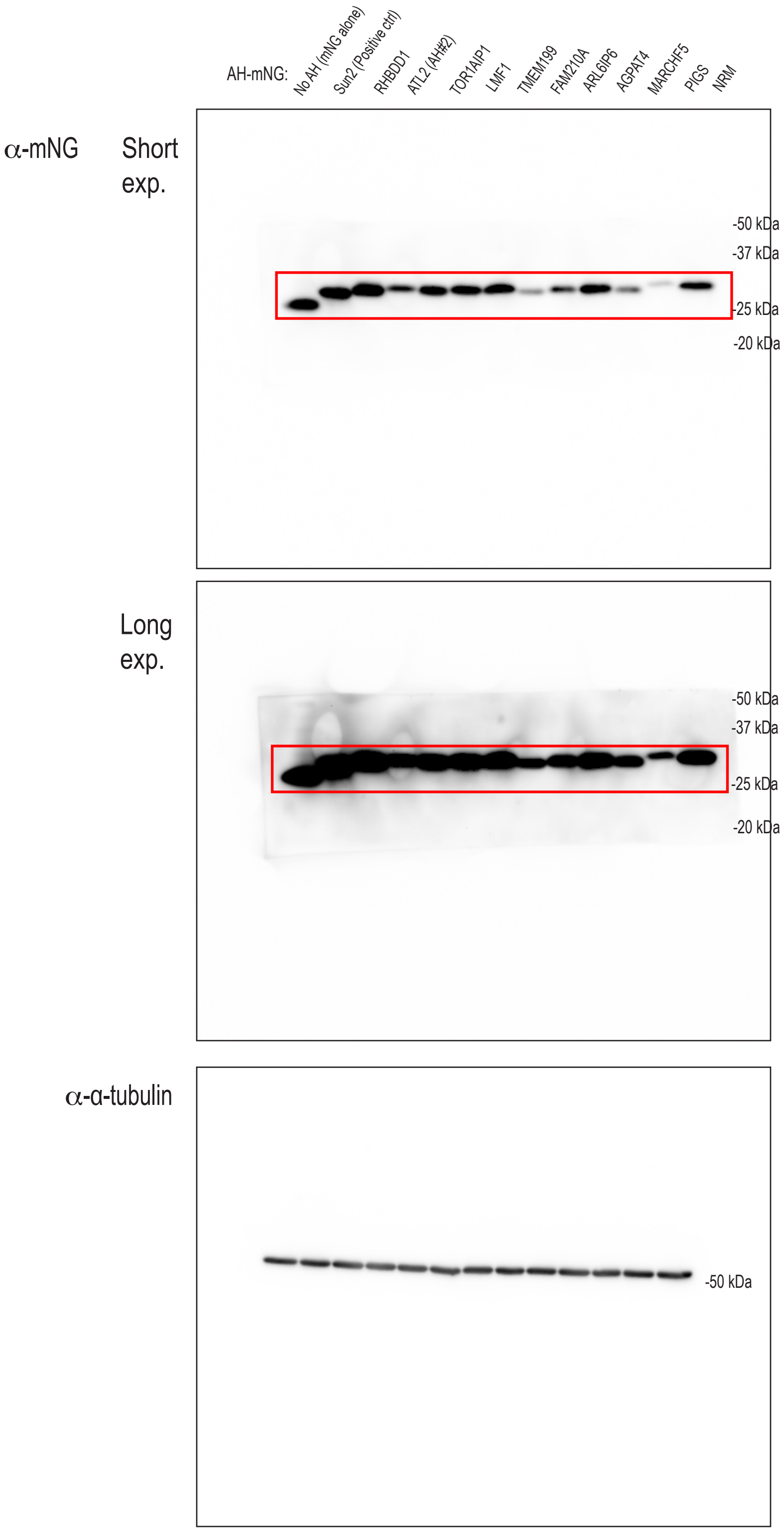
AH-mNG:
No AH (mNG alone)
Sun2 (Positive ctrl)
RHBDD1
ATL2 (AH#2)
TOR1AIP1
LMF1
TMEM199
FAM210A
ARL6IP6
AGPAT4
MARCHF5
PIGS
NRM
α-mNG
Short exp.
-50 kDa
-37 kDa
-25 kDa
-20 kDa
Long exp.
-50 kDa
-37 kDa
-25 kDa
-20 kDa
α-α-tubulin
-50 kDa

Fig. S9e

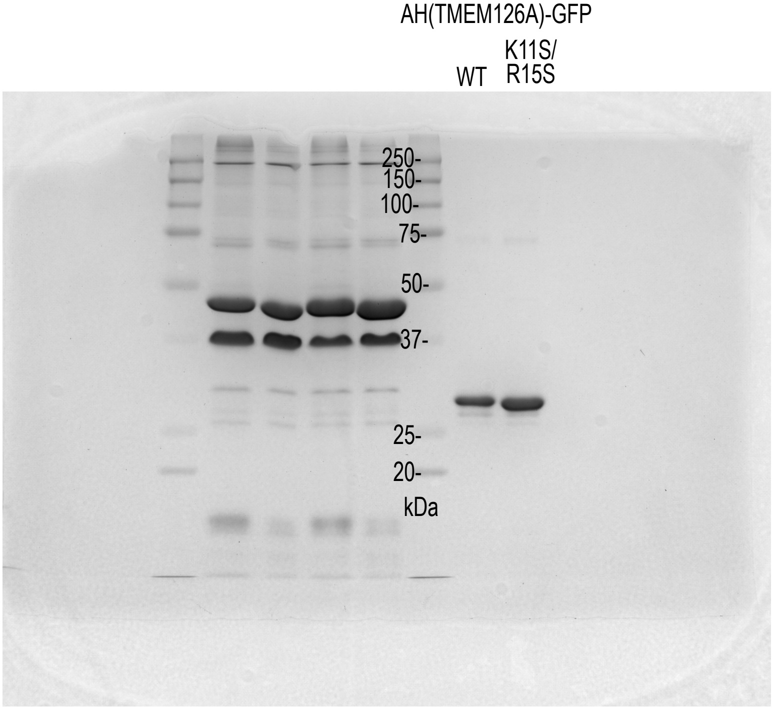

Fig. S14a

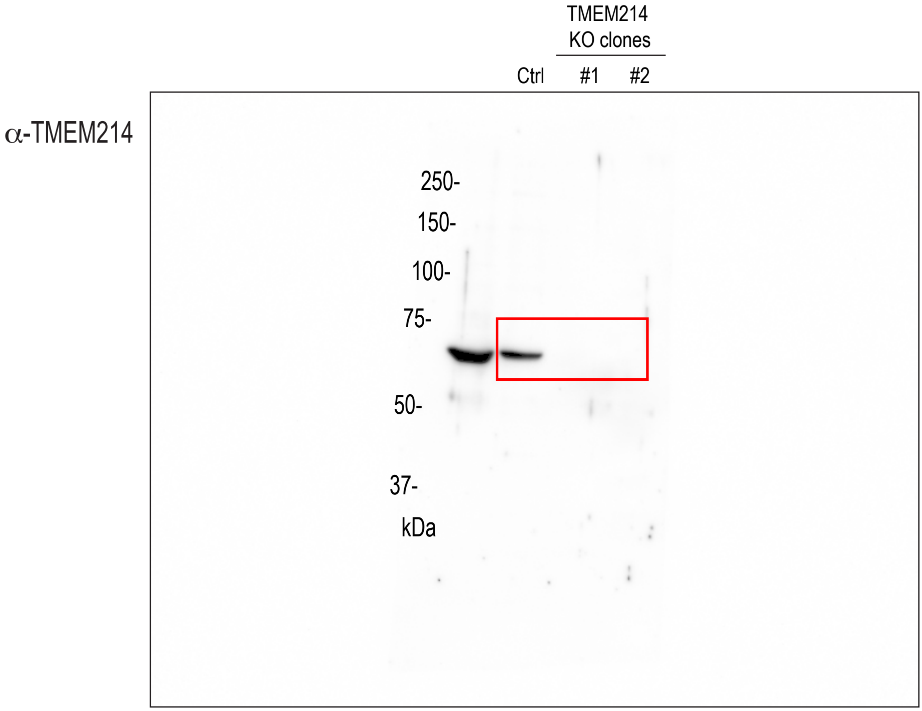
TMEM214
KO clones
Ctrl
#1
#2
α-TMEM214
250-
150-
100-
75-
50-
37-
kDa

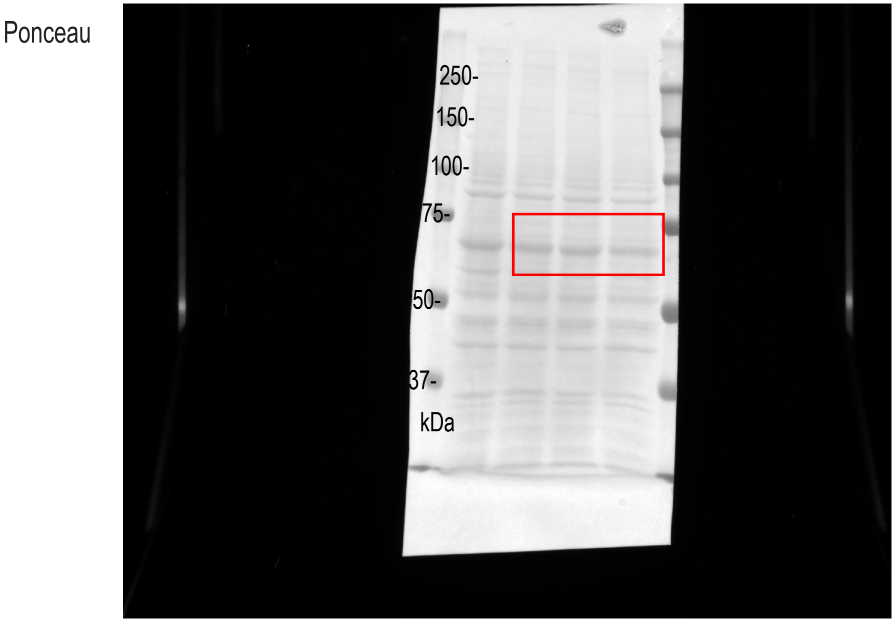
Ponceau
250-
150-
100-
75-
50-
37-
kDa
